# Assessing corporate sustainability with large language models: evidence from Europe

**DOI:** 10.1038/s41467-026-75160-z

**Published:** 2026-07-07

**Authors:** Kerstin Forster, Lucas Keil, Victor Wagner, Maximilian A. Müller, Thorsten Sellhorn, Stefan Feuerriegel

**Affiliations:** 1 LMU Munich, Munich, Germany; 2 Munich Center for Machine Learning, Munich, Germany; 3 University of Cologne, Cologne, Germany; 4 Sustainability Reporting Navigator, Munich, Germany; 5 Stockholm School of Economics, Stockholm, Sweden

**Keywords:** Sustainability, Business, Social sciences

## Abstract

Companies play a crucial role in achieving global sustainability goals, yet evidence on their progress across environmental, social, and governance (ESG) dimensions remains limited. We develop a machine learning framework to systematically extract ESG indicators from corporate reports. Applying this approach to annual and sustainability reports of 600 large European firms (2014–2023), we construct a dataset of 2.9 million ESG observations across environmental, social, and governance topics. We assess ESG transparency based on disclosures aligned with the European Sustainability Reporting Standards (ESRS) and evaluate ESG performance using extracted numerical indicators. Results reveal a pronounced transparency gap: firms in the top ESG rating decile disclose 22% more indicators than those in the bottom decile, although this gap narrows over time. Performance trends are uneven: while most social indicators remain largely stagnant, except for gains in gender equality, environmental indicators show some improvement. Reported scope 3 emissions increase sharply, largely reflecting improved disclosure. Our open-source framework enables systematic tracking of corporate ESG efforts.

## Abstract (web summary)

This study uses machine learning to extract environmental, social, and governance (ESG) indicators from corporate reports of 600 large European firms, revealing a pronounced but narrowing disclosure gap and uneven ESG performance.

## Introduction

Private-sector businesses importantly shape the world’s progress toward environmental, social, and governance (ESG) goals^1–5^. A total of 169 companies are responsible for 80 percent of industrial GHG emissions, of which 56 are European^6^. Yet, despite widespread corporate pledges to reduce environmental footprints^7,8^, many companies still lag behind their environmental goals^2,3^. Similarly, corporate efforts to improve workplace diversity also continue to fall short^9,10^. Clearly, corporate ESG efforts need close monitoring, if performance improvements are to be achieved.

However, comprehensive evidence about corporate progress along ESG dimensions is limited, primarily for five reasons. First, many studies focus narrowly on a few ESG dimensions such as carbon emissions, energy use, or water withdrawal^11–16^, while neglecting other environmental (e.g., biodiversity) as well as social and governance indicators (e.g., employee turnover). Second, others are confined to specific sectors^4,17^, countries^18,19^, or time periods^20–22^, reducing the ability to track broader cross-sectoral trends or identify regional disparities. Third, several studies analyze the narrative in corporate sustainability reports qualitatively^23–26^, lacking quantifiable data needed for tracking progress in ESG performance. Fourth, while there is a large body of research in finance, accounting, and legal studies using natural language processing to analyze regulatory filings (see overviews in refs. ^27^ and^28^), primarily for the purpose of text classification (e.g., sentiment analysis, readability analysis), monitoring ESG performance is a different task and requires the extraction of structured quantitative information. Fifth, the most comprehensive ESG data is compiled by commercial providers (see Supplementary Information S2); however, because no unified reporting standard exists, these datasets are costly, focused on investors’ information needs, and often report inconsistent measures—unless these measures come directly from the companies themselves^29^. Given these limitations, a comprehensive, quantitative assessment of corporate ESG transparency and performance across granular ESG dimensions and over a multi-year trajectory is missing. Here, we aim to fill this gap.

**Fig. 1:**
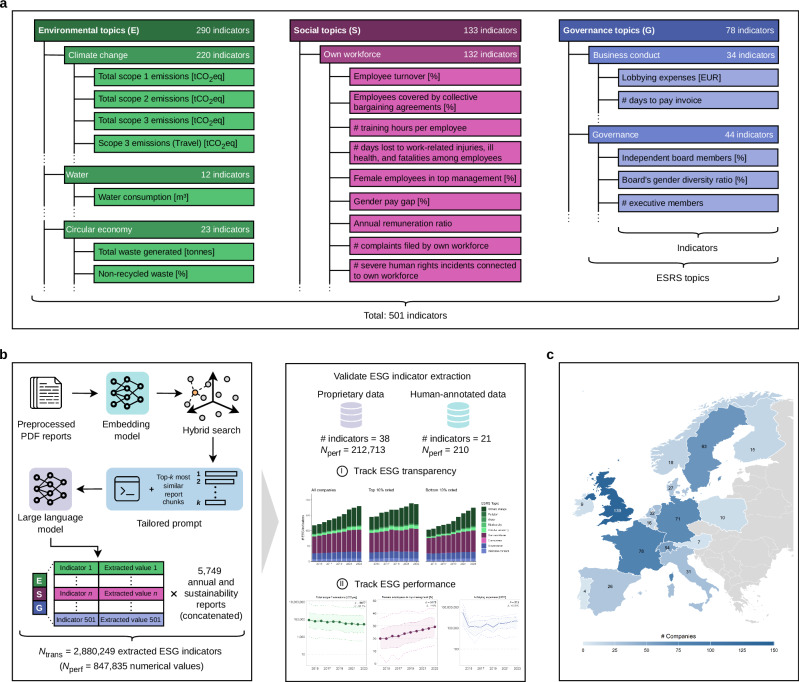
Machine learning framework to extract environmental, social, and governance (ESG) indicators from corporate reports. **a** We assess corporate sustainability in Europe between 2014 and 2023 in accordance with European Sustainability Reporting Standards (ESRS)^35^. Our framework includes 501 quantitative ESG indicators across environmental (E: 290), social (S: 133), and governance (G: 78) topics. Details on ESRS are provided in Supplementary Information S1. **b** Our machine learning framework is prompted to return *N*_trans_ = 2, 880, 249 ESG indicators from corporate reports. Out of these, we successfully identify and extract numerical values for ${N}_{{{\rm{perf}}}}=847,835$ indicators. Here, *N*_trans_ refers to how often we prompt the large language model, while ${N}_{{{\rm{perf}}}}$ denotes how often these prompts resulted in a numerical value because the ESG indicator was actually disclosed in the corporate reports. Our framework comprises two major components: (i) retrieval-augmented generation to extract ESG indicators from corporate reports using a large language model; and (ii) validation by comparing the extracted numerical values against proprietary and human-annotated ESG data. We then use our machine learning framework to analyze ESG-related transparency and performance across nine distinct ESRS topics based on the extracted indicators. Icons were obtained from Flaticon (https://www.flaticon.com/): file by iconixar, neural network by Vectors Tank, window by LAFS, database by Stockio. **c** The map shows the number of companies by country, with darker shading indicating more companies. Overall, our dataset includes the 600 largest listed companies in Europe across 16 countries. The full list of companies is provided in Supplementary Table S1.

Targeted transparency—i.e., disclosure requirements aimed at empowering civil society to hold companies accountable^30^—allows for tracking progress along ESG dimensions^31^ and can even act as a catalyst for change^32,33^. Policy-makers rely on ESG-related transparency to inform regulatory frameworks and monitor compliance with sustainability standards. For example, the EU’s recent Corporate Sustainability Reporting Directive (CSRD)^34^ mandates the most comprehensive sustainability reporting requirements globally. It introduced a set of European Sustainability Reporting Standards (ESRS)^35^ to unify ESG reporting for large, listed companies (see Fig. 1a and Supplementary Information S1). Financial stakeholders, including banks, insurers, and asset managers, increasingly seek to align their portfolios with sustainability goals^36^ and thus depend on reliable ESG information to assess and manage risks and returns. Societal actors, such as consumers, non-governmental organizations (NGOs), and employees, rely on such information to hold corporations accountable for their ESG performance^37^. While transparency alone does not guarantee improved ESG outcomes, comprehensive and gap-free ESG reporting is often a necessary first step to help align corporate practices and global ESG goals^13^.

Here, we develop a machine learning (ML) framework to construct a time series of ESG indicators from corporate reports based on retrieval-augmented generation (RAG) (Fig. 1). We then track the values of these ESG indicators along environmental (e.g., scope 1, 2, and 3 GHG emissions, water consumption, waste), social (e.g., employee turnover, women in top management, gender pay gap), and governance (e.g., lobbying expenses) topics as specified by ESRS. Our analysis covers the 600 largest listed corporations in Europe, which represent nearly 90% of the investable equity market in Europe based on market capitalization, over the 2014–2023 period. Overall, we prompt our ML framework for *N*_trans_ = 2, 880, 249 ESG indicators, of which ${N}_{{{\rm{perf}}}}=847,835$ return a numerical value because the corresponding ESG indicator was disclosed by the company and can be extracted from the corporate report. Notably, while we only extract values for 29% of the indicators, this does not imply that 71% are missing; companies might not disclose data on certain indicators because they refer to a topic that is not deemed relevant (i.e., material) to the sector or entity.

We use this dataset for two key analyses: First, we assess ESG-related transparency as the availability of ESG disclosures defined by ESRS. Second, we analyze ESG performance over time and across industries based on the values of these indicators. To demonstrate the scientific value of our generated dataset for downstream analysis, we report descriptive evidence but refrain from causal claims about the drivers of transparency or performance. We make both our ML framework and our dataset publicly available to allow policy-makers, investors, and other societal actors to systematically monitor and compare corporate ESG efforts—including among industry peers and against companies’ publicly stated targets. Doing so should help drive corporate progress toward global sustainability goals.

## Results

### ML framework overview

Our end-to-end ML framework operates as follows (Fig. 1b; see the “Methods” and the Supplementary Information for full technical details). For each company–year (i.e., each company observed in a given year), we collect the corresponding annual and sustainability reports (as PDFs), extract and clean the text from the PDFs, split the text into overlapping chunks, and index the chunks in a vector database. Within each company–year, we then query the indexed text separately for each of the 501 ESG indicators, retrieve and re-rank candidate chunks, and use a state-of-the-art LLM to extract the requested numeric value (or abstain if absent). Finally, we standardize units and currencies and store the resulting output in a structured format (Supplementary Fig. S4). We apply this framework to STOXX Europe 600 index constituents (as of 2023) over the 2014–2023 period. Key methodological limitations are discussed in the Supplementary Information S4.

We validated the outputs against (i) a proprietary benchmark dataset and (ii) expert human annotations on a subset. The agreement is strong against both comparisons (Supplementary Fig. S1–S2), which implies that the generated dataset is reliable for the descriptive analyses below. The full validation design and error analysis are described in Methods section under Validation.

While our framework enables large-scale, automated ESG indicator extraction, the reliability of its outputs depends on the quality of extraction at each stage of the pipeline. We therefore quantify how observations progress through each stage of the ML pipeline and report the results at both the company–year level and the indicator–company–year level, which allows us to identify at which stage information enters—or drops out of—the process (Supplementary Fig. S6). Building on this decomposition, an indicator may fail to be retrieved for two reasons: (i) it may not be disclosed (e.g., due to non-materiality or because the company reports a different indicator or uses a different reporting variant), or (ii) the extraction may fail (e.g., due to ambiguous reporting). To assess whether the extraction may further vary across indicators, we additionally report per-indicator disclosure detection and standardization rates (Supplementary Table S6).

### ESG-related transparency

We use our ML framework to track ESG indicators from both the annual and sustainability reports of the 600 major European companies listed in the STOXX Europe 600 (as of 2023) for the time period 2014 through 2023 (Fig. 1b). These companies cover nearly 90% of the investable equity market in Europe and span 16 European countries, including the UK (139 companies), France (78), and Germany (71) (Fig. 1c). The full list of all companies is provided in Supplementary Table S1. From each report, we extract reported ESG indicators from among the 501 indicators defined by European Sustainability Reporting Standards (ESRS)^35^ (see Supplementary Information S1 for background). ESRS are structured along over-arching topics (e.g., climate change, own workforce, business conduct), each mandating a granular set of quantitative ESG indicators that collectively constitute the 501 indicators. We track these indicators to determine: (1) whether the indicator is present or absent from the corporate report (*transparency*), and, if present, (2) its numerical value (*performance*). Accordingly, this enables systematic benchmarking across firms, sectors, and time, which helps generate descriptive evidence, but without causal claims, to inform hypothesis development and demonstrate the scientific value of the dataset.

**Fig. 2:**
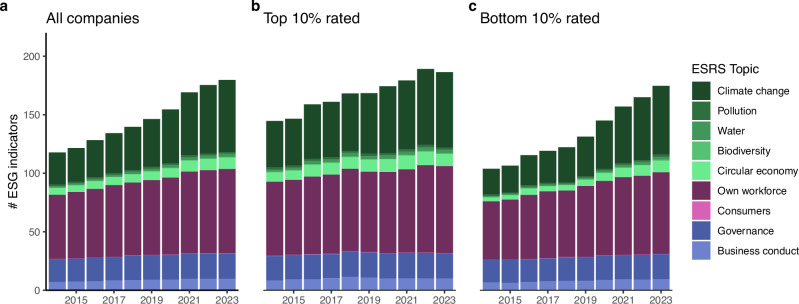
Transparency gap between average and top-in-class companies. **a** Shown is the average number of ESG indicators disclosed annually, grouped by ESRS topic. Of note, the number of mandated ESG indicators varies across sectors due to differences in business model, resource use, and regulatory exposure. To assess the transparency gap, we compare the average against companies that are **b** higher-rated and **c** lower-rated in terms of sustainability performance. Specifically, we compare against companies for which the ESG rating (based on lagged MSCI ESG ratings^38^) ranks in the top − 10% and bottom − 10%, respectively. Deciles are calculated on a yearly basis. This analysis shows how top- and bottom-rated companies compare in ESG disclosure relative to the full sample.

Our analysis shows an overall trend toward increased transparency (Fig. 2a). The average number of disclosed ESG indicators increased from 117.8 (2014) to 179.7 (2023;  + 52.5%), with more pronounced increases in specific topics such as climate change ( + 125.0%), water ( + 83.0%), and circular economy ( + 73.0%). Notably, the number of reported ESG indicators varies across industry sectors due to differences in business model, resource intensity, and regulatory exposure. But even beyond structural sector differences, our analysis points to a notable transparency gap (Fig. 2b, c). In 2023, companies with a top − 10% ESG rating (based on external data providers^38^) disclosed on average 186.5 ESG indicators, compared to an average of 174.7 indicators for companies that are lagging behind, corresponding to a 6.8% difference. This gap was substantially wider in 2014, when companies in the upper decile reported on average 144.7 indicators versus just 103.8 for the bottom decile (difference: 39.4%). This narrowing of the transparency gap over the last decade coincides with a general increase in average ESG ratings over this period^38^, suggesting an overall strengthening and alignment of data availability about corporate sustainability.

**Fig. 3:**
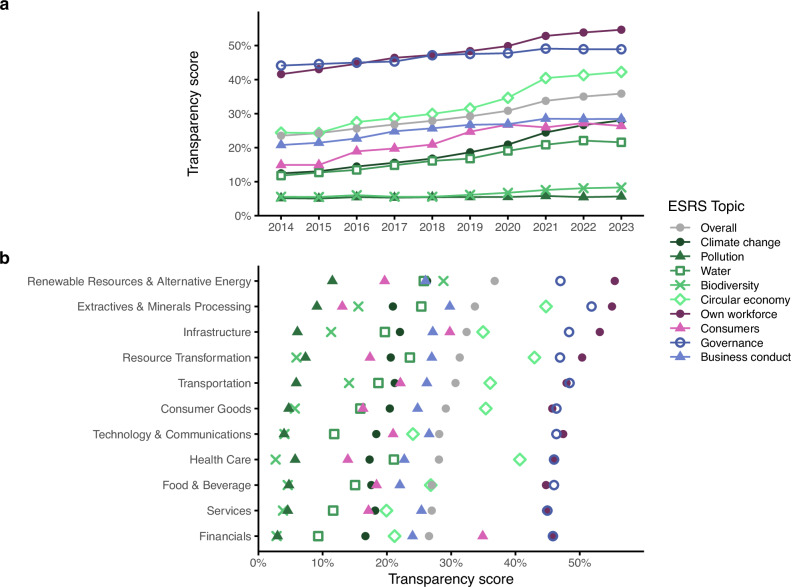
Transparency across ESRS topics and industries. Here, transparency score refers to the relative number of disclosed indicators out of all 501 indicators. **a** reports the transparency scores disclosed by ESRS topic between 2014 and 2023. **b** shows the transparency scores by industry. Industries are categorized according to the Sustainable Industry Classification System (SICS), developed by the Sustainability Accounting Standards Board (SASB) to classify companies into sectors with comparable exposure to sustainability-related risks and opportunities^68^. Industries in (**b**) are sorted by their overall transparency score (descending).

We find substantial disparities in ESG-related transparency across topics and industries, as measured by a transparency score defined as the relative number of disclosed indicators out of all 501 indicators (Fig. 3a, b). This could be explained by differences in the materiality of topics, that is, certain sectors or entities may not be affected by a given topic and therefore do not disclose detailed information, or different scope and depth of disclosure frameworks prior to the ESRS. Overall, transparency scores are unequally distributed across topics (Fig. 3a). In 2023, topics with particularly high transparency scores were own workforce (54.7%), governance (48.9%), and circular economy (42.3%), whereas transparency scores were particularly low for the topics pollution (5.6%) and biodiversity (8.3%). Overall, several topics show increasing transparency scores between 2014 and 2023, such as circular economy (change in transparency score:  + 17.8 percentage points [p.p.]), climate change ( + 15.5 p.p.), and own workforce ( + 13.1 p.p.). There is also some heterogeneous variation in overall as well as topic-specific transparency scores across industries (Fig. 3b). Variation in overall transparency may reflect sector-specific disclosure traditions and peer benchmarking effects, whereby firms adopt similar practices as their industry peers to meet investor and stakeholder expectations. Topic-specific variation might reflect their relative importance across industries. For example, whereas topics like own workforce and governance consistently score highest, arguably due to being universally material and often regulated, the relative importance of other topics varies. One striking example is the financial industry, which displays comparatively lower transparency across environmental topics in contrast with social and governance, consistent with its lower direct ecological footprint but greater exposure to organizational and ethical issues. Still, the relative ranking of topics shows broadly similar characteristics across industries, with one possible explanation for that being a preference of companies to report on commonly accepted, measurable metrics requested by data providers and rating agencies. Further context on ESG transparency trends and the role of rating agencies is provided in Supplementary Information S2.

**Fig. 4:**
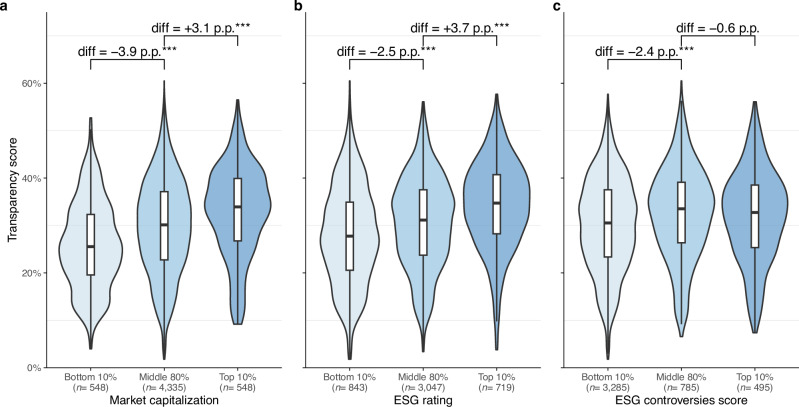
Heterogeneity in ESG-related transparency across company characteristics. Here, we analyze transparency scores (i.e., the relative number of disclosed indicators out of all 501 indicators) by (**a**) market capitalization, **b** ESG rating (based on MSCI ESG ratings^38^ measured annually by company), and **c** ESG controversies score (by Refinitiv^60^; measured annually by company). The ESG controversies score captures exposure to negative ESG-related events and news coverage^39^. All independent variables are lagged by one year to mitigate potential reverse causality. For each, we compare companies in the bottom 10% and top 10% against the middle 80%, calculated on a yearly basis. We inverse-code the ESG controversies score such that companies with more controversies appear in the top 10%. Shown are violin plots, which represent the kernel probability density of the data, together with boxplots displaying the median and interquartile range. Reported below each group is the number of firm-year observations (*n*). Reported above the brackets is the difference (diff) in percentage points (p.p.). Statistical comparisons are based on two-sided *t*-tests (with ^***^ corresponding to the 0.1% significance level). Whiskers indicate the range of non-outlier values, extending to 1.5 times the interquartile range beyond the first and third quartiles.

To identify potential drivers behind ESG transparency, we analyze transparency scores across different company characteristics. For this analysis, we compare companies in the top 10% and bottom 10% by market capitalization and ESG rating. In general, larger companies are expected to be more transparent, as they have more resources and stronger incentives (e.g., public exposure) for ESG reporting. Companies with higher ESG ratings are typically more advanced in their sustainability practices and should thus tend to disclose more. Overall, transparency is indeed lower among smaller companies and those with lower ESG ratings (Fig. 4a,b).

Specifically, companies in the top 10% by market capitalization and ESG rating have significantly higher transparency scores than the middle 80% by  + 3.1 p.p. (*p* < 0.001) and  + 3.7 p.p. (*p* < 0.001), respectively. In contrast, transparency scores are significantly lower for companies in the bottom 10% of market capitalization ( − 3.9 p.p.; *p* < 0.001) and ESG rating ( − 2.5 p.p.; *p* < 0.001) compared to the middle 80%.

Further, we analyze the ESG controversies score, a proprietary metric capturing exposure to negative ESG-related events and news coverage^39^ (Fig. 4c). Companies with higher (i.e., worse) ESG controversies scores are those whose ESG performance has been more contentious in the past. Here, we find that companies in the bottom 10% of the ESG controversies score (i.e., the least controversial) have significantly lower transparency scores than the middle 80% ( − 2.4 p.p.; *p* < 0.001), while the difference for companies in the top 10% is small and not statistically significant ( − 0.6 p.p.). This suggests that companies with low controversy exposure also tend to disclose less, possibly because they face less external pressure to report comprehensively on ESG matters. Overall, these differences translate into substantial transparency gaps between companies, as confirmed in a regression analysis (see Table 1). For example, companies in the top − 10% of ESG ratings have, on average, 22% higher transparency scores than companies in the bottom − 10%, highlighting the substantial transparency gap between the two groups.

**Table 1:** Determinants of transparency

*Panel A: Transparency explained by market capitalization*						
	(1)	(2)	(3)	(4)	(5)	(6)
Constant	0.297***	0.195***				
	(0.003)	(0.012)				
Top − 10% market capitalization	0.031***	0.016	0.022**	0.020*	0.026**	0.018
	(0.009)	(0.009)	(0.008)	(0.008)	(0.008)	(0.011)
Bottom − 10% market capitalization	− 0.039***	− 0.003	− 0.019*	− 0.005	− 0.021**	0.011
	(0.008)	(0.008)	(0.007)	(0.008)	(0.008)	(0.006)
Controls	No	Yes	Yes	Yes	Yes	Yes
Year FE	No	No	Yes	No	—	—
Sector FE	No	No	No	Yes	—	—
Year × Sector FE	No	No	No	No	Yes	Yes
Company FE	No	No	No	No	No	Yes
*N*	5431	4346	4346	4344	4344	4344
Adj. *R*^2^	0.025	0.081	0.188	0.132	0.238	0.604

*Panel B: Transparency explained by ESG rating*						
	(1)	(2)	(3)	(4)	(5)	(6)
Constant	0.305***	0.036				
	(0.003)	(0.061)				
Top − 10% ESG rating	0.037***	0.029***	0.024**	0.027***	0.022**	− 0.006
	(0.007)	(0.007)	(0.008)	(0.007)	(0.007)	(0.008)
Bottom − 10% ESG rating	− 0.025***	− 0.022**	− 0.028***	− 0.015*	− 0.022**	− 0.004
	(0.007)	(0.007)	(0.007)	(0.007)	(0.007)	(0.004)
Controls	No	Yes	Yes	Yes	Yes	Yes
Year FE	No	No	Yes	No	—	—
Sector FE	No	No	No	Yes	—	—
Year × Sector FE	No	No	No	No	Yes	Yes
Company FE	No	No	No	No	No	Yes
*N*	4609	4346	4346	4344	4344	4344
Adj. *R*^2^	0.033	0.052	0.185	0.114	0.243	0.603

*Panel C: Transparency explained by ESG controversies score*						
	(1)	(2)	(3)	(4)	(5)	(6)
Constant	0.325***	− 0.015				
	(0.005)	(0.062)				
Top − 10% ESG controversies score	− 0.006	0.001	− 0.001	− 0.001	− 0.003	− 0.002
	(0.007)	(0.006)	(0.006)	(0.006)	(0.006)	(0.005)
Bottom − 10% ESG controversies score	− 0.024***	− 0.010	− 0.009	− 0.009	− 0.008	− 0.005
	(0.005)	(0.005)	(0.005)	(0.005)	(0.005)	(0.003)
Controls	No	Yes	Yes	Yes	Yes	Yes
Year FE	No	No	Yes	No	—	—
Sector FE	No	No	No	Yes	—	—
Year × Sector FE	No	No	No	No	Yes	Yes
Company FE	No	No	No	No	No	Yes
*N*	4565	4346	4346	4344	4344	4344
Adj. *R*^2^	0.010	0.088	0.194	0.144	0.250	0.603

**Notes:** This table reports regression estimates where the dependent variable is the company-level transparency score, calculated as the number of reported ESG indicators divided by all indicators listed in ESRS. Each panel uses top − 10% and bottom − 10% dummies of the focal determinant as regressors. Deciles are calculated on a yearly basis. Panel A reports results using lagged market capitalization as the main independent variable. Panel B uses the lagged ESG rating from MSCI, and panel C uses the lagged ESG controversies score from Refinitiv (inverse-coded, i.e., higher scores indicate higher controversy exposure). Model (1) includes only the focal determinant dummies and no additional controls. Models (2)-(6) additionally include the remaining two determinants as continuous controls. The models vary by the set of included fixed effects (FE): Model (1) and Model (2) include no fixed effects; Model (3) includes year fixed effects; Model (4) includes sector fixed effects; Model (5) includes year-by-sector fixed effects; and Model (6) includes company fixed effects. Standard errors are clustered by company and reported in parentheses. Significance levels: ***, **, * correspond to 0.1%, 1%, and 5%, respectively. Reported significance levels are based on two-sided t-tests. No multiple-comparison adjustments were applied. Estimates are based on ordinary least squares (OLS) regression. We find that companies in the top − 10% (bottom 10%) of market capitalization, ESG rating, and controversies score have significantly higher (lower) levels of transparency than the average company. For example, according to Model (1), companies in the top − 10% versus bottom − 10% of ESG ratings have transparency scores that are ~22% higher, calculated by comparing fitted transparency levels from the regression model.

In addition to assessing corporate ESG transparency, our framework enables large-scale, granular analysis of ESG performance—that is, the numerical values of disclosed ESG indicators over time. We focus our analysis on a selected set of indicators that are particularly relevant to current public debates and recent policy frameworks (e.g., the Corporate Sustainability Due Diligence Directive^40^, and the Zero Pollution Action Plan^41^). The complete set of indicators is included in our dataset (see Data availability statement) and is accessible through an interactive dashboard at (https://lookerstudio.google.com/s/jUlW6L1X8u8).

### Environmental performance

**Fig. 5:**
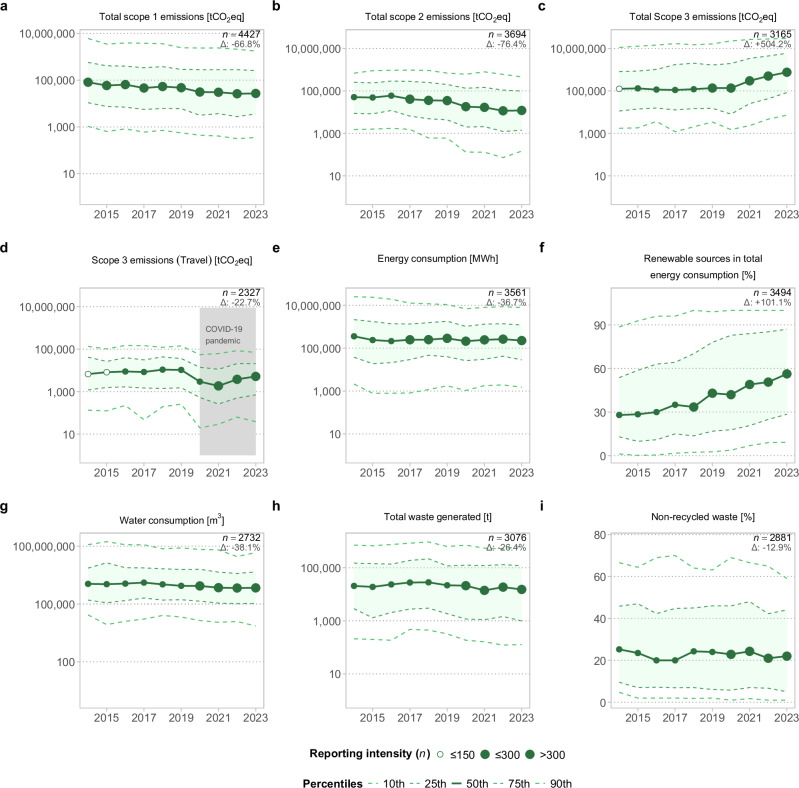
Environmental performance over time. Here, we analyze key environmental indicators between 2014 and 2023. Shown are (**a**) total scope 1 emissions, **b** total scope 2 emissions, **c** total scope 3 emissions, **d** scope 3 emissions (travel), **e** energy consumption, **f** the percentage of renewable sources in total energy consumption, **g** water consumption, **h** total waste generation, and **i** the percentage of non-recycled waste. For each indicator and year, we present the median value across all reporting companies (solid line, 50th percentile), the interquartile range (shaded band, bounded by the 25th and 75th percentiles, inner dashed lines), and the 10th and 90th percentiles (outer dashed lines) to capture the distribution and highlight variation between top and bottom-performing companies. For each panel, dot sizes indicate reporting intensity (i.e., the number of companies disclosing values for each indicator in a given year), *n* denotes the total number of company–year observations for the indicator, and Δ denotes the percentage change in indicator value between 2014 and 2023. Note that logarithmic axes are used to better visualize ESG indicators that span several orders of magnitude. For indicators recorded in percent, we use linear scales. In **d** we also indicate the time period of the COVID-19 pandemic, referring to the World Health Organization’s definition of COVID-19 as a public health emergency of international concern^91^.

Overall, progress toward reducing corporate emissions is mixed (Fig. 5a–d). The graphs show the development of selected indicators over time and provide information on percentiles as well as reporting intensity (i.e., the number of firms reporting on a particular indicator in a given year). For example, median scope 1 emissions (i.e., direct GHG emissions from sources owned or controlled by the company such as production facilities or company vehicles) have declined by 66.8% since 2014 (Fig. 5a), suggesting a gradual shift toward lower direct emissions. Similarly, median scope 2 emissions (i.e., indirect emissions resulting from purchased electricity, steam, heating, or cooling) have declined by 76.4% (Fig. 5b). In contrast, total scope 3 emissions (i.e., indirect emissions across the companies’ upstream and downstream value chain), remained largely stagnant between 2014 and 2020, before increasing by a factor of 5.6 through 2023 (Fig. 5c).

**Fig. 6:**
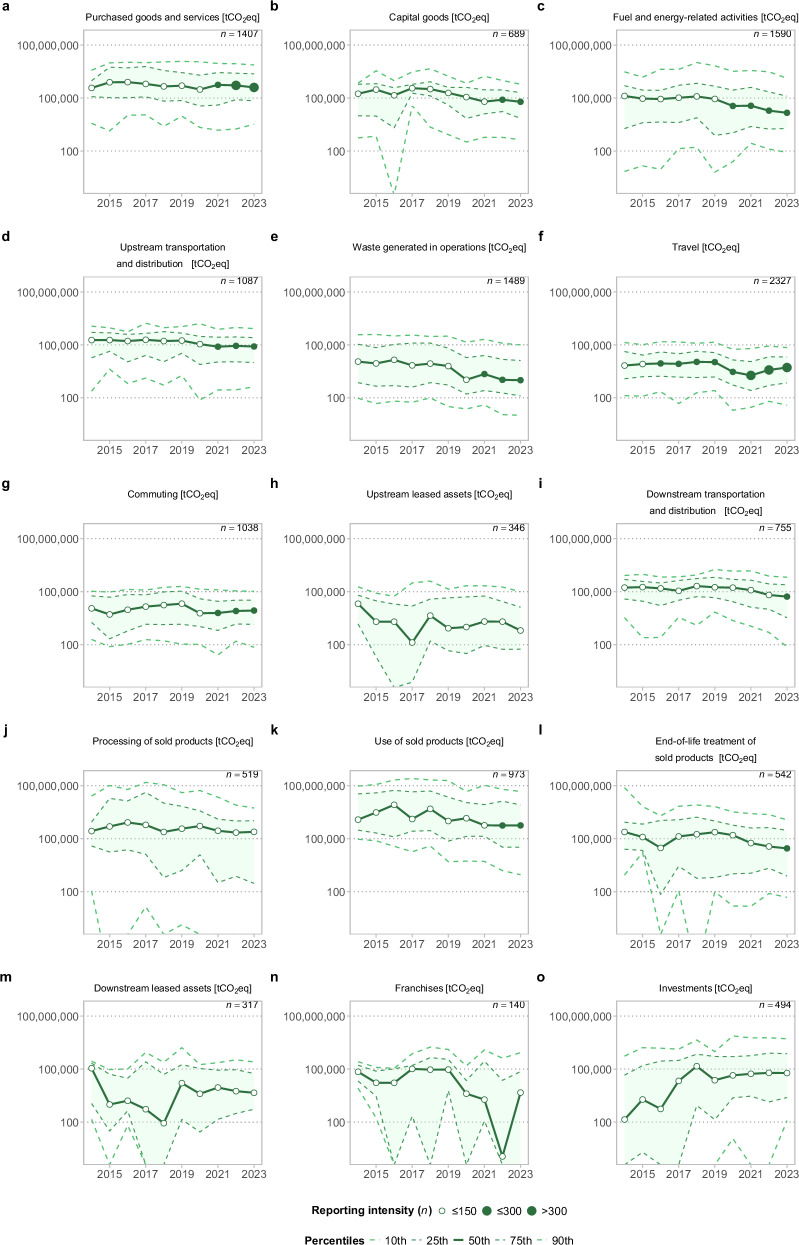
Scope 3 emissions by categories. Here, we analyze subcategories of companies' scope 3 emissions. Shown are scope 3 emissions for (**a**) purchased goods and services, **b** capital goods, **c** fuel and energy-related activities, **d** upstream transportation and distribution, **e** waste generated in operations, **f** travel, **g** commuting, **h** upstream leased assets, (**i**) downstream transportation and distribution, **j** processing of sold products, **k** use of sold products, **l** end-of-life treatment of sold products, **m** downstream leased assets, **n** franchises, and **o** investments. For each indicator and year, we present the median value across all reporting companies (solid line, 50th percentile), the interquartile range (shaded band, bounded by the 25th and 75th percentiles, inner dashed lines), and the 10th and 90th percentiles (outer dashed lines) to capture the distribution and highlight variation between top- and bottom-performing companies. For each panel, dot sizes indicate reporting intensity (i.e., the number of companies disclosing values for each indicator in a given year), and *n* denotes the total number of company–year observations for the indicator. Note that logarithmic axes are used to better visualize different orders of magnitude.

In the specific case of scope 3 emissions, however, we argue that this sharp increase is mainly attributable to higher transparency levels: companies have started to report emissions data for a wider range of previously untracked categories, which mechanically raises total scope 3 emissions. To substantiate this, we inspect the 15 scope 3 categories as defined in the Corporate Value Chain Accounting and Reporting Standard by the GHG Protocol. These exhibit no clear upward trends in individual categories (see Fig. 6) but a stark increase in the number of categories companies report emissions for (see Supplementary Fig. S21)^42^. Additionally, we confirm through regression analysis that the increase in total scope 3 emissions is partly driven by the increase in reported categories (see Supplementary Table S4). As expected, we observe a sharp drop in scope 3 emissions from travel during the COVID-19 pandemic (Fig. 5d). Although travel-related emissions have rebounded by a factor of 2.8 since 2021, they remain below pre-pandemic levels in 2023, which suggests that many companies have either revised their travel policies or continue to embrace virtual meetings as part of a post-pandemic shift in workplace practices. This analysis highlights that the perceptions of corporate ESG performance are shaped by data availability, that is, ESG transparency. By enabling the tracking of ESG indicators, our ML approach can contribute to the ability of the public to monitor corporate ESG performance.

Beyond emissions, progress across other environmental indicators is uneven, with some showing considerable changes, while others have stagnated (Fig. 5e–i). For example, the increasing share of renewable energy sources (Fig. 5f), accompanied by a decline in fossil-based energy (not reported here), indicates growing alignment with global decarbonization efforts and a gradual transition toward more sustainable energy sources. However, companies in the highest decile of energy intensity—measured as energy consumed per EUR of revenue—continue to rely predominantly on fossil fuels. Furthermore, energy (Fig. 5e) and water consumption (Fig. 5g) have declined by 37.7% and 38.1%, respectively, over the observation period. Reductions in total waste generated (Fig. 5h), and non-recycled waste (Fig. 5i) have been modest.

To account for the underlying trends in economic activity, we analyze inflation-adjusted median revenues across the sample period from the Worldscope database^43^. Overall, we find only a modest increase of 16.7% over the full sample period (see Supplementary Table S3), suggesting that macroeconomic growth alone is unlikely to fully explain the trends in ESG performance. By analyzing intensity ratios, we find that the companies in our sample seem to have made real adjustments in some areas (e.g., reducing scope 1 and 2 emission intensities), while, in other areas, the intensity ratios remain stagnant (e.g., energy consumption) or increase (e.g., indirect emissions) (see Supplementary Fig. S13).

To further explore heterogeneity across companies, we stratify our analysis of corporate ESG performance by different company characteristics, namely, market capitalization, ESG ratings, and ESG controversies scores, comparing companies in the top and bottom 10% of each characteristic (see Supplementary Fig. S22–S24). Here, scope 1 emissions are substantially lower among smaller companies, companies with lower ESG ratings, and those associated with fewer ESG controversies. We observe similar results for most of the other indicators.

The observed differences suggest that, in line with expectations, environmental performance is lower for larger companies and companies with more controversies around ESG practices. Notably, companies with higher ESG ratings also exhibit lower performance, and this pattern holds when analyzing intensities instead of absolute performance levels (as larger companies tend to have higher ESG ratings), highlighting the need to differentiate between sustainability transparency and performance (see Supplementary Fig. S14). There are two possible explanations for why companies with higher ratings exhibit lower environmental performance. First, ESG ratings as those by MSCI do not solely assess risks arising from negative impacts but also emphasize how these risks and opportunities are managed, which can favor larger firms with more advanced governance structures. Second, the MSCI rating is determined relative to peers within the same industry, meaning that companies operating in resource-intensive sectors (e.g., extractives & minerals processing) may still receive comparatively high ratings despite sizable absolute impacts if they outperform their sector counterparts^38^.

Finally, to account for changes in the number and composition of reporting companies over time, we further examine whether trends differ between early and late adopters of ESG-related reporting. Specifically, we conduct a separate analysis comparing firms that began ESG reporting early in the sample period to those that started later (see Supplementary Fig. S15). We find that overall trends in performance remain consistent across both groups, indicating that the main findings are not driven by sample composition. Restricting the sample to companies that report a given metric in all sample years yields closely similar trends (see Supplementary Fig. S18–S20).

### Social performance

**Fig. 7:**
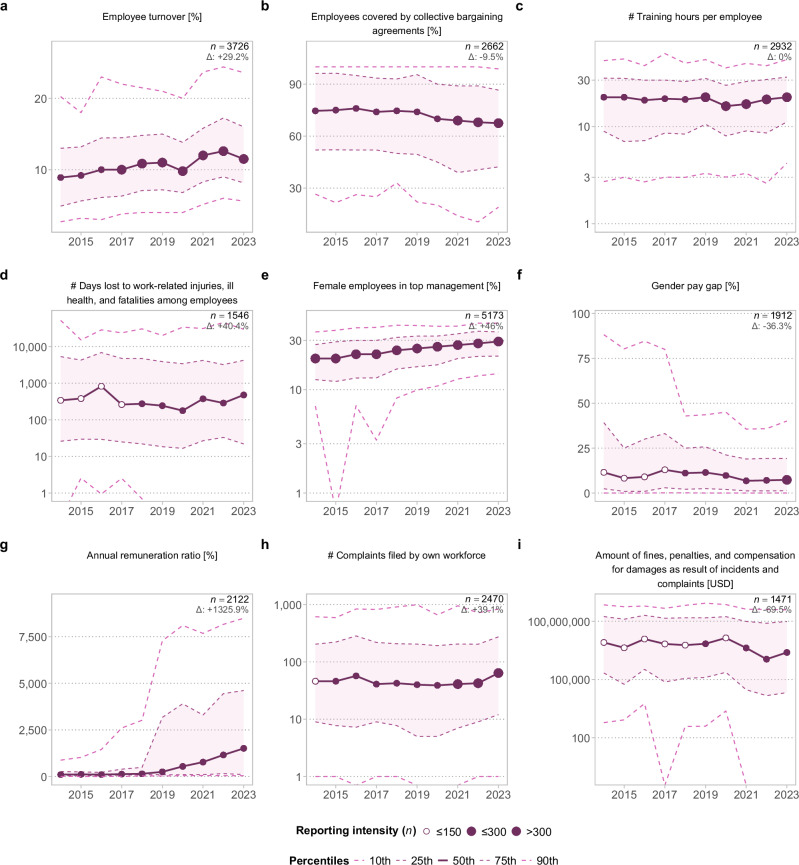
Social performance over time. Here, we analyze key social indicators between 2014 and 2023. Shown are (**a**) employee turnover, **b** the percentage of employees covered by collective bargaining agreements, **c** the number of training hours per employee, **d** the number of days lost to work-related injuries, ill health, and fatalities among employees, **e** the percentage of female employees in top management, **f** the gender pay gap, **g** the annual remuneration ratio (i.e., defined as the ratio of total annual compensation for top executives compared to the median employee), **h** the number of complaints filed by own workforce, and **i**, the number of fines, penalties, and compensation for damages as a result of incidents and complaints. For each indicator and year, we present the median value across all reporting companies (solid line, 50th percentile), the interquartile range (shaded band, bounded by the 25th and 75th percentiles, inner dashed lines), and the 10th and 90th percentiles (outer dashed lines) to capture the distribution and highlight variation between top and bottom-performing companies. For each panel, dot sizes indicate reporting intensity (i.e., the number of companies disclosing values for each indicator in a given year), *n* denotes the total number of company–year observations for the indicator, and Δ denotes the percentage change in indicator value between 2014 and 2023. Note that logarithmic axes are used to better visualize different orders of magnitude. For indicators recorded in percent, we use linear scales.

Performance across social indicators is mixed (Fig. 7). Notably, for instance, employee turnover has increased by 2.6 p.p. since 2014 (Fig. 7a), suggesting growing challenges in workforce retention. At the same time, the share of female employees in top management has increased steadily by 9.2 p.p. (Fig. 7e), which reflects ongoing efforts to promote gender equality in corporate leadership. The gender pay gap has narrowed by 4.7 p.p. since 2014 but has widened again by 0.5 p.p. since 2021 (Fig. 7f), suggesting potential setbacks in corporate efforts to promote equal pay. In 2023, the amount of fines, penalties, and compensation for damages as a result of incidents and complaints has decreased by 69.5% compared to 2014 levels. In contrast, the number of training hours per employee (Fig. 7c) remains stagnant over the observation period.

However, other indicators even exhibit decreases over time. The share of employees covered by collective bargaining agreements has decreased by 7.1 p.p., (Fig. 7b), indicating a weakening of employees’ bargaining power. Similarly, the annual remuneration ratio (i.e., the ratio of total annual compensation of the highest-paid individual to the median annual total remuneration for all employees, excluding the highest-paid individual) has increased by 1325.9% since 2014 (Fig. 7g), pointing to a widening gap between executive compensation relative to employee pay. The number of days lost to work-related injuries, ill health, and fatalities among employees has increased by 40.4% (Fig. 7d), mirroring an increase in the number of complaints filed by own workforce by 39.1% (Fig. 7h).

Consistent with our findings for environmental performance, companies that began ESG reporting earlier do not exhibit substantially different trends in social performance (see Supplementary Fig. S16), and trends among constant reporters are closely similar (see Supplementary Fig. S19). As expected, social indicators measured in absolute terms exhibit lower levels for smaller companies while relative metrics display comparable performance, with the exception of those pertaining to gender equality, where larger companies seem to perform better, possibly due to higher public exposure (see Supplementary Fig. S25). For ESG ratings and controversies, the results are mixed (see Supplementary Fig. S26–S27).

### Governance performance

**Fig. 8:**
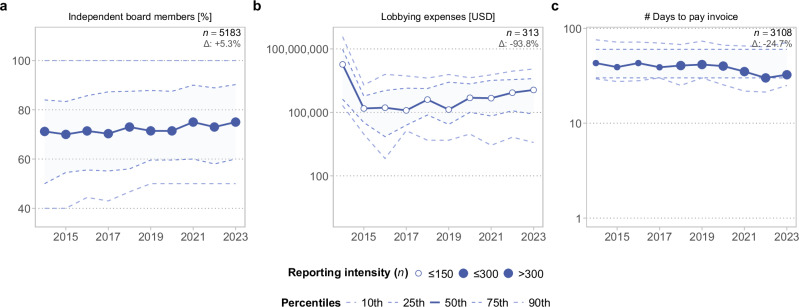
Governance performance over time. Here, we analyze key governance indicators between 2014 and 2023. Shown are (**a**) the percentage of independent board members, **b** lobbying expenses, and **c** the number of days to pay invoice. For each indicator and year, we present the median value across all reporting companies (solid line, 50th percentile), the interquartile range (shaded band, bounded by the 25th and 75th percentiles, inner dashed lines), and the 10th and 90th percentiles (outer dashed lines) to capture the distribution and highlight variation between top- and bottom-performing companies. For each panel, dot sizes indicate reporting intensity (i.e., the number of companies disclosing values for each indicator in a given year), *n* denotes the total number of company–year observations for the indicator, and Δ denotes the percentage change in indicator value between 2014 and 2023. Note that logarithmic axes are used to better visualize different orders of magnitude. For indicators recorded in percent, we use linear scales.

To understand governance practices (Fig. 8), we examine the share of independent board members, which remains consistently high, reaching 75.0% in 2023 (Fig. 8a), reflecting previous governance reforms aimed at strengthening board autonomy. In contrast, lobbying expenses have increased by 747.6% since 2019 (Fig. 8b). In the case of lobbying expenses, the earlier drop around 2014/15 could be attributed to limited or strategic disclosure by early adopters of ESG-related reporting (see Supplementary Fig. S17); trends among constant reporters are broadly consistent (see Supplementary Fig. S20). However, transparency on lobbying expenses remains generally low, resulting in fewer observations compared to other indicators shown here. Lobbying expenses are also higher for larger companies and companies with higher ratings, as well as more controversies around their ESG practices. For other governance indicators, the results are mixed, while larger companies tend to have a higher degree of independence within their board structure (see Supplementary Fig. S28–S30).

## Discussion

We extract and analyze granular, quantitative ESG indicators aligned with emerging sustainability reporting standards (that is, ESRS^35^). Although our findings point to an overall trend toward increased corporate ESG transparency, disclosure practices remain uneven, highlighting structural disparities shaped by topic- and industry-specific reporting priorities. Regarding ESG performance, our analysis shows heterogeneous trends across key indicators, including large increases in some measures (e.g., remuneration ratios). We note, however, that some of these observed changes coincide with expanded disclosure, highlighting how evolving transparency can affect the interpretation of reported ESG performance.

Our analysis is based on a comprehensive dataset on corporate ESG-related transparency and performance, which comprises 501 quantitative ESG indicators across nine ESRS topics. The main purpose of our analysis is to show the scientific value of the dataset for hypothesis generation and subsequent theory building, by providing descriptive insights that can subsequently be examined using causal inference methods. To construct our dataset, we leverage large language models as state-of-the-art techniques from machine learning. Whereas such machine learning models have been used in sustainability research^17,19,21,22,44–50^, for example, to analyze sustainability narratives^23–26^, we fill evidence gaps regarding transparency and performance by analyzing quantitative ESG indicators. We also address key limitations of existing ESG analyses that are based on manual data collection and thus restricted to a few, selected indicators (e.g., carbon emissions, energy use, or water withdrawal^12–16^) or limited to certain countries^18,19^, sectors^4,17^, or short time periods^20–22^. In contrast, our framework enables tracking heterogeneity in trends across broad samples, e.g., to identify cross-industry differences or regional disparities. Furthermore, our open science approach democratizes data access.

Our analysis of corporate transparency reveals persistent gaps in ESG disclosures, which suggests that the transparency of current reporting practices is unevenly distributed across companies, sectors, and topics^51^. In interpreting these patterns, we accordingly refrain from making causal claims but highlight several cases where the findings are consistent with theoretical expectations.

The overall increase in indicator transparency is consistent with anticipated growth in regulatory constraints, investor demand, competitive pressure from peers, and expanding rating agency coverage^12,52–55^. While better extraction performance in more recent reports could, in principle, contribute to this trend, we observe similar patterns when analyzing a subset of high-quality reports, suggesting that the trend is not an artifact of our extraction pipeline.

The observation that the transparency gap between higher-rated and lower-rated companies becomes narrower over time is consistent with a convergence dynamic: as sustainability reporting transitioned from a voluntary differentiator to a common and eventually mandatory practice, laggards increasingly adopted disclosure norms established by early leaders^54,55^. A regression analysis that interacts rating group dummies with a linear time trend confirms this pattern, indicating that lower-rated companies display relatively stronger increases in transparency over time, though the differential trends attenuate once company-level controls and fixed effects are included (Supplementary Table S2). Notably, ESG ratings themselves increased over the sample period, and, because ratings may, at least partially, reflect transparency, including a one-year lag mitigates—but does not fully eliminate—the concern that the association between ratings and transparency is partly mechanical^54,56,57^.

Higher transparency scores among larger companies are consistent with greater resource availability, stronger regulatory exposure, more intense stakeholder scrutiny, and greater exposure to a diverse set of ESG topics^51,58,59^. An alternative explanation is that our extraction pipeline may perform better on reports from larger companies if these reports have higher document text quality; however, we find similar associations when analyzing a subset of reports with high document text quality (see Supplementary Fig. S7–S12). Notably, the size-transparency relationship becomes statistically insignificant once we control for ESG ratings and controversy exposure (see Table 1, Panel A), and remains insignificant across all subsequent specifications including sector and company fixed effects. This suggests that the univariate association between size and transparency largely reflects other company characteristics correlated with size, such as rating coverage and controversy exposure, rather than size per se.

We further analyze the association between ESG controversy exposure, measured through the Refinitiv ESG controversies score^60^, and transparency (see Table 1, Panel C). We find that companies with lower controversy exposure have significantly lower transparency scores, while the difference for companies with higher controversy exposure is small and not statistically significant. This pattern admits several interpretations. Companies with less controversy exposure may face weaker external pressure to disclose comprehensively, or, conversely, companies that disclose more may expose themselves to greater scrutiny, creating a mechanical link between transparency and measured controversy exposure^58,59,61^. Once we control for market capitalization and ESG ratings (see Table 1, Panel C, Model 2–6), the association becomes statistically insignificant, suggesting that the univariate association between controversy exposure and transparency largely captures other company characteristics correlated with controversy exposure, consistent with recent research on the company characteristics that shape ESG transparency, such as size, being third-party rated, analyst coverage, and institutional holdings^12,51^.

In addition, the observed disparities in transparency across sectors and topics highlight the need for regulatory frameworks that mandate the standardized disclosure of material ESG indicators to ensure consistency, comprehensiveness, and comparability in corporate ESG reporting^55,62^. Cross-sectional variation in topic-level transparency—i.e., highest for own workforce and governance, lowest for pollution and biodiversity—likely reflects the maturity of established reporting frameworks and the degree of stakeholder attention that different topics receive^63,64^. Pollution and biodiversity disclosures may lag because these topics lack standardized metrics and are material to fewer companies^65^. Alternatively, our pipeline may perform better on topics with well-codified terminology than on less standardized areas, or these topics may be genuinely non-material for many companies in our sample.

Our assessment of environmental performance reveals several trends in direct environmental indicators (e.g., scope 1 GHG emissions and renewable energy adoption). The decline in median scope 1 and scope 2 emissions is consistent with a reduction in direct emissions (e.g., through operational efficiencies) and a shift toward lower-carbon energy sources for direct operations, which is corroborated by the concurrent increase in renewable energy shares we document. However, these reductions may also partly reflect divestiture of high-emitting assets, offshoring of production, or reclassification of emissions across scopes rather than genuine abatement. We examine inflation-adjusted median revenues across the sample period and find only a modest increase (16.7%), and do not document a downturn in overall economic activity that might independently explain the emissions decline. We also present company-level intensity measures in support of our findings (see Supplementary Fig. S13).

Moreover, the concurrent increase in scope 3 GHG emissions shows the challenges companies face in managing emissions beyond their immediate operations—particularly across complex supply chains^13^. In light of widespread net-zero pledges^7,8^ and the legally binding goal set by the EU to reach climate neutrality by 2050^66^, the current trajectory of emissions suggests that many companies remain considerably off-track. The new EU Corporate Sustainability Due Diligence Directive (CSDDD)^40^ may alter this trajectory by requiring that companies identify and address environmental risks along their value chains, thus strengthening accountability for scope 3 GHG emissions.

Notably, trends in reported scope 3 GHG emissions may reflect not only changes in underlying activities but also increased transparency, including greater granularity, broader scope of coverage, and improved measurement practices. Furthermore, because scope 3 emissions encompass indirect emissions across value chains and often involve overlapping reporting boundaries, we emphasize the need for policymakers to interpret scope 3 emission data cautiously to avoid double-counting and therefore misinformed conclusions or misguided policy responses. Additionally, our results show that major emitters continue to rely heavily on fossil energy sources, with limited adoption of renewables, implying that, despite the technical feasibility of decarbonizing industrial processes, current market incentives and regulatory pressures may be insufficient to drive large-scale transition in carbon-intensive sectors. Further, several other environmental dimensions—such as water or waste management—exhibit less progress in terms of performance, but often also regarding transparency (e.g., few companies disclose biodiversity-related indicators). These findings highlight the importance of regulatory incentives to strengthen environmental performance holistically across entire value chains.

Similarly, uneven performance across social indicators reflects continued efforts to improve gender diversity in leadership, but limited progress toward greater pay and income equality. The steady increase in female representation in top management is consistent with growing regulatory pressure (e.g., such as Directive (EU) 2022/2381 on gender balance on corporate boards) and evolving stakeholder expectations, though it may also partly reflect expanded definitions of top management or selective reporting of favorable metrics. The gender pay gap narrowed overall but widened slightly after 2021, which could reflect pandemic-related workforce disruptions and return-to-office policies that differentially affected women. The large increase in remuneration ratios suggests that executive compensation outpaced median wages, potentially driven by equity-based pay structures and market performance during the sample period. Country-level regulatory differences, industry trends, and macroeconomic conditions may further shape these drifts.

Governance indicators show an increase in board independence and a decrease in the number of days to pay invoices, echoing Directive (EU) 2017/828 on shareholder voting rights regarding executive pay, or Directive 2011/7/EU on harmonized payment terms to curb abusive payment practices towards small-medium enterprises. In contrast, increasing lobbying expenditures since 2019 point to the need for stronger disclosure and oversight. Overall, these patterns highlight the limits of voluntary corporate action and underscore the role of policy in addressing persistent transparency gaps across ESG dimensions.

Our ML framework has several strengths. First, it generates a highly granular dataset that covers 501 different ESG indicators across nine ESRS topics. Consequently, our dataset is considerably more comprehensive than other available datasets, including those from commercial providers (e.g., Refinitiv^60^). Second, our framework is scalable, which enables automated ESG analysis across time, industries, and countries. The scalability also enables regular updates of our dataset, as well as extending the dataset to emerging ESG frameworks and other geographical regions. Third, we open-source both our dataset and framework to democratize access to ESG data. So far, ESG data is often buried in lengthy reports or needs to be purchased at high cost from commercial providers that invest in the costly process of manual extraction, or that use their own, often diverging, definitions of ESG indicators. By providing open-source access, we make ESG data freely accessible to stakeholders including policy-makers, investors, and societal actors.

Our work is subject to the following limitations. First, the observed patterns are consistent with multiple causes, yet we refrain from such causal claims and interpret the results as descriptive, opening avenues for future research using our dataset. Second, as we apply the ESRS framework retrospectively to reports published before ESRS became mandatory, documented data gaps may partly reflect differences in the reporting frameworks under which companies report. Transparency scores should therefore be interpreted as alignment with current ESRS requirements. However, ESRS are largely based on previously established frameworks, especially GRI, and thus overlap substantially in underlying indicators, making our findings relevant beyond ESRS-specific requirements. Third, sustainability reports are self-reported and, during our sample period, subject to limited assurance. While our framework can detect what is disclosed, it cannot identify strategic non-disclosure. Nevertheless, such selective disclosures should be mitigated through future regulations, including ESRS, with mandatory audits of such data being rolled out. Importantly, outright falsification of financial or ESRS reports constitutes a legal offence under existing capital market regulations, which may deter systematic data manipulation. Moreover, non-retrieval of an indicator can reflect either non-disclosure or imperfect extraction (e.g., due to ambiguous information in the report); hence, absence of an extracted value does not necessarily represent the absence of reporting. Fourth, our sample comprises the 600 largest listed European companies, placing it at the upper tail of company size, visibility, and institutional coverage. Our findings may not generalize to smaller or private companies, or to non-European markets where regulatory pressure and stakeholder expectations differ substantially. Nevertheless, our ML framework is scalable, enabling future research to extend the analysis to other regions and company populations. Fifth, the accuracy of the RAG pipeline depends on the structure, clarity, completeness, and consistency of the corporate reports analyzed. Variations in data quality—such as missing or ambiguous information—can affect extraction accuracy, and LLM-based extraction may be prone to errors arising from hallucinations and the opaque reasoning processes inherent in LLMs. We thus validated extracted values against two external datasets (see Supplementary Fig. S1) to confirm the reliability of our framework.

In sum, targeted transparency through high-quality ESG disclosures empowers policy-makers, financial stakeholders, and societal actors to hold companies accountable for their ESG efforts—in turn, advancing global sustainability goals. Our framework allows these stakeholders to systematically track corporate ESG-related transparency and performance at scale. It thus provides a basis for evaluating the effectiveness of emerging regulatory initiatives on corporate ESG reporting and progress toward global sustainability goals.

## Methods

### Data

Our sample consists of all companies listed in the STOXX Europe 600 stock index in 2023. The index constituents come from a broad range of countries and industries, covering nearly 90% of the investable market in Europe^67^. As a result, our dataset includes companies from 16 European countries and 11 industry sectors. The sectors are classified according to the Sustainable Industry Classification System (SICS), which was developed by the Sustainability Accounting Standards Board (SASB) to group companies into sectors with comparable exposure to sustainability-related risks and opportunities^68^. Company fundamentals, such as market capitalization and sales, are sourced from the Worldscope database, a service by the London Stock Exchange Group^43^. An overview of the companies included in the dataset is provided in Supplementary Table S1. Coverage across industries and countries over time is shown in Supplementary Fig. S5.

For all companies, we collect both annual reports and sustainability reports published as PDF files (overall 9,173 documents) between 2014 and 2023. The publicly available reports are sourced from intermediaries (i.e., https://annualreports.comand https://sustainabilityreports.com) as well as directly from companies’ websites. The distribution of annual and sustainability reports over time is shown in Supplementary Fig. S3. Overall, the PDF files have an average length of 183 pages, which amounts to a total of 1,678,551 pages across the dataset. The word count is 88,965 on average per PDF. In our ML framework, we later concatenate both the annual and sustainability reports to form a panel dataset with company–year observations.

In these corporate reports, we then track 501 quantitative ESG indicators defined by European Sustainability Reporting Standards (ESRS)^35^. ESRS are described in the Supplementary Information S1. A complete list of the ESG indicators including metadata is provided in a supplementary CSV file.

### ML framework

We developed an ML framework to extract quantitative ESG indicators from corporate reports based on retrieval-augmented generation (RAG)^69^. Our framework consists of five steps (see Supplementary Fig. S4): In step 1, we preprocess the corporate report PDF files and divide the text into meaningful chunks (*preprocessing*). In step 2, we embed the text chunks and store the vector representations in a vector database (*indexing*). In step 3, for each ESG indicator description, the top-*k* most similar report chunks are retrieved and re-ranked (*retrieval*). In step 4, each ESG indicator description and the corresponding report chunks are passed in a tailored prompt to a pre-trained LLM, which outputs the extracted ESG indicator (*generation*). Finally, in step 5, the model output is postprocessed by standardizing values and units (*postprocessing*). Key methodological limitations are discussed in the Supplementary Information S4.

Step 1: preprocessing. First, we used PyMuPDF (v1.24.9)^70^ to parse the corporate report PDF files. The extracted text was cleaned by normalizing white spaces and resolving character encoding mismatches. For each company and reporting year, the corresponding annual and sustainability reports were concatenated into a single document, denoted as *d*. Each document *d* was then partitioned into a sequence of semantically coherent chunks ${{{\mathcal{C}}}}_{d}=\{{c}_{1,d},{c}_{2,d},\ldots,{c}_{M,d}\}$, where *M* is the total number of chunks for document *d*, determined by the document length and the fixed chunk size and overlap parameters. This ensures efficient retrieval and compatibility with the context length constraints of the LLM in step 4. Specifically, we applied recursive character text splitting^71^, which is designed to preserve meaningful contextual boundaries, to partition each document *d* into its corresponding set of chunks ${{{\mathcal{C}}}}_{d}$. Each chunk *c*_*i*,*d*_ was restricted to a maximum length of 400 characters, with an overlap of 100 characters between consecutive chunks, to ensure the granularity for precise similarity matching while minimizing the risk of sentence fragmentation^72^.

Step 2: indexing. We transformed each text chunk *c*_*i*,*d*_ into a dense vector representation using the pre-trained embedding model all-MiniLM-L12-v2^73^, implemented via sentence-transformers (v3.1.0). The model all-MiniLM-L12-v2 is an efficient yet semantically capable transformer-based model, which makes it well-suited for the large-scale retrieval task in this work^74^. The model maps each chunk *c*_*i*,*d*_ to a point in a 384-dimensional vector space, where semantically similar texts are positioned closer together, while dissimilar ones are more distant. The model all-MiniLM-L12-v2 is based on the MiniLM model family, which achieves state-of-the-art performance while improving computational efficiency^75^. To improve its ability to encode semantically meaningful representations, all-MiniLM-L12-v2 was fine-tuned on a corpus of more than one billion sentence pairs using a contrastive learning objective. Given a sentence from a training pair, the all-MiniLM-L12-v2 model was trained to identify the associated sentence from a set of randomly sampled alternatives. Formally, cosine similarity is computed for all sentence pairs in a batch, and a cross-entropy loss is applied to maximize similarity for correct pairs while minimizing it for incorrect pairs. This ensures that semantically related chunks *c*_*i*,*d*_ are embedded closer together in the vector space while unrelated ones are pushed apart^73^.

Step 3: retrieval. The resulting vector representations for all chunks *c*_*i*,*d*_ were stored in a FAISS vector database, implemented using faiss-cpu (v1.8.0.post1), which enables scalable and low-latency retrieval^76,77^. Next, for each document *d* and each ESG indicator description, denoted as query *q*, we retrieved the top-30 most relevant report chunks, forming the set ${{{\mathcal{C}}}}_{q,d}^{30}$. The indicator description *q* consists of the relevant indicator subtopic and its description according to ESRS (e.g., gross scope 3 greenhouse gas emissions: category 1.1 cloud computing and data center services or percentage of employees at top management level: female). We used a state-of-the-art hybrid search method that integrates cosine similarity search and keyword-based search with equal weighting^72^, implemented using faiss-cpu (v1.8.0.post1) and rank-bm25 (v0.2.2), which enables contextual semantic understanding with precise term-based retrieval.

The top-30 most relevant chunks ${{{\mathcal{C}}}}_{q,d}^{30}$ were re-ranked using the cross-encoder model Llama-Rank-V1^78^, a fine-tuned variant of Llama3-8B-Instruct^79^ trained on relevance-annotated data. Unlike bi-encoder models such as all-MiniLM-L12-v2, which encode queries and documents independently, cross-encoders jointly process query-chunk pairs (*q*, *c*_*i*,*d*_) to capture fine-grained semantic relationships. Due to the high computational cost of cross-encoders, we employed a two-stage strategy: the efficient bi-encoder all-MiniLM-L12-v2 retrieved a candidate top-30 set, which was then re-ranked by Llama-Rank-V1. This balances retrieval efficiency with ranking precision. Specifically, Llama-Rank-V1 computed a numeric relevance score for each query-chunk pair (*q*, *c*_*i*,*d*_), where ${c}_{i,d}\in {{{\mathcal{C}}}}_{q,d}^{30}$. The top-10 re-ranked chunks ${{{\mathcal{C}}}}_{q,d}^{10}$ were expanded by concatenating each ${c}_{i,d}\in {{{\mathcal{C}}}}_{q,d}^{10}$ with its preceding chunk *c*_*i*−1,*d*_ and subsequent chunk *c*_*i*+1,*d*_, respectively. This then resulted in a set of extended chunks ${{{\mathcal{C}}}}_{q,d}^{10*}=\{({c}_{i-1,d}\parallel {c}_{i,d}\parallel {c}_{i+1,d})| {c}_{i,d}\in {{{\mathcal{C}}}}_{q,d}^{10}\}$. This approach is known to mitigate the risk of missing important details that may span across chunk boundaries, such as large cohesive tables^72^. As a result, step 3 returned the most contextually relevant information from the corpus.

Step 4: generation. For each document *d* and query *q*, the corresponding retrieved report chunks ${{{\mathcal{C}}}}_{q,d}^{10*}$ were included in a tailored prompt and passed to a pre-trained LLM. The prompt is based on best practices in LLM prompt design^80–82^ and instructs the model to refrain from hallucinating if the requested ESG indicator is absent from the corporate report. The exact prompt is stated in Supplementary Information S3. Specifically, the model is asked to return the target value and unit of the requested ESG indicator described by *q*, if reported, in JSON format. JSON ensures structured, machine-readable output that facilitates downstream processing. We selected Llama-3.1-70B-Instruct^83^ as inference model, which offers state-of-the-art performance in natural language understanding and generation tasks without the excessive computational cost of larger models. Llama-3.1-70B-Instruct is an externally developed, instruction-tuned large language model with publicly released weights. We did not update model parameters on our corpus, and all outputs were generated via prompting conditioned on the retrieved report chunks. Furthermore, Llama-3.1-70B-Instruct was fine-tuned for instruction-following tasks^79^, which makes it better suited for structured information extraction than standard generative models. We intentionally chose Llama-3.1-70B-Instruct over alternative, proprietary models (e.g., GPT-4) for two reasons: first, it gives state-of-the-art performance at the time of writing, and, second, it is open-source, meaning that end-users can easily adopt our framework^84^. We accessed Llama-3.1-70B-Instruct via the Together AI API^85^ using the together Python package (v1.3.3). Informed by best practices for LLM-based research^80^, we set the temperature to 0, which leads the model to consistently select the token with the highest probability. This ensures deterministic output and, thus, promotes reproducibility. Reporting follows best practice^86^.

Step 5: postprocessing. Finally, we perform the following postprocessing steps. Recall that the model output for each document *d* and query *q* contains the ESG indicator value and corresponding unit. The latter was standardized to adhere to the units specified by the European Financial Reporting Advisory Group (EFRAG)^35^ as follows. We used regular expressions to unify the output strings, and the Pint library (v0.24.4)^87^ to convert the unified strings to standard units. The currencies were converted to USD according to the annual exchange rates provided by the Federal Reserve^88^. Thus, we enable robust downstream analysis and ensure a structured dataset for future research. The detailed regular expressions for the standardization process are in our source codes. Eventually, the final dataset is stored in a CSV file.

The pipeline was implemented in Python (v3.12.10). Statistical analyses, tables and figures were generated using custom Python and R (v4.4.0) scripts. Complete package versions and installation requirements are provided in the public repository listed in the Code Availability statement and in the Reporting Summary.

### Validation

We validated our ML framework using two datasets: (1) a proprietary dataset of ${N}_{{{\rm{perf}}}}=212,713$ values across 38 ESG indicators, sourced from LSEG Refinitiv, a provider of financial and ESG data^60^; and (2) a human-annotated subset of ${N}_{{{\rm{perf}}}}=210$ values across 21 ESG indicators (the raw data is made publicly available in our code base). The proprietary dataset was compiled by Refinitiv from publicly available sources, including company disclosures, stock exchange filings, and third-party information such as news websites. However, not all data points are directly cross-verified by the companies themselves. The human-annotated dataset was annotated independently by two PhD-level researchers (K.F. and L.K.) familiar with ESRS reporting, each of whom annotated half of the dataset, such that the entire dataset was covered by expert annotation. To ensure the reliability of the annotations, another annotator—a graduate-level university student with prior experience in corporate ESG analysis—independently annotated the full subset. Then, the inter-rater reliability was assessed. Specifically, we computed the intraclass correlation coefficient (ICC) for each subset using a two-way mixed-effects model with absolute agreement and single measures^89^. We found consistently high ICC values across both annotator combinations (subset 1: ICC = 0.978, 95% CI [0.968, 0.985]; subset 2: ICC  = 0.998, 95% CI [0.997, 0.998]), which indicates excellent agreement and thus strong support for the reliability of the manual annotations.

However, both datasets come with inherent limitations. (1) The proprietary dataset covers only 38 ESG indicators—which is fewer than the 501 indicators defined by ESRS. (2) The human-annotated dataset is constrained by the manual nature of the annotation process, which limits scalability and thus size. Manual annotation is particularly time-intensive due to the length and complexity of sustainability reports, which often span hundreds of pages. As a result, each value took us ~15–20 minutes to annotate, which corresponds to roughly 105–140 h (or around three weeks under regular working hours) of full-time labeling. We initially considered delegating the annotation task to trained university students; however, this approach proved unsuccessful due to insufficient annotation quality. Despite thorough training, student annotators frequently introduced mismatches in definitions or units. Due to these issues, we reverted to relying on experienced researchers for annotation. Hence, both validation datasets remain substantially smaller in scope—both in terms of ESG indicators and number of companies—than our machine-learning-based dataset.

We then proceeded as follows. (1) For both datasets, we compared our extracted ESG indicators against the corresponding values from the validation datasets (see Supplementary Fig. S1 for both Refinitiv and the manually annotated validation dataset). In both cases, the correspondence is strong: for the proprietary dataset, the estimated slope from the OLS regression is *β* = 0.885 (*p* < 0.001), with an adjusted *R*^2^ of 0.91, and, for the manually annotated dataset, the estimated slope from the OLS regression is also *β* = 0.885 (*p* < 0.001), with an adjusted *R*^2^ of 0.93. Hence, we find a strong agreement between our ML framework and the benchmark datasets. (2) To further validate the performance of our framework, we evaluated standardized mean absolute error (sMAE) and standardized root mean squared error (sRMSE). These metrics normalize absolute and squared errors by the interquartile range (IQR) of the human-annotated values for each indicator, which enables comparison across indicators with different scales for better interpretability while providing robustness against outliers. sMAE measures deviations between predictions and human-annotated reference values, while sRMSE additionally penalizes large errors, thus providing a more conservative assessment. Macro-averaged across indicators, the framework achieves an sMAE of 0.21 (95% CI [0.08,  0.37]) and sRMSE of 0.39 (95% CI [0.15,  0.54]) (Supplementary Table S5), demonstrating robust alignment with human-annotated values. Indicators with undefined denominators (IQR = 0) and one indicator with only three observations, where a single prediction error produced unstable standardized metrics, were excluded from the macro-averages to maintain reliability of the aggregated estimates. Confidence intervals for the aggregated metrics were estimated using a nonparametric percentile bootstrap with 2,000 resamples. (3) To assess potential heterogeneity across ESG indicators, we computed the agreement for each ML-extracted ESG indicator with the proprietary dataset from Refinitiv. We consistently find positive and statistically significant correlations for the majority of ESG indicators (Supplementary Fig. S2), with particularly high agreement for indicators related to greenhouse gas emissions, suggesting that our extraction approach is not biased toward specific topics but overall robust. We focused on the Refinitiv dataset in the heterogeneity analysis because the human-annotated validation set is too small (all *n*≤10) to yield statistically reliable estimates, whereas the proprietary dataset typically contains *n* ≫ 100 values per indicator, which thus allows for such a heterogeneity analysis.

### Reporting summary

Further information on research design is available in the Nature Portfolio Reporting Summary linked to this article.

## Supplementary information

Supplementary Information

Reporting Summary

Transparent Peer Review file

## Data Availability

The complete dataset of extracted ESG indicators generated in this study has been deposited in the Open Science Framework (OSF) repository under accession code q2jpv (https://osf.io/q2jpv/). The corporate annual and sustainability reports used in this study have been deposited in Harvard Dataverse under accession code DVN/84HKPS (10.7910/DVN/84HKPS). The remaining third-party datasets used for validation and additional analyses are available under restricted access because they are proprietary and require a subscription or license; access can be obtained from the respective data providers. Specifically, the LSEG Refinitiv ESG data used for validation are available from LSEG; company fundamentals data are available from LSEG Worldscope Fundamentals (https://www.lseg.com/en/data-analytics/financial-data/company-data/fundamentals-data/worldscope-fundamentals); ESG controversies scores are available from LSEG ESG Scores (https://www.lseg.com/en/data-analytics/sustainable-finance/esg-scores); and MSCI ESG ratings are available from MSCI.

## Code availability

The code used to reproduce the analyses, tables, and figures reported in this study is available on GitHub at https://github.com/forsterkerstin/corporate-sustainability-trackerand has been archived on Zenodo^90^. Llama-3.1-70B-Instruct is available at https://huggingface.co/meta-llama/Llama-3.1-70B-Instruct.
