# Supplementary material for: Assessing corporate sustainability with large language models: evidence from Europe
Source: Nat Commun. 2026 Jul 7;17:5940. doi: 10.1038/s41467-026-75160-z (PMC13341771; doi:10.1038/s41467-026-75160-z)
Supplement: Supplementary file 1 — Supplementary Information [file 41467_2026_75160_MOESM1_ESM.pdf]

Supplements

Supplementary Figures

S1 Comparison of ML-based and validation dataset values 3
S2 Correlation of ML-based and proprietary validation dataset values 4
S3 Corporate annual and sustainability reports per year 5
S4 Overview of our machine learning framework 6
S5 Scope and annual coverage 7
S6 Pipeline coverage analysis 8
S7 Transparency trends for high-quality documents and full sample. 9
S8 Transparency across ESRS topics and industries for high-quality documents. 10
S9 Heterogeneity in ESG-related transparency across company characteristics for high-quality documents. 11
S10 Environmental performance trends for high-quality documents and full sample. . . 12
S11 Social performance trends for high-quality documents and full sample. 13
S12 Governance performance trends for high-quality documents and full sample. 14
S13 Intensity trends for selected environmental indicators for early adopters and full sample 15
S14 Intensity trends for selected environmental indicators by rating 16
S15 Environmental indicators for early adopters and full sample 17
S16 Social indicators for early adopters and full sample 18
S17 Governance indicators for early adopters and full sample 19
S18 Environmental indicators for constant reporters and full sample 20
S19 Social indicators for constant reporters and full sample 21

S20 Governance indicators for constant reporters and full sample 22
S21 # Observations per year by scope 3 greenhouse gas emissions category 23
S22 Differences in environmental indicators by market capitalization 24
S23 Differences in environmental indicators by ESG rating 25
S24 Differences in environmental indicators by ESG controversies score 26
S25 Differences in social indicators by market capitalization 27
S26 Differences in social indicators by ESG rating 28
S27 Differences in social indicators by ESG controversies score 29
S28 Differences in governance indicators by market capitalization 30
S29 Differences in governance indicators by ESG rating 31
S30 Differences in governance indicators by ESG controversies score 32
S31 Prompt design 74

Supplementary Tables

S1 List of companies by name and country, grouped by SICS sector 33
S2 Transparency trends by rating group 43
S3 Inflation-adjusted median revenues in 2023 EUR 44
S4 Regression analysis for scope 3 emissions 45
S5 Per-indicator standardized mean absolute error (sMAE) and standardized root mean squared error (sRMSE). 46
S6 Per-indicator disclosure detection and standardization coverage. 47

Supplementary Information 69

Supplementary Figures

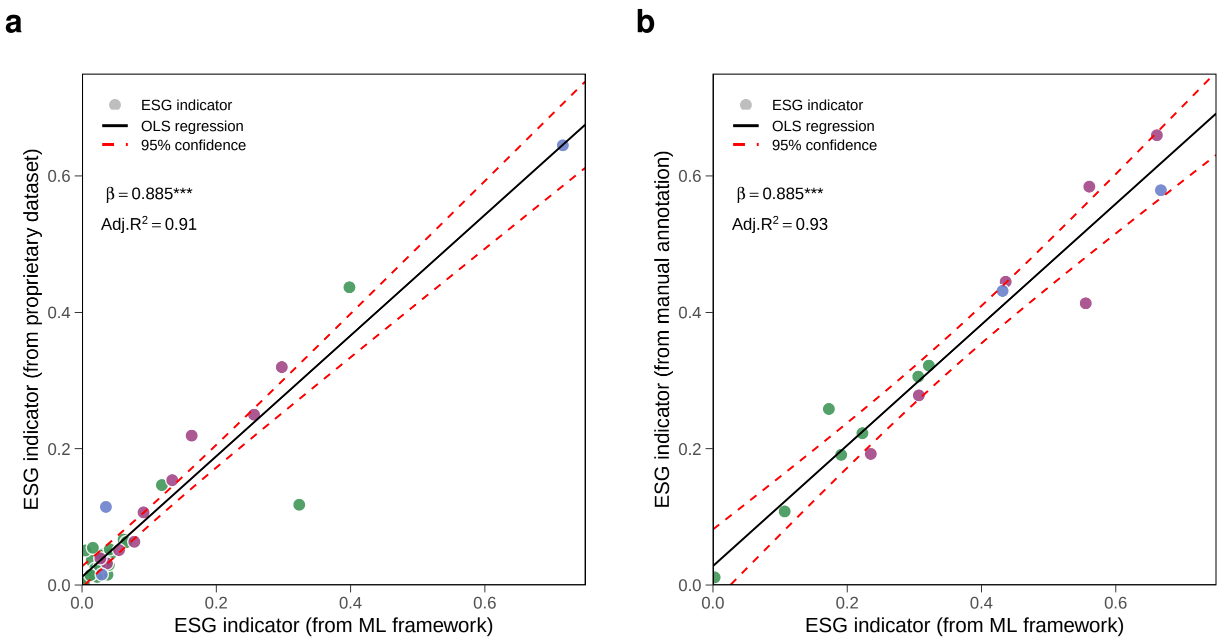

Fig. S1. Comparison of our ML-based extraction against (a) Refinitiv data and (b) manually annotated data. Here, we correlate the extracted values from our ML framework against the values from the validation datasets. The x-axis shows the average value of the ESG indicator extracted by the ML framework, while the y-axis shows the average value of the ESG indicator as reported in **a**, Refinitiv data (proprietary; limited number of ESG indicators) and **b**, the manually annotated data (limited coverage across company–years). For direct comparability across ESG indicators, the values are normalized to the range from 0 to 1. To reduce the influence of extreme outliers, we excluded the top 1% of values within each indicator prior to normalization. Further, we report only indicators with more than three observations in both the ML-generated and validation datasets. Overall, the values of the ESG indicators in both our ML-generated dataset and the validation datasets show strong agreement, but ours offers a broader coverage in terms of ESG indicators and company years. The only outlier (green dot, bottom right) is the indicator referring to Total greenhouse gas emissions reduction (in %) (reported by $n = 40$ companies). To understand the source of the disparities, we manually reviewed corporate reports and found inconsistencies in how companies present this indicator. Companies report the indicator in different ways: some disclose annualized emission reductions, others report targets for a specific year, while others describe reductions already achieved. Hence, the inconsistency between both datasets is neither a problem of our method (nor of the Refinitiv data), but rather a problem of interpretation. This variation highlights that the ESG indicator currently lacks a standardized reporting approach. Consequently, our analysis shows that the reporting standards in ESRS should define this dimension more precisely to improve comparability across disclosures.

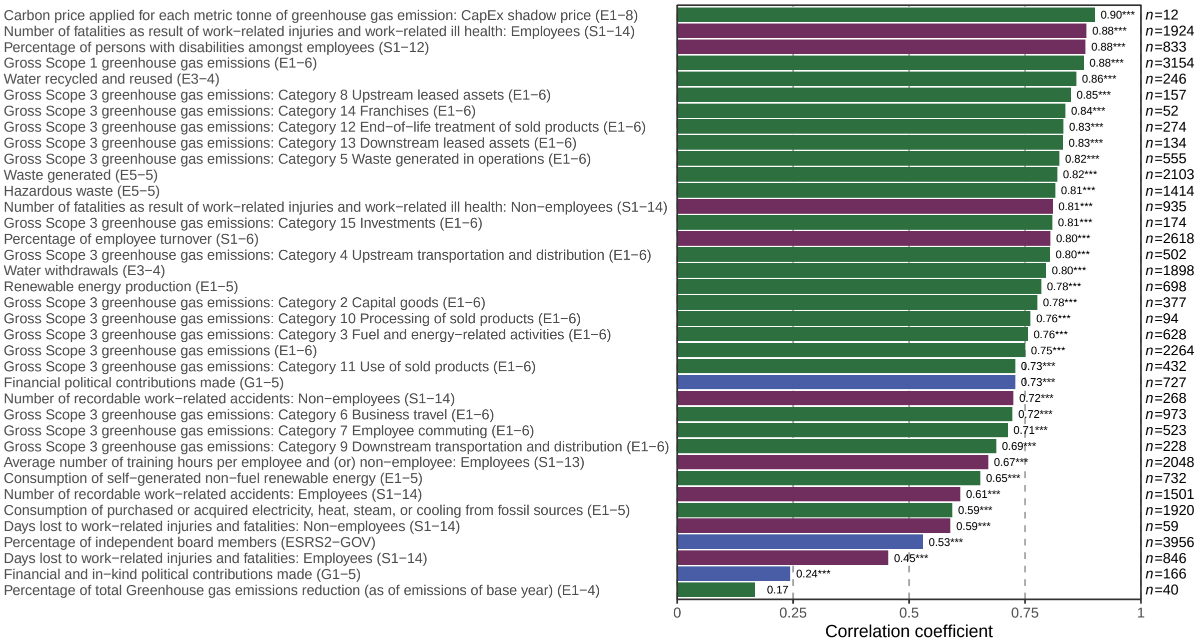

Fig. S2. Comparison between the ML-based and the proprietary validation datasets across different ESG indicators. Here, we assess potential heterogeneity in the extracted values across different ESG indicators. We thus report the Spearman correlation coefficients between the values generated from our ML framework and the proprietary validation dataset, separately for each ESG indicator. The annotation on the right-hand side (n) is the number of values used for the comparison (i.e., the number of values where corresponding values are recorded in both our dataset and the validation dataset). Significance levels based on Spearman rank-order correlation test: * $p < 0.05$, ** $p < 0.01$, *** $p < 0.001$. For the human-annotated validation dataset, we do not report correlation coefficients, as the number of observations per ESG indicator is too small (all $n \leq 10$) to yield meaningful and statistically reliable estimates. Instead, to ensure full transparency and enable qualitative assessment, we provide the complete annotated dataset as a supplementary CSV file.

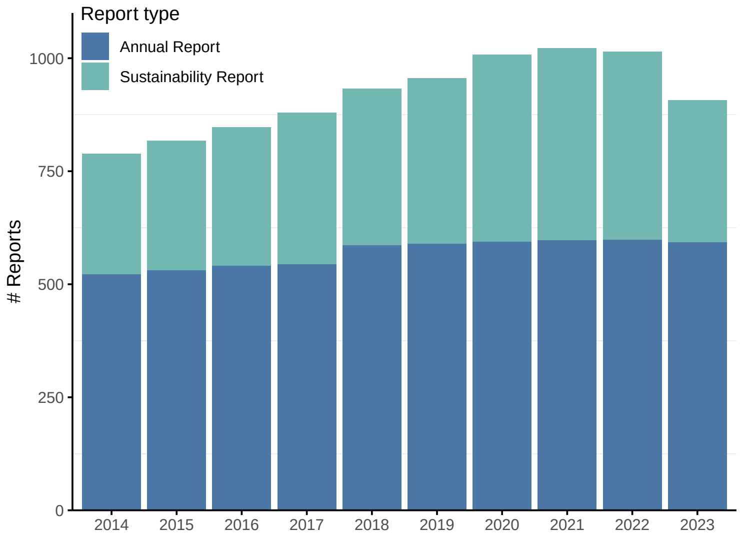

Fig. S3. Corporate annual and sustainability reports per year.

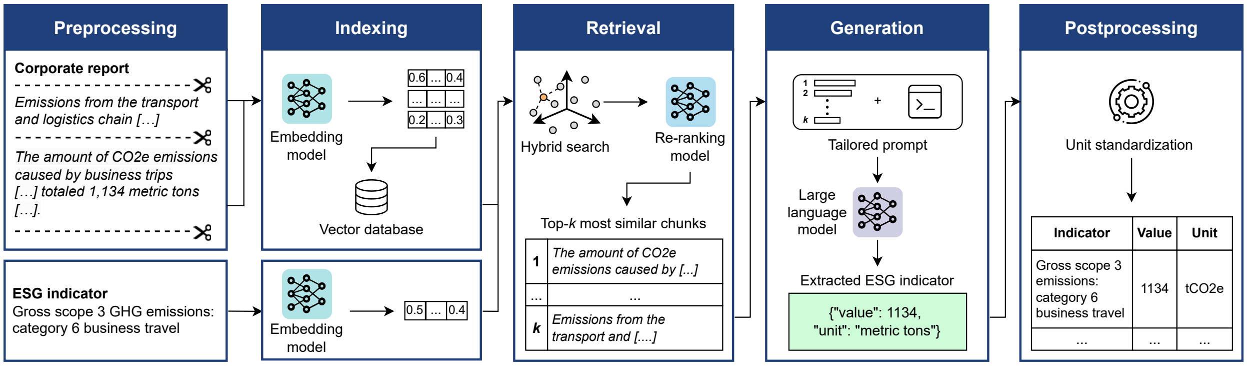

Fig. S4. Overview of our machine learning framework to extract ESG indicators from corporate annual and sustainability reports. Icons were obtained from Flaticon (https://www.flaticon.com/): neutral network by Vectors Tank, database by Smashicons, window by LAFS, technical support by Freepik.

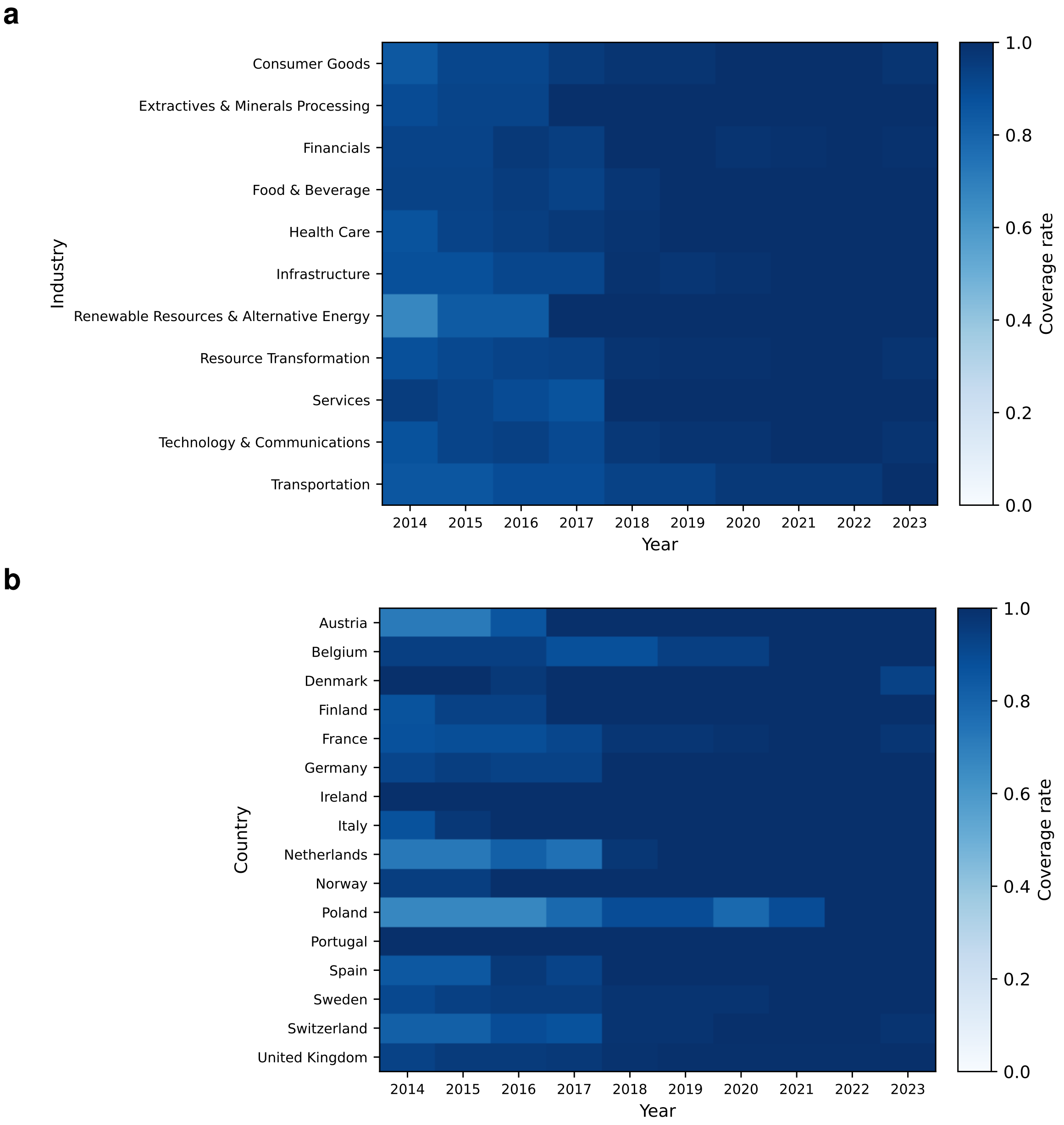

Fig. S5. Scope and annual coverage. a, Coverage by industry and year. Industries are categorized according to the Sustainable Industry Classification System (SICS), which was developed by the Sustainability Accounting Standards Board (SASB) to group companies into sectors with comparable exposure to sustainability-related risks and opportunities [1]. **b**, Coverage by country and year. Coverage is computed at the company–year level: for each STOXX 600 firm and year in the population table, a company–year is counted as covered if at least one report (e.g., annual report and/or sustainability report) is publicly available and included in our corpus for that company–year. Cells summarize the share of covered company–years within each **(a)** industry–year and **(b)** country–year. Coverage is high across both industries and countries and increases over time, converging to near-complete coverage for most industries and countries from roughly 2018 onward. Lower coverage is concentrated in the early years and in a small subset of industries and countries, consistent with more limited public availability of reports in the earlier part of the sample. Overall, the figure indicates broad and increasingly comprehensive coverage of STOXX Europe 600 firm-years in the corpus over the study period.

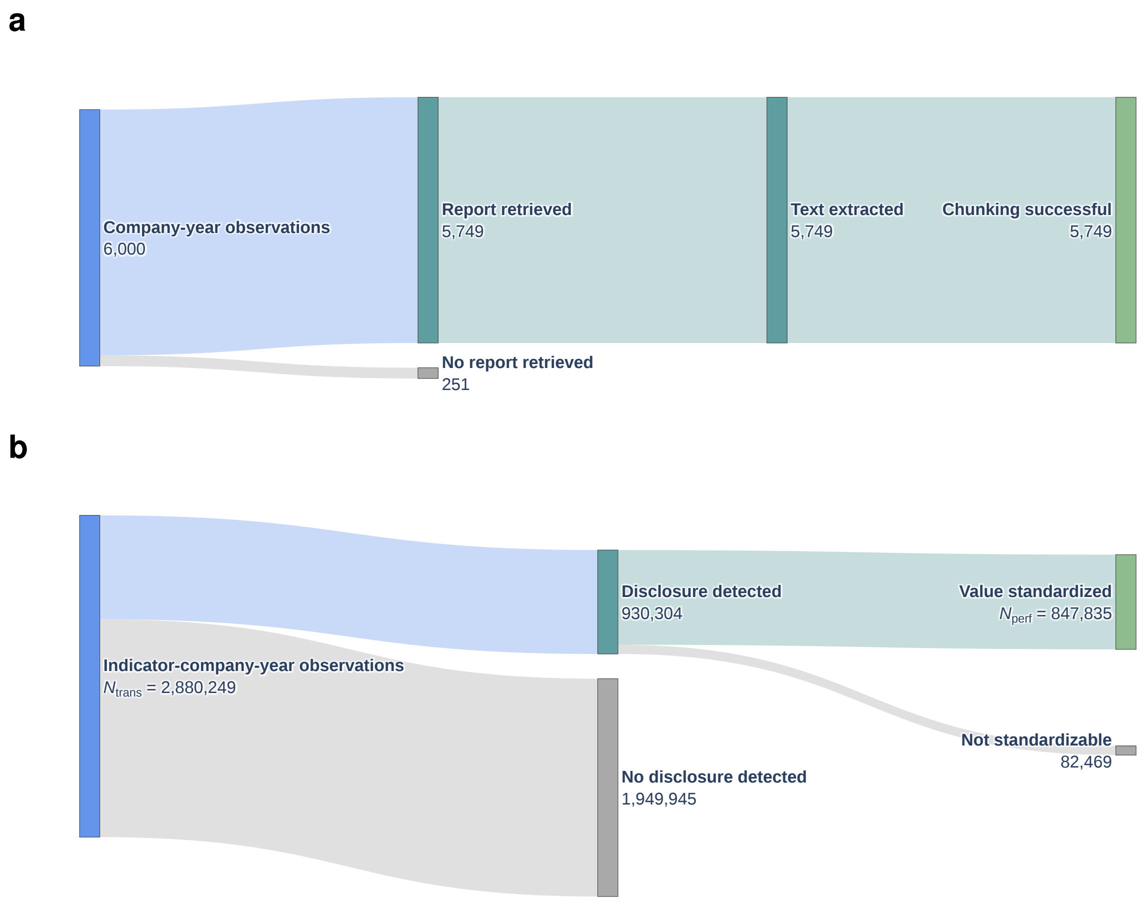

Fig. S6. Pipeline coverage analysis. **a**, Coverage of company–year observations across the end-to-end processing pipeline (successful report retrieval, text extraction, and chunking). Of all company–year observations under analysis (i.e., STOXX Europe 600, 2014–2023), 95.8% successfully pass all three stages and meet conservative minimum-quality gates designed to ensure substantive textual content (minimum extracted text length: 5,000 characters; minimum indexed chunks per vector store: 50). **b**, Coverage of indicator–company–year observations conditional on successful chunking, tracing indicator extraction from text, and subsequent normalization to a standardized, numeric representation. Among disclosed indicators, 88.6% yield a valid value that can be expressed in the EFRAG-specified unit after postprocessing.

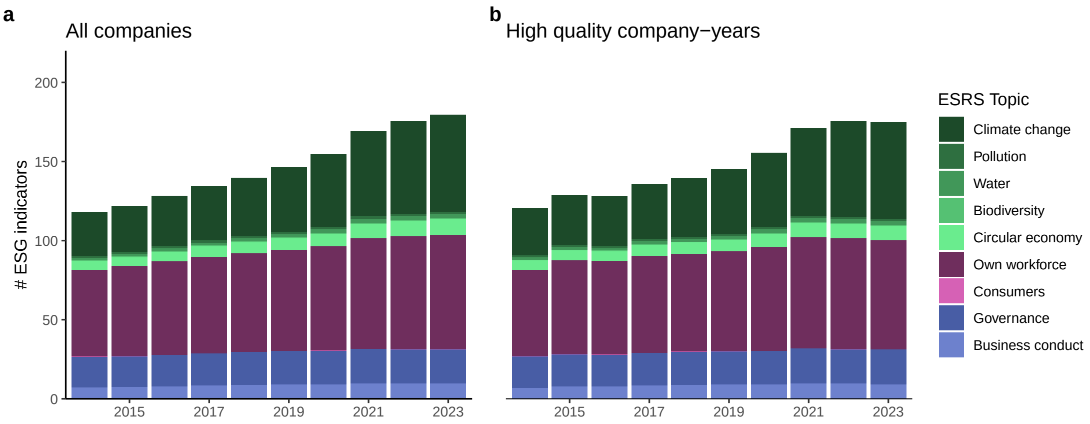

Fig. S7. Transparency trends for high-quality documents and full sample. Transparency trends are shown for the full sample in **a** and for the subset of company–year observations with high document text quality in **b**. High-quality documents are defined as texts that do not fall into the upper tercile on any of three proxies for poor text quality: newline density, boilerplate concentration, and the Fog index. Terciles are calculated within each year. Patterns remain consistent relative to the full sample, indicating that the main results are not primarily driven by improvements in document quality over time.

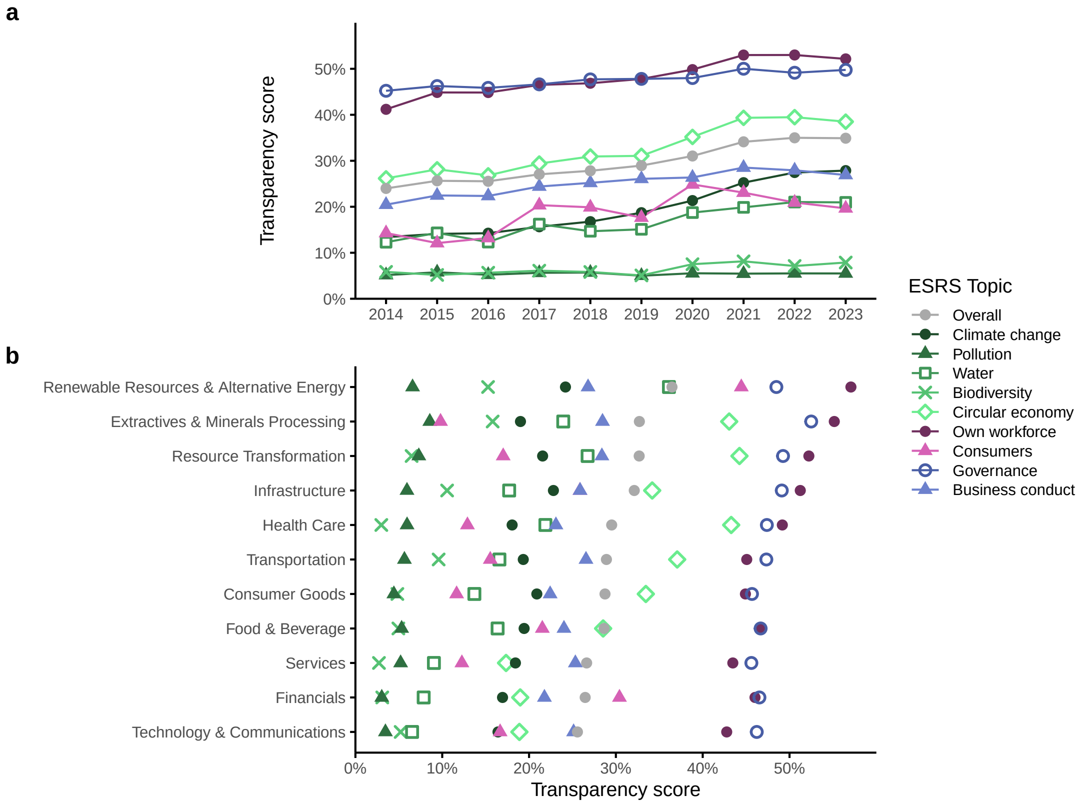

Fig. S8. Transparency across ESRS topics and industries for high-quality documents. Here, we re-estimate transparency patterns on a subset of company–year observations with high document text quality, defined as texts that do not fall into the upper tercile on any of three proxies for poor text quality: newline density, boilerplate concentration, and the Fog index. Terciles are calculated within each year. **a**, reports the transparency scores disclosed by ESRS topic between 2014 and 2023. **b**, shows the transparency scores by industry. Patterns generally mirror our findings relative to the full sample, indicating that the main results are not primarily driven by improvements in document quality over time.

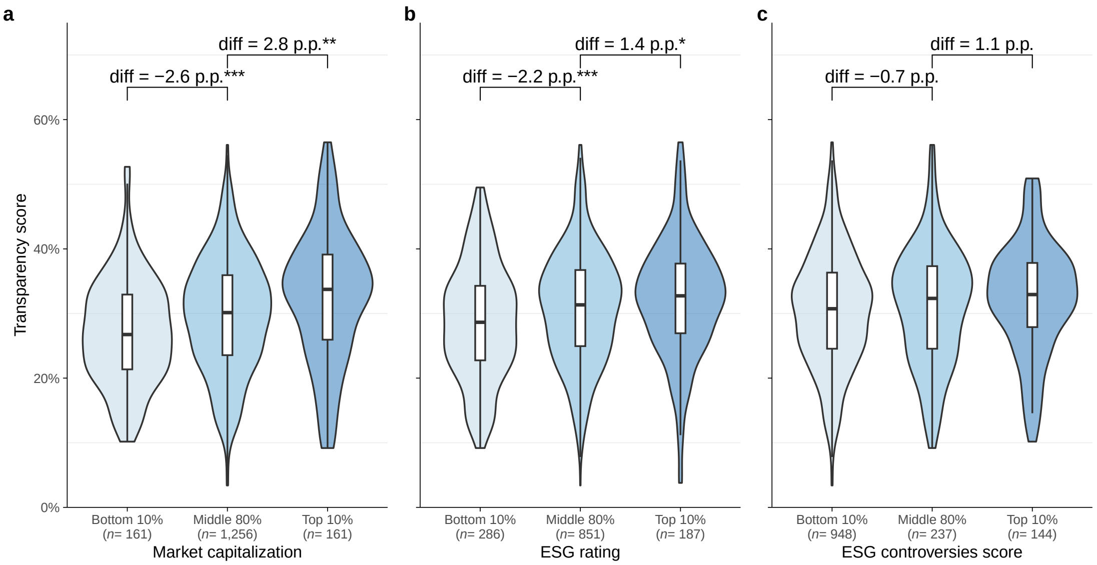

Fig. S9. Heterogeneity in ESG-related transparency across company characteristics for high-quality documents. Here, we re-estimate transparency patterns on a subset of company–year observations with high document text quality, defined as texts that do not fall into the upper tercile on any of three proxies for poor text quality: newline density, boilerplate concentration, and the Fog index. Terciles are calculated within each year. Shown are transparency scores (i.e., the relative number of disclosed indicators out of all 501 indicators) by **a**, market capitalization, **b**, ESG rating (based on MSCI ESG ratings [2] measured annually by company), and **c**, ESG controversies score (by Refinitiv [3]; measured annually by company). Patterns generally mirror our findings relative to the full sample, indicating that the main results are not primarily driven by improvements in document quality over time.

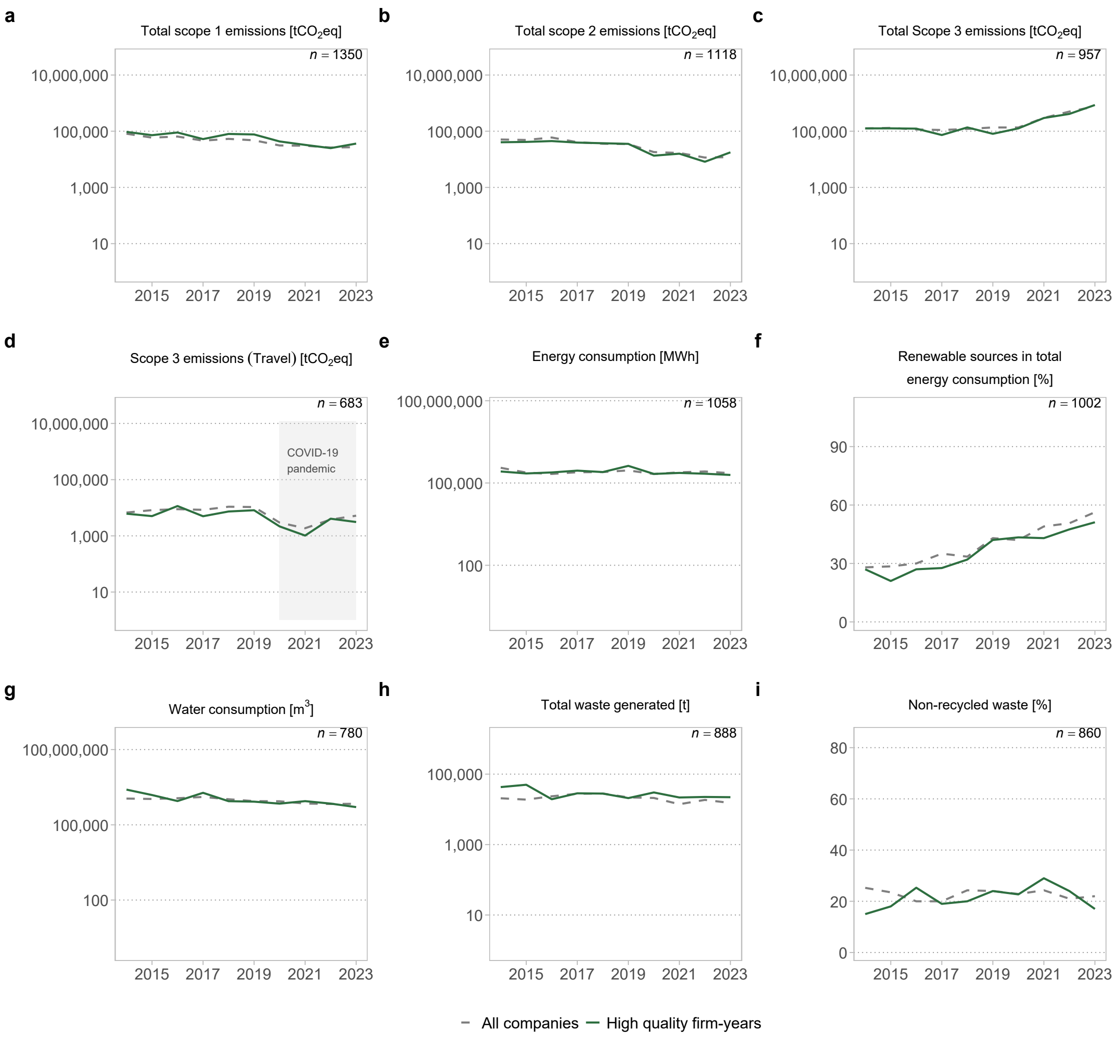

Fig. S10. Environmental performance trends for high-quality documents and full sample. Here, we re-estimate environmental performance trends on a subset of company–year observations with high document text quality, defined as texts that do not fall into the upper tercile on any of three proxies for poor text quality: newline density, boilerplate concentration, and the Fog index. Terciles are calculated within each year. Shown are **a**, total scope 1 emissions, **b**, total scope 2 emissions, **c**, total scope 3 emissions, **d**, scope 3 emissions (travel), **e**, energy consumption, **f**, the percentage of renewable sources in total energy consumption, **g**, water consumption, **h**, total waste generation, and **i**, the percentage of non-recycled waste. In this figure, n refers to the number of company–year observations. Note that logarithmic axes are used to better visualize different orders of magnitude. For indicators recorded in percent, we use linear scales. Patterns generally mirror our findings relative to the full sample, indicating that the main results are not primarily driven by improvements in document quality over time.

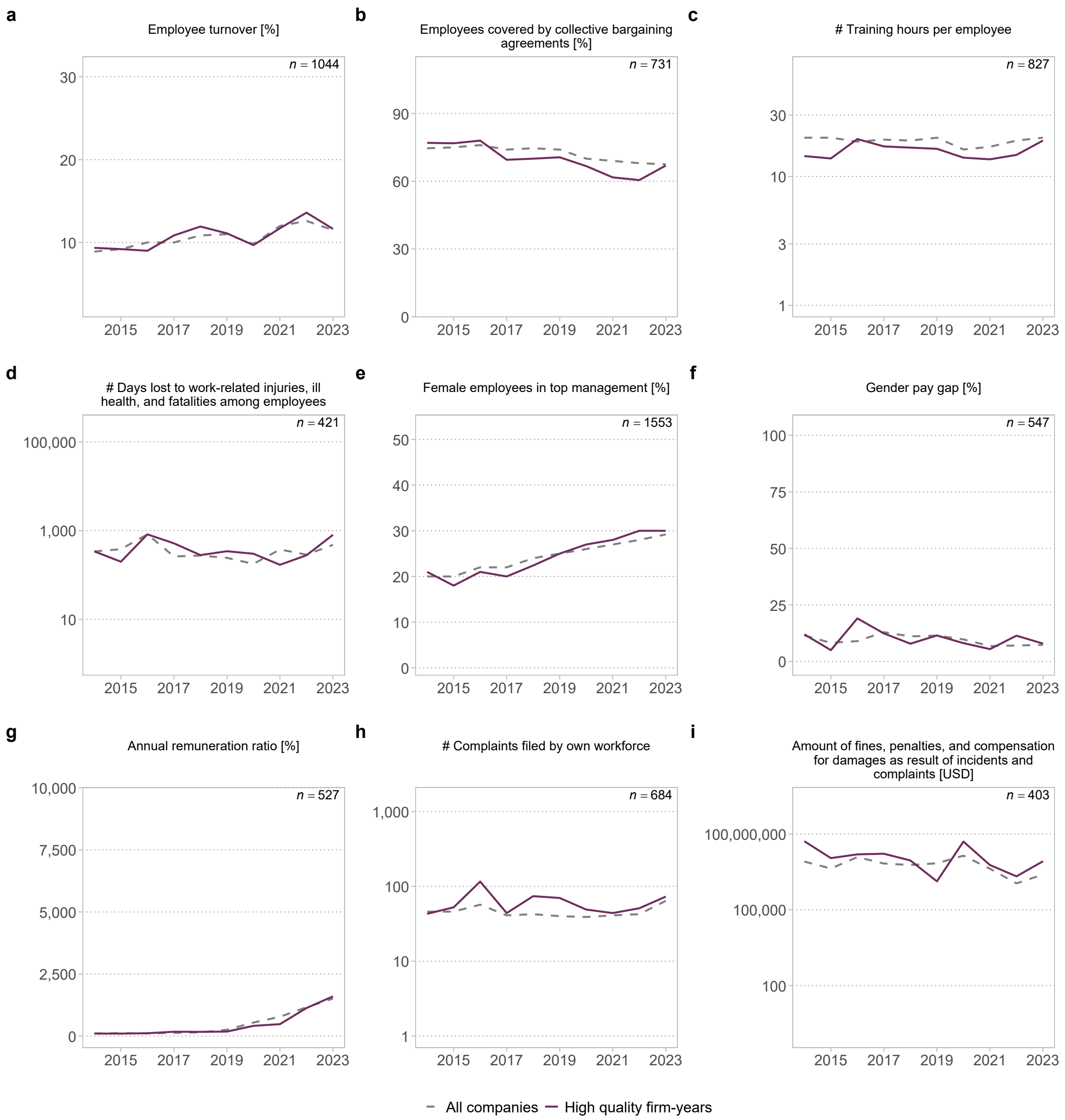

Fig. S11. Social performance trends for high-quality documents and full sample. Here, we re-estimate social performance trends on a subset of company–year observations with high document text quality, defined as texts that do not fall into the upper tercile on any of three proxies for poor text quality: newline density, boilerplate concentration, and the Fog index. Terciles are calculated within each year. Shown are **a**, employee turnover, **b**, the percentage of employees covered by collective bargaining agreements, **c**, the number of training hours per employee, **d**, the number of days lost to work-related injuries, ill health, and fatalities among employees, **e**, the percentage of female employees in top management, **f**, the gender pay gap, **g**, the annual remuneration ratio (i.e., defined as the ratio of total annual compensation for top executives compared to the median employee), **h**, the number of complaints filed by own workforce, and **i**, the number of fines, penalties, and compensation for damages as a result of incidents and complaints. In this figure, n refers to the number of company–year observations. Note that logarithmic axes are used to better visualize different orders of magnitude. For indicators recorded in percent, we use linear scales. Patterns generally mirror our findings relative to the full sample, indicating that the main results are not primarily driven by improvements in document quality over time.

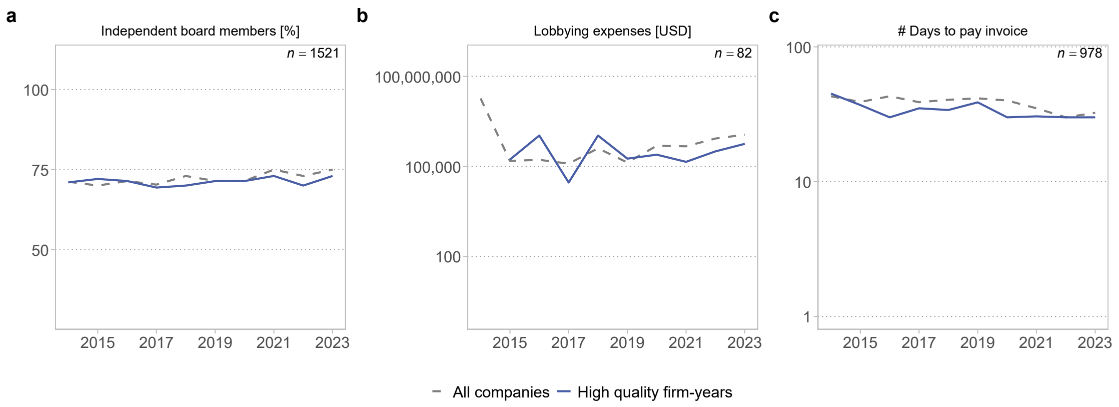

Fig. S12. Governance performance trends for high-quality documents and full sample. Here, we re-estimate governance performance trends on a subset of company–year observations with high document text quality, defined as texts that do not fall into the upper tercile on any of three proxies for poor text quality: newline density, boilerplate concentration, and the Fog index. Terciles are calculated within each year. Shown are **a**, the percentage of independent board members, **b**, lobbying expenses, and **c**, the number of days to pay invoice. In this figure, n refers to the number of company–year observations. Note that logarithmic axes are used to better visualize different orders of magnitude. For indicators recorded in percent, we use linear scales. Patterns generally mirror our findings relative to the full sample, indicating that the main results are not primarily driven by improvements in document quality over time.

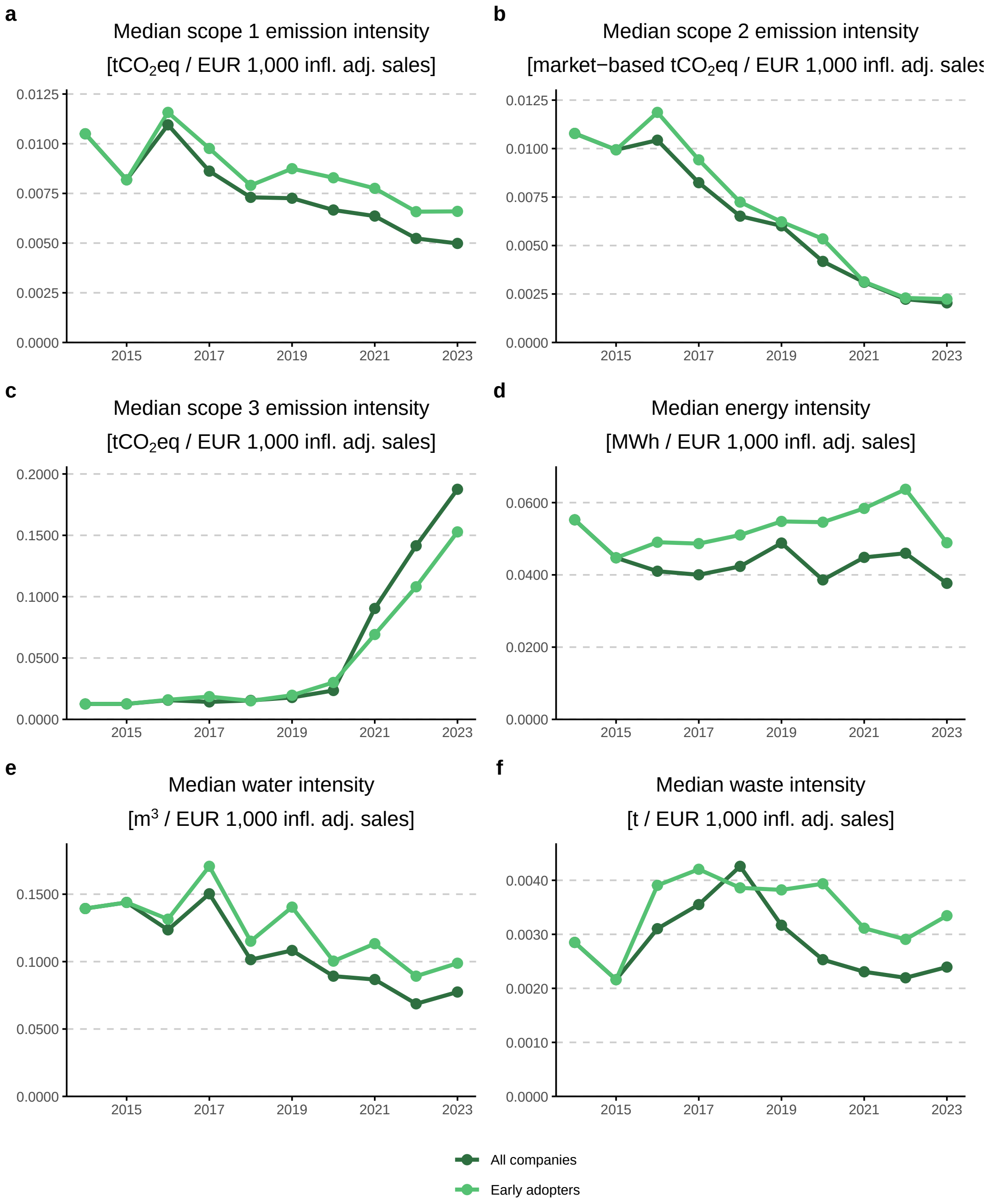

Fig. S13. Intensity trends (median) for selected environmental indicators for early adopters and full sample. Shown are **a**, median scope 1 emission intensity, **b**, median scope 2 emission intensity, **c**, median scope 3 emission intensity, **d**, median energy intensity, **e**, median water intensity, and **f**, median waste intensity. We define early adopting companies at indicator level as companies that start to report an indicator as early as 2014 or 2015. Intensities are calculated at company level as reported indicator values divided by revenues (sales) in EUR, adjusted for inflation (base year 2023).

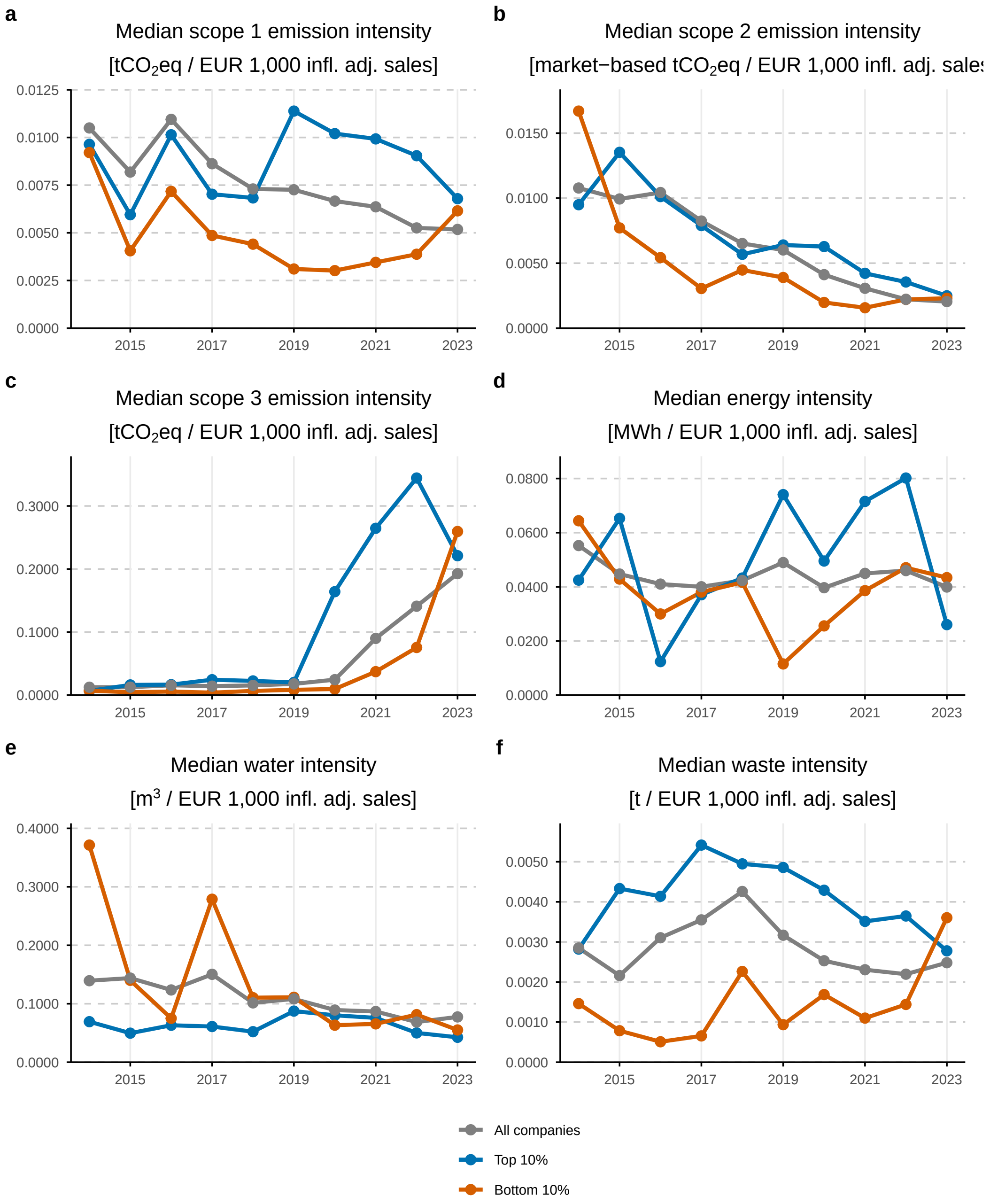

Fig. S14. Intensity trends (median) for selected environmental indicators by rating. Here, we compare the median intensities for selected environmental indicators between companies in the top-10% (bottom-10%) of lagged ESG ratings (provided by MSCI [2]) and the full sample. Shown are **a**, median scope 1 emission intensity, **b**, median scope 2 emission intensity, **c**, median scope 3 emission intensity, **d**, median energy intensity, **e**, median water intensity, and **f**, median waste intensity. Deciles are calculated on a yearly basis. Intensities are defined as units of an indicator as per EUR 1,000 in revenue.

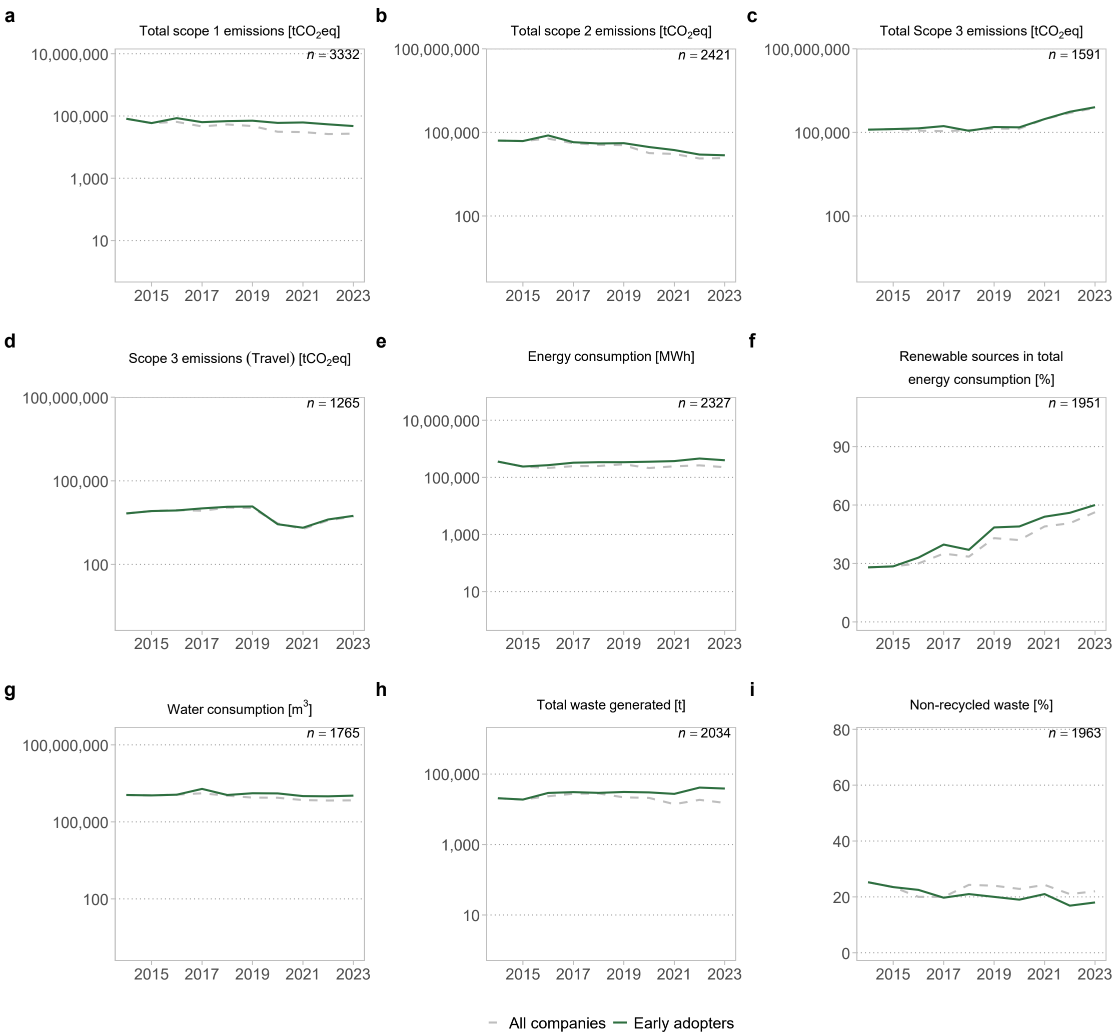

Fig. S15. Environmental indicators (median) for early adopters and full sample. Here, we compare medians for environmental indicators between early adopting companies (which we define as companies that start to report values for a specific indicator already in 2014 or 2015) and our full sample. Shown are **a**, total scope 1 emissions, **b**, total scope 2 emissions, **c**, total scope 3 emissions, **d**, scope 3 emissions (travel), **e**, energy consumption, **f**, the percentage of renewable sources in total energy consumption, **g**, water consumption, **h**, total waste generation, and **i**, the percentage of non-recycled waste. We report the number of company–year observations for early adopting companies in the upper-right corner of each panel. In this figure, n refers to the number of observations of early adopting companies. Note that logarithmic axes are used to better visualize different orders of magnitude. For indicators recorded in percent, we use linear scales.

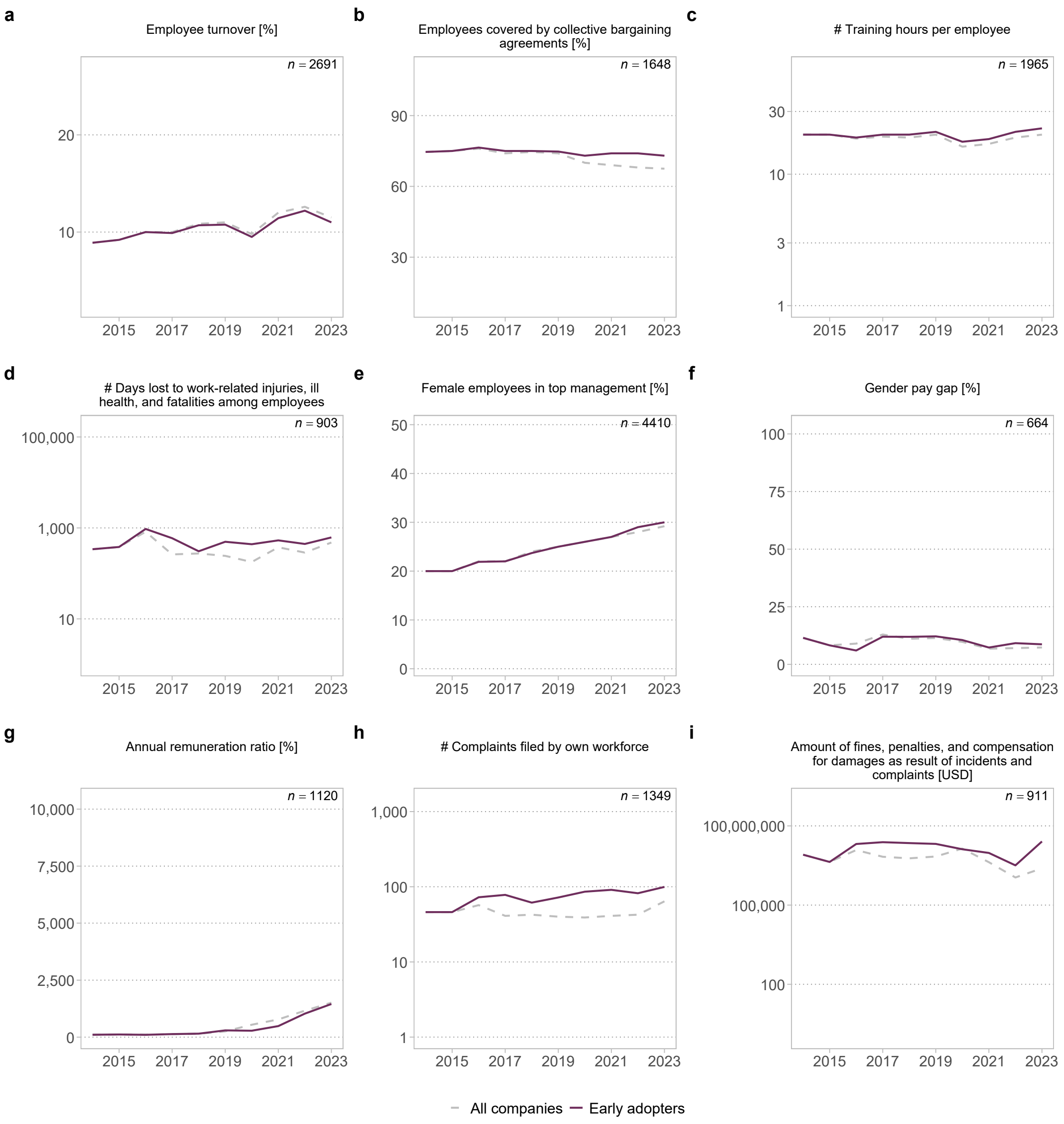

Fig. S16. Social indicators (median) for early adopters and full sample. Here, we compare medians for social indicators between early adopting companies (which we define as companies that start to report values for a specific indicator already in 2014 or 2015) and our full sample. Shown are **a**, employee turnover, **b**, the percentage of employees covered by collective bargaining agreements, **c**, the number of training hours per employee, **d**, the number of days lost to work-related injuries, ill health, and fatalities among employees, **e**, the percentage of female employees in top management, **f**, the gender pay gap, **g**, the annual remuneration ratio (i.e., defined as the ratio of total annual compensation for top executives compared to the median employee), **h**, the number of complaints filed by own workforce, and **i**, the number of fines, penalties, and compensation for damages as a result of incidents and complaints. We report the number of company–year observations for early adopting companies in the upper-right corner of each panel. In this figure, n refers to the number of observations of early adopting companies. Note that logarithmic axes are used to better visualize different orders of magnitude. For indicators recorded in percent, we use linear scales.

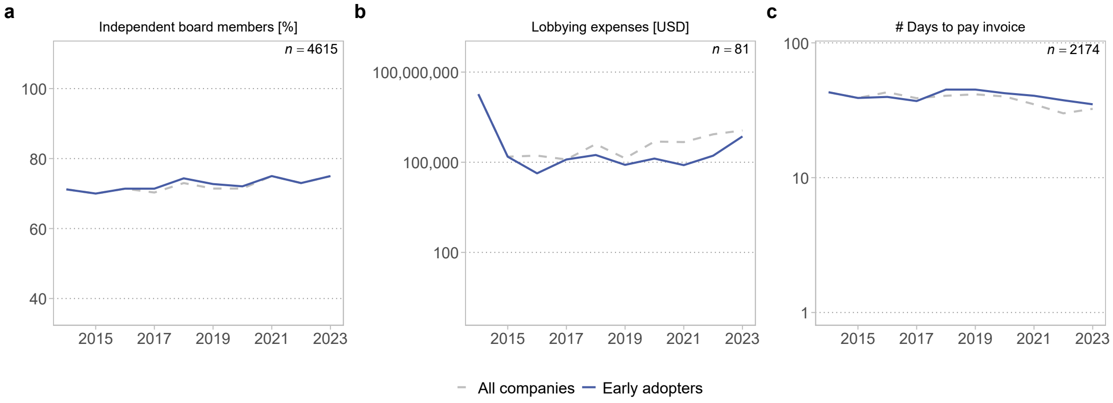

Fig. S17. Governance indicators (median) for early adopters and full sample. Here, we compare medians for governance indicators between early adopting companies (which we define as companies that start to report values for a specific indicator already in 2014 or 2015) and our full sample. Shown are **a**, the percentage of independent board members, **b**, lobbying expenses, and **c**, the number of days to pay invoice. We report the number of company–year observations for early adopting companies in the upper-right corner of each panel. In this figure, n refers to the number of observations of early adopting companies. Note that logarithmic axes are used to better visualize different orders of magnitude. For indicators recorded in percent, we use linear scales.

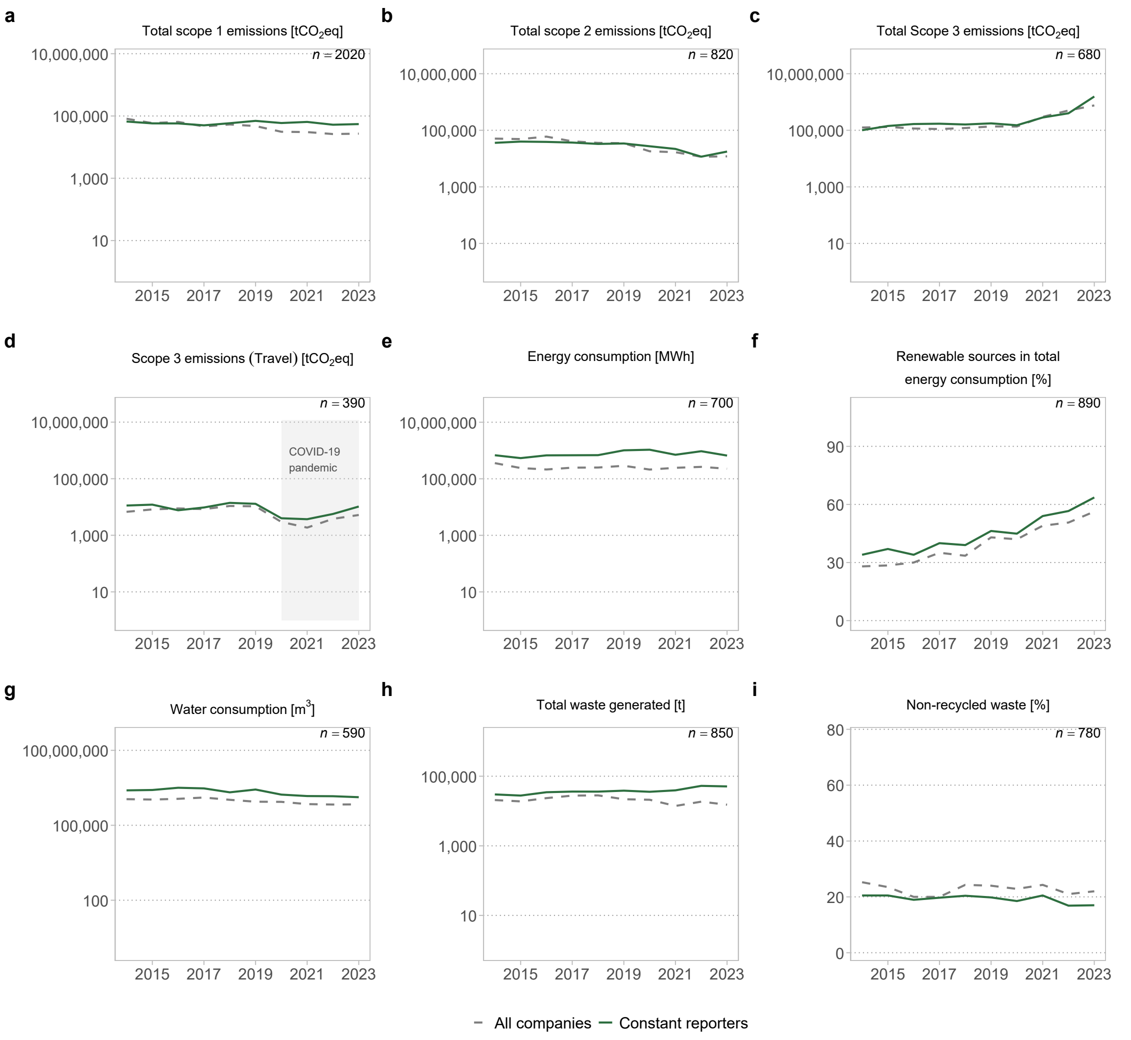

Fig. S18. Environmental indicators (median) for constant reporters and full sample. Here, we compare medians for environmental indicators between constant reporters, i.e., companies that report on a given metric for all years within the sample period, and our full sample. Shown are **a**, total scope 1 emissions, **b**, total scope 2 emissions, **c**, total scope 3 emissions, **d**, scope 3 emissions (travel), **e**, energy consumption, **f**, the percentage of renewable sources in total energy consumption, **g**, water consumption, **h**, total waste generation, and **i**, the percentage of non-recycled waste. We report the number of company–year observations for constant reporters in the upper-right corner of each panel. In this figure, n refers to the number of company–year observations of constant reporters. Note that logarithmic axes are used to better visualize different orders of magnitude. For indicators recorded in percent, we use linear scales. Although this subset is more restrictive, the results closely mirror our findings for early adopting companies and the full sample.

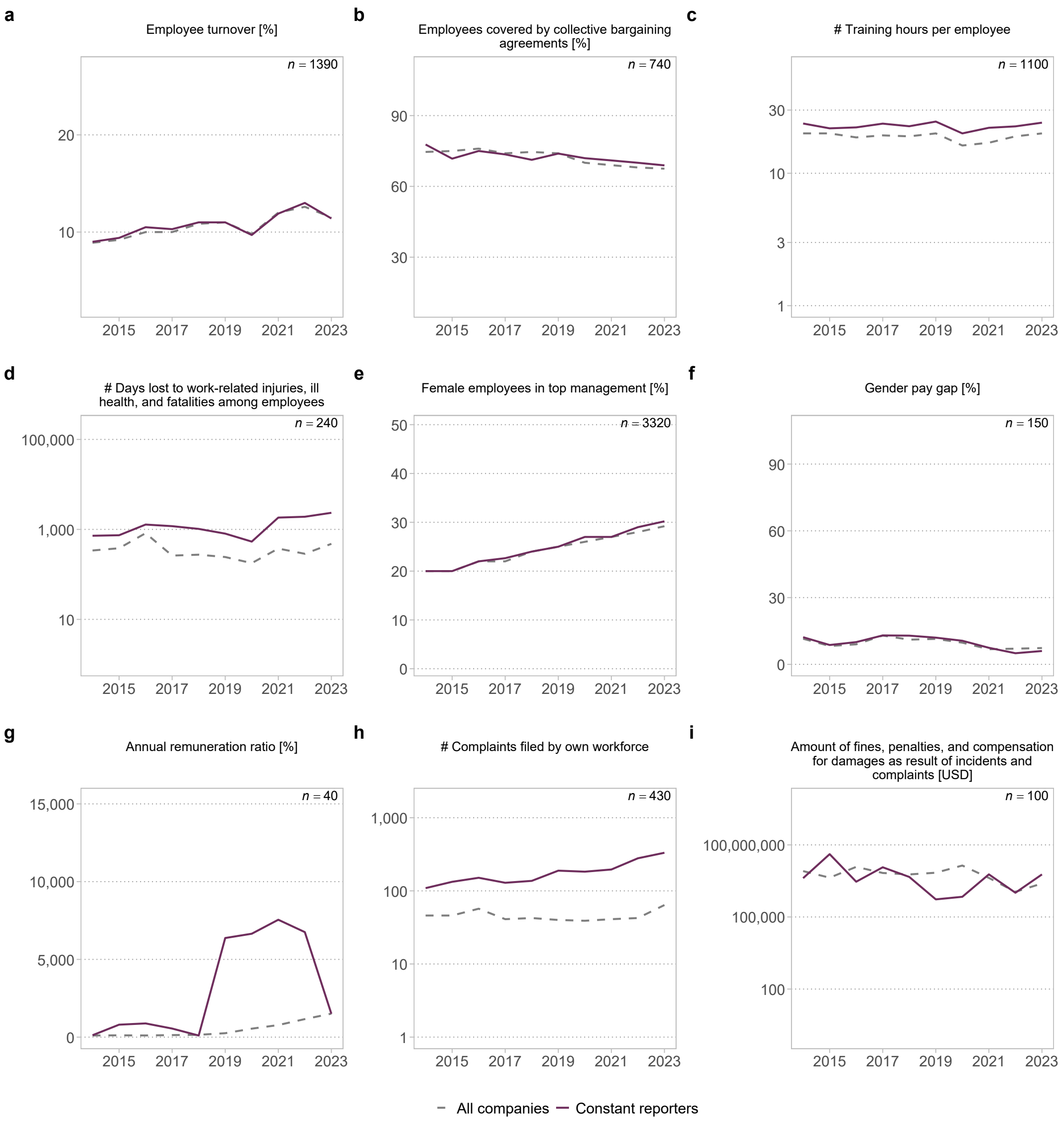

Fig. S19. Social indicators (median) for constant reporters and full sample. Here, we compare medians for social indicators between constant reporters, i.e., companies that report on a given metric for all years within the sample period, and our full sample. Shown are **a**, employee turnover, **b**, the percentage of employees covered by collective bargaining agreements, **c**, the number of training hours per employee, **d**, the number of days lost to work-related injuries, ill health, and fatalities among employees, **e**, the percentage of female employees in top management, **f**, the gender pay gap, **g**, the annual remuneration ratio (i.e., defined as the ratio of total annual compensation for top executives compared to the median employee), **h**, the number of complaints filed by own workforce, and **i**, the number of fines, penalties, and compensation for damages as a result of incidents and complaints. We report the number of company–year observations for constant reporters in the upper-right corner of each panel. In this figure, n refers to the number of company–year observations of constant reporters. Note that logarithmic axes are used to better visualize different orders of magnitude. For indicators recorded in percent, we use linear scales. Although this subset is more restrictive, the results closely mirror our findings for early adopting companies and the full sample, with the exception of the annual remuneration ratio, where the small sample of constant reporters ($n = 4$) limits the representativeness of the observed trend.

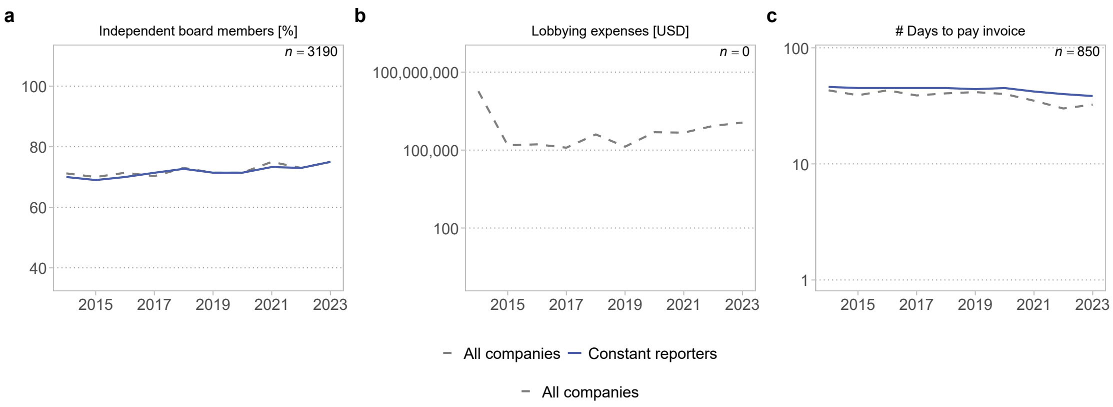

Fig. S20. Governance indicators (median) for constant reporters and full sample. Here, we compare medians for governance indicators between constant reporters (companies that report on a given metric for all years within the sample period) and our full sample. Shown are **a**, the percentage of independent board members, **b**, lobbying expenses, and **c**, the number of days to pay invoice. We report the number of company–year observations for constant reporters in the upper-right corner of each panel. In this figure, n refers to the number of company–year observations of constant reporters. Note that logarithmic axes are used to better visualize different orders of magnitude. For indicators recorded in percent, we use linear scales. Although this subset is more restrictive, the results closely mirror our findings for early adopting companies and the full sample. Our full sample does not contain any constant reporters for lobbying expenses.

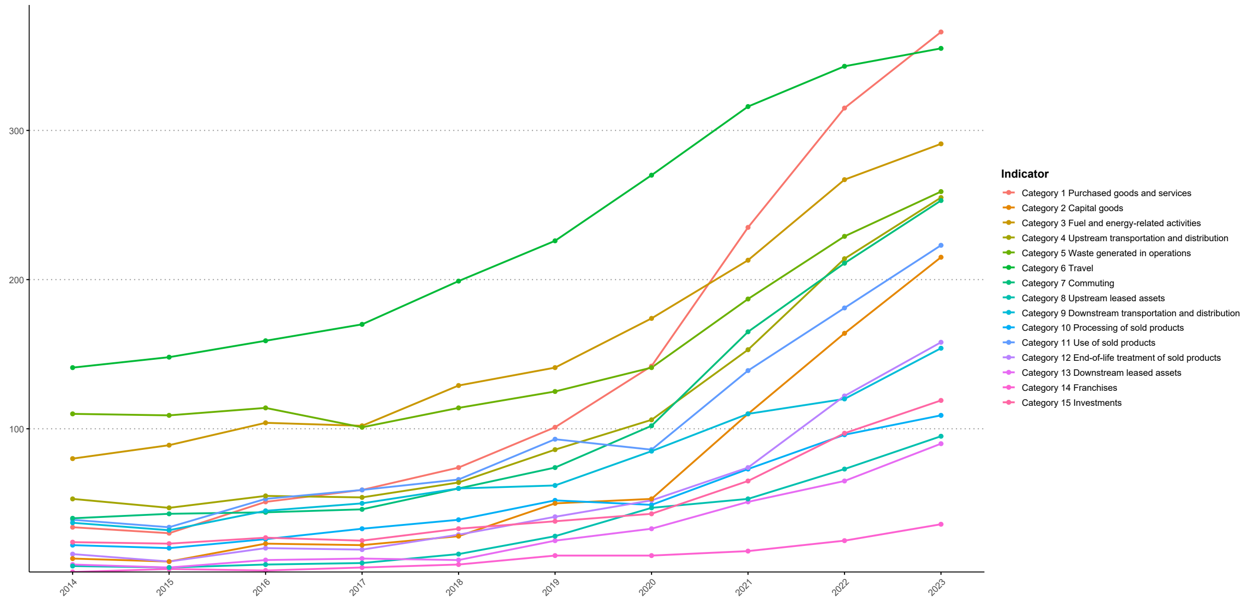

Fig. S21. # Observations per year by scope 3 greenhouse gas emissions category. This figure shows the number of company–year observations disclosing values for each scope 3 emissions category over time. We distinguish 15 reporting categories following the GHG Protocol classification. Categories are labeled and ordered by their numeric identifier. We observe substantial variation in disclosure rates across categories, with some categories being consistently more frequently reported than others.

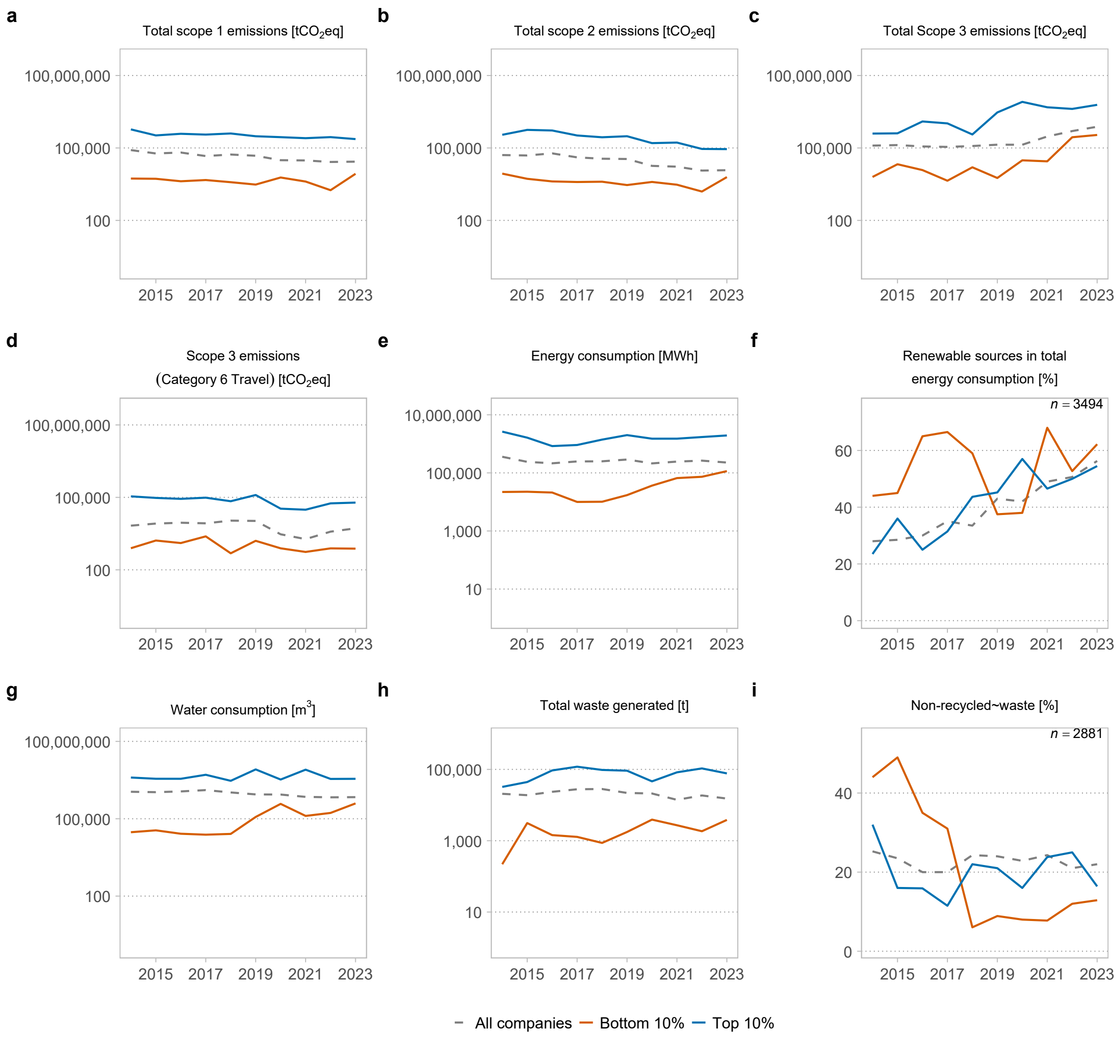

Fig. S22. Differences in environmental indicators by market capitalization. Here, we compare the medians for environmental indicators between companies in the top-10% (bottom-10%) of lagged market capitalization and the rest of the sample. Shown are **a**, total scope 1 emissions, **b**, total scope 2 emissions, **c**, total scope 3 emissions, **d**, scope 3 emissions (travel), **e**, energy consumption, **f**, the percentage of renewable sources in total energy consumption, **g**, water consumption, **h**, total waste generation, and **i**, the percentage of non-recycled waste. Deciles are calculated on a yearly basis. We report the number of company–year observations in the upper-right corner of each panel. Note that logarithmic axes are used to better visualize different orders of magnitude. For indicators recorded in percent, we use linear scales.

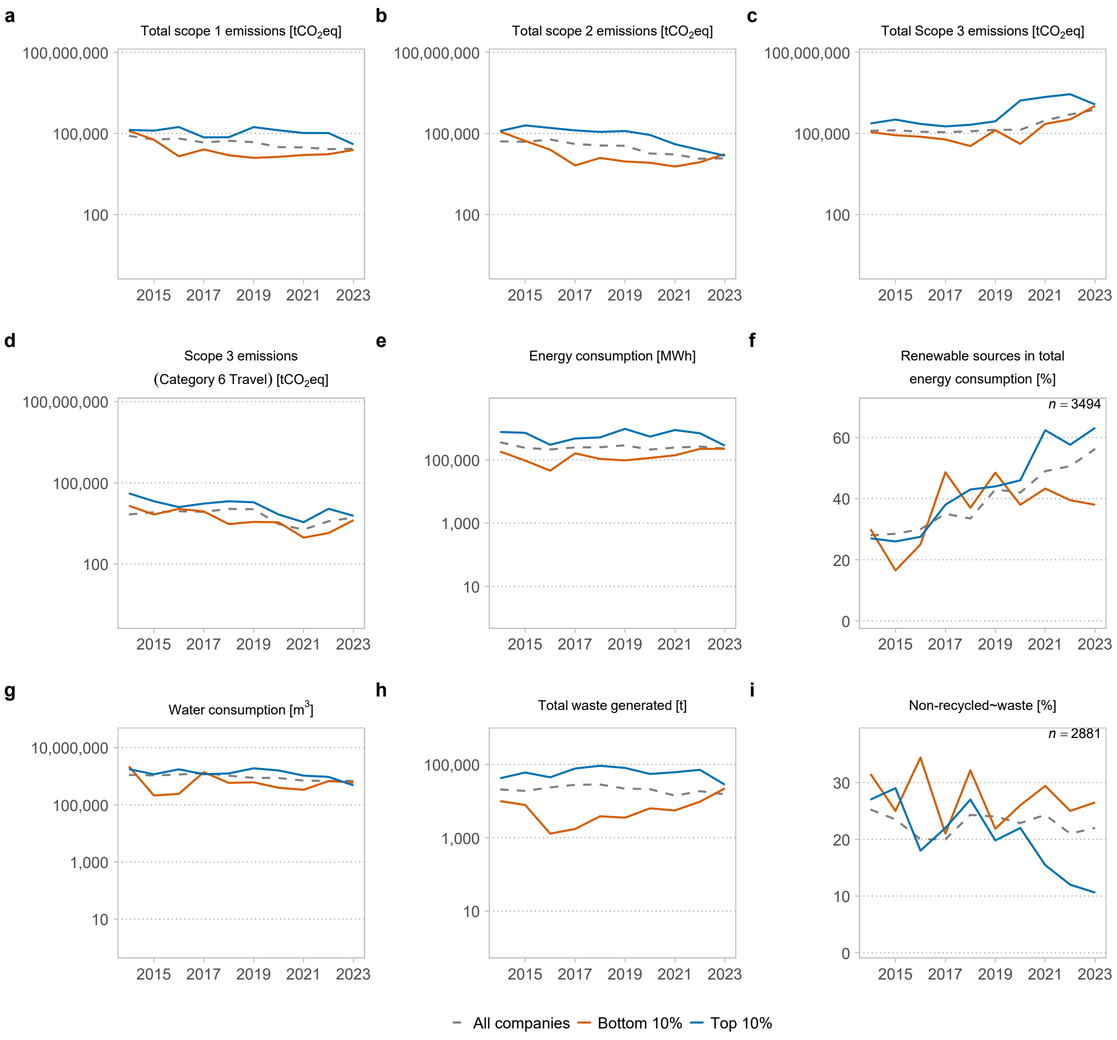

Fig. S23. Differences in environmental indicators by ESG rating. Here, we compare the medians for environmental indicators between companies in the top-10% (bottom-10%) of lagged ESG ratings and the rest of the sample. ESG ratings are sourced from MSCI [2]. Shown are **a**, total scope 1 emissions, **b**, total scope 2 emissions, **c**, total scope 3 emissions, **d**, scope 3 emissions (travel), **e**, energy consumption, **f**, the percentage of renewable sources in total energy consumption, **g**, water consumption, **h**, total waste generation, and **i**, the percentage of non-recycled waste. Deciles are calculated on a yearly basis. We report the number of company–year observations in the upper-right corner of each panel. Note that logarithmic axes are used to better visualize different orders of magnitude. For indicators recorded in percent, we use linear scales.

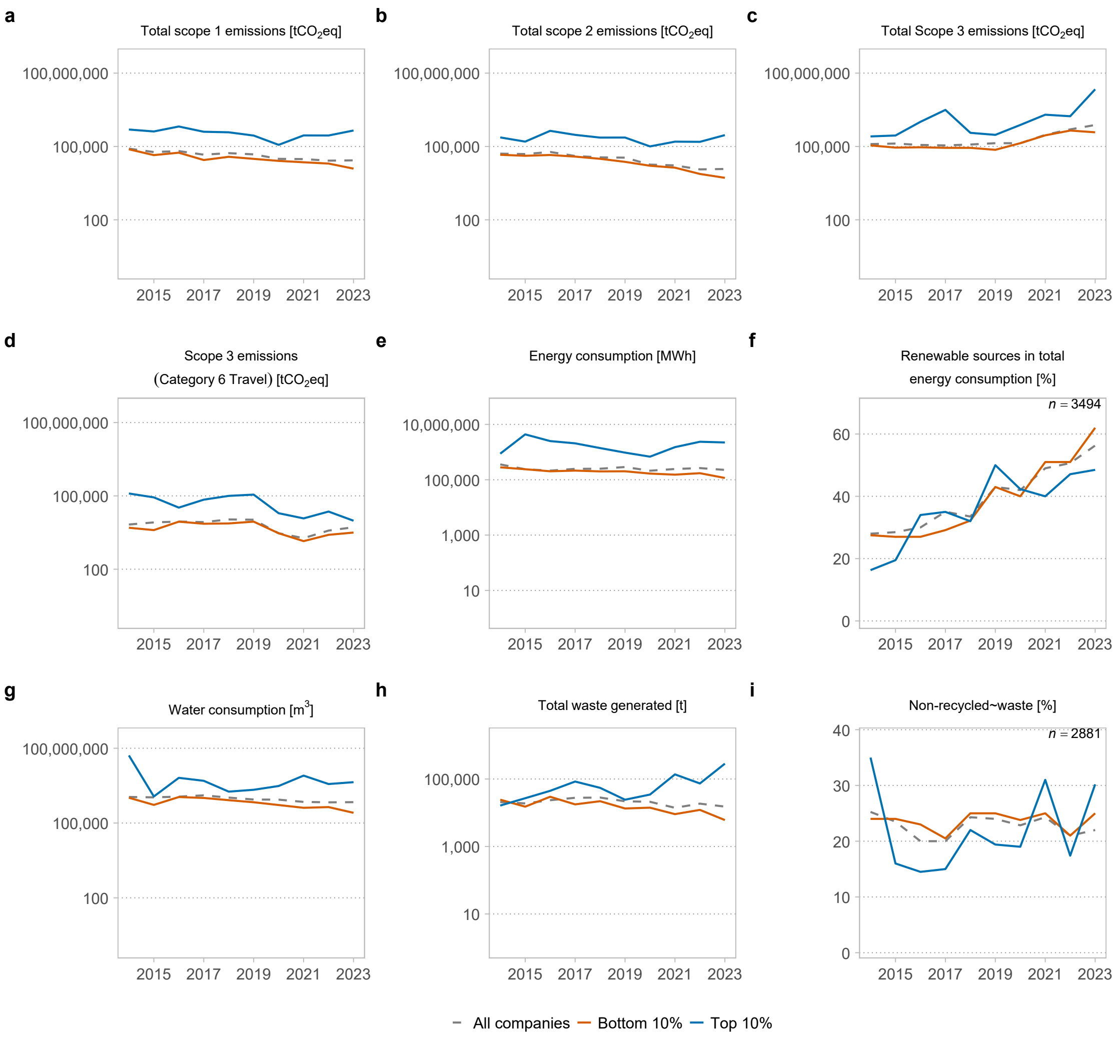

Fig. S24. Differences in environmental indicators by ESG controversies score. Here, we compare the medians for environmental indicators between companies in the top-10% (bottom-10%) of lagged ESG controversies scores and the rest of the sample. Controversies scores are sourced from Refinitiv [4]. We inverse-code the score such that companies in the top-10% of controversies scores have more controversies. Shown are **a**, total scope 1 emissions, **b**, total scope 2 emissions, **c**, total scope 3 emissions, **d**, scope 3 emissions (travel), **e**, energy consumption, **f**, the percentage of renewable sources in total energy consumption, **g**, water consumption, **h**, total waste generation, and **i**, the percentage of non-recycled waste. Deciles are calculated on a yearly basis. We report the number of company–year observations in the upper-right corner of each panel. Note that logarithmic axes are used to better visualize different orders of magnitude. For indicators recorded in percent, we use linear scales.

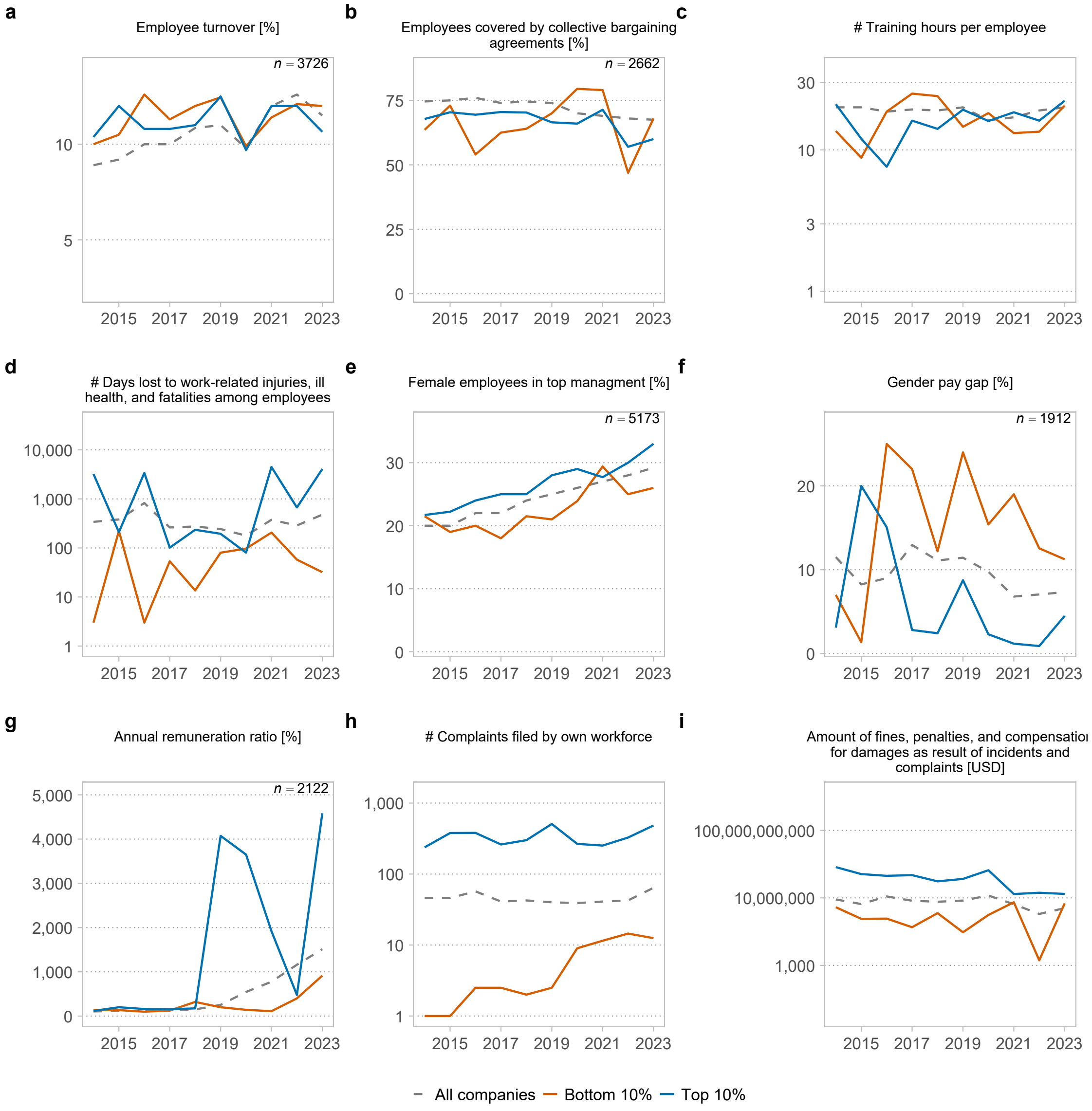

Fig. S25. Differences in social indicators by market capitalization. Here, we compare the medians for social indicators between companies in the top-10% (bottom-10%) of lagged market capitalization and the rest of the sample. Shown are **a**, employee turnover, **b**, the percentage of employees covered by collective bargaining agreements, **c**, the number of training hours per employee, **d**, the number of days lost to work-related injuries, ill health, and fatalities among employees, **e**, the percentage of female employees in top management, **f**, the gender pay gap, **g**, the annual remuneration ratio (i.e., defined as the ratio of total annual compensation for top executives compared to the median employee), **h**, the number of complaints filed by own workforce, and **i**, the number of fines, penalties, and compensation for damages as a result of incidents and complaints. Deciles are calculated on a yearly basis. We report the number of company–year observations in the upper-right corner of each panel. Note that logarithmic axes are used to better visualize different orders of magnitude. For indicators recorded in percent, we use linear scales.

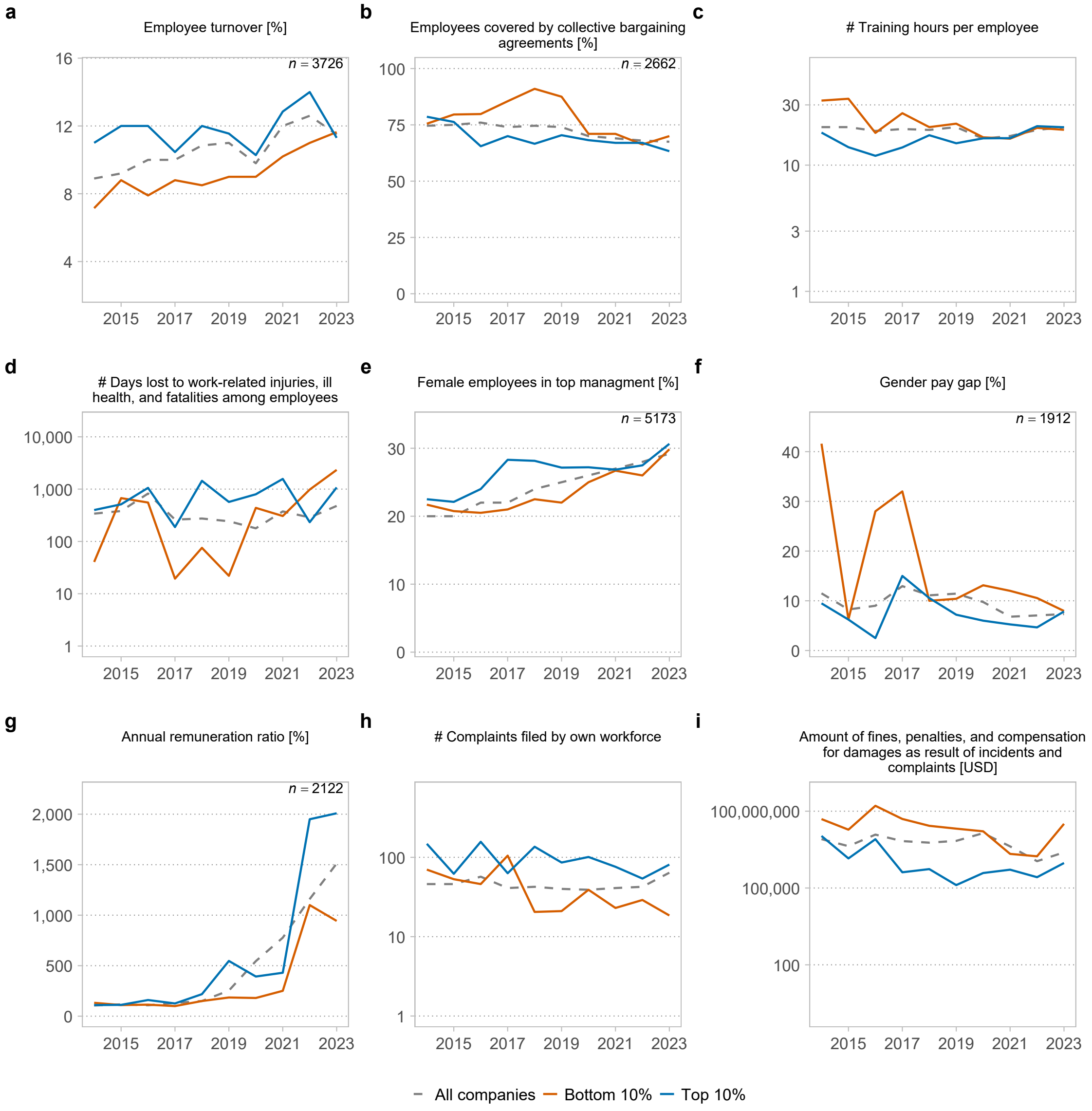

Fig. S26. Differences in social indicators by ESG rating. Here, we compare the medians for social indicators between companies in the top-10% (bottom-10%) of lagged ESG ratings and the rest of the sample. ESG ratings are sourced from MSCI [2]. Shown are **a**, employee turnover, **b**, the percentage of employees covered by collective bargaining agreements, **c**, the number of training hours per employee, **d**, the number of days lost to work-related injuries, ill health, and fatalities among employees, **e**, the percentage of female employees in top management, **f**, the gender pay gap, **g**, the annual remuneration ratio (i.e., defined as the ratio of total annual compensation for top executives compared to the median employee), **h**, the number of complaints filed by own workforce, and **i**, the number of fines, penalties, and compensation for damages as a result of incidents and complaints. Deciles are calculated on a yearly basis. We report the number of company–year observations in the upper-right corner of each panel. Note that logarithmic axes are used to better visualize different orders of magnitude. For indicators recorded in percent, we use linear scales.

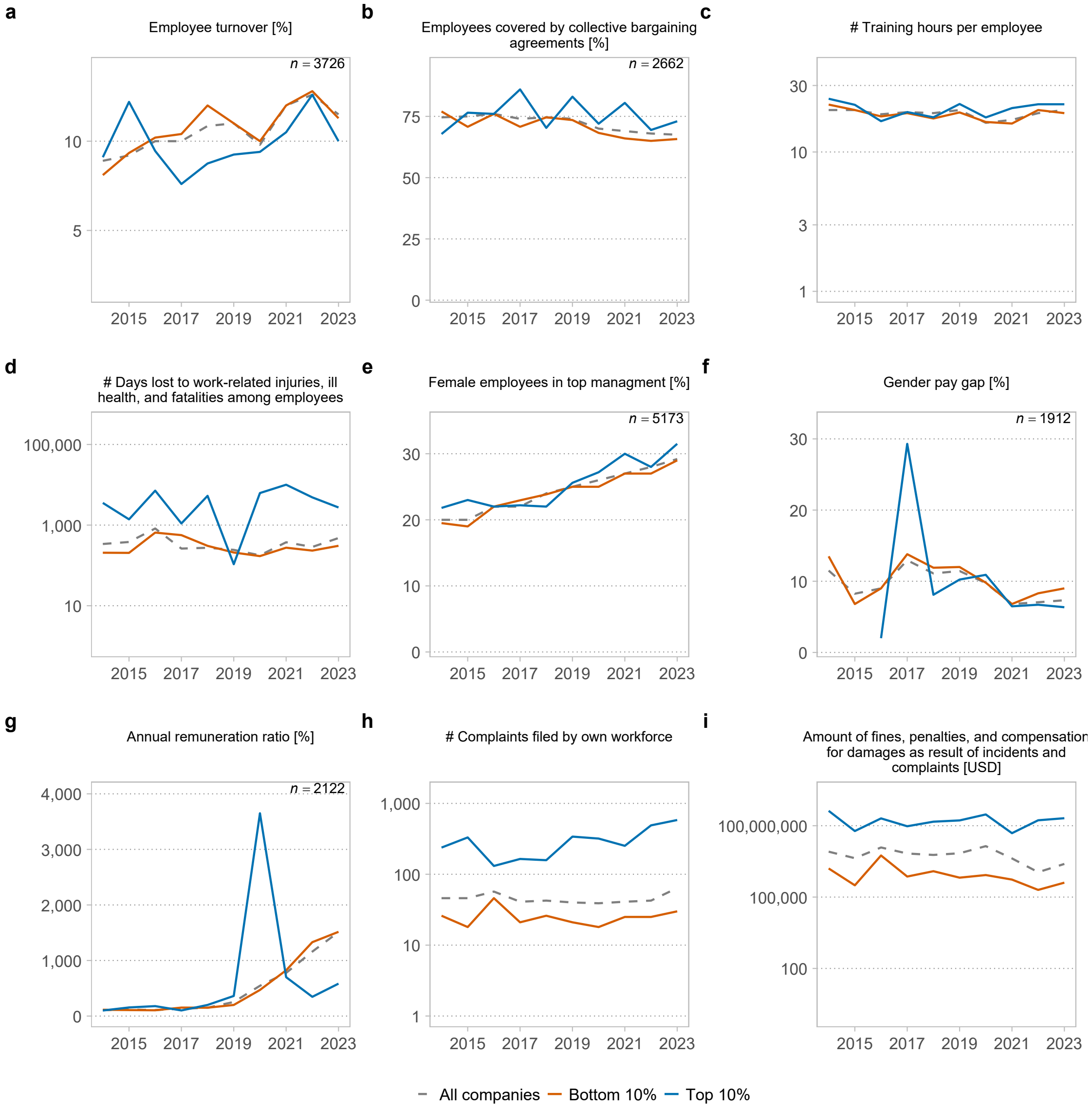

Fig. S27. Differences in social indicators by ESG controversies score. Here, we compare the medians for social indicators between companies in the top-10% (bottom-10%) of lagged ESG controversies scores and the rest of the sample. Controversies scores are sourced from Refinitiv [4]. We inverse-code the score such that companies in the top-10% of controversies scores have more controversies. Shown are **a**, employee turnover, **b**, the percentage of employees covered by collective bargaining agreements, **c**, the number of training hours per employee, **d**, the number of days lost to work-related injuries, ill health, and fatalities among employees, **e**, the percentage of female employees in top management, **f**, the gender pay gap, **g**, the annual remuneration ratio (i.e., defined as the ratio of total annual compensation for top executives compared to the median employee), **h**, the number of complaints filed by own workforce, and **i**, the number of fines, penalties, and compensation for damages as a result of incidents and complaints. Deciles are calculated on a yearly basis. We report the number of company–year observations in the upper-right corner of each panel. Note that logarithmic axes are used to better visualize different orders of magnitude. For indicators recorded in percent, we use linear scales.

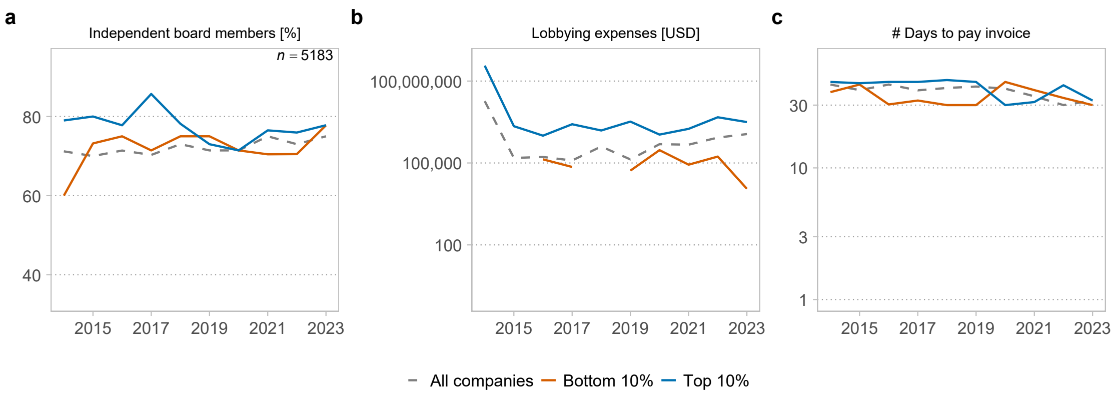

Fig. S28. Differences in governance indicators by market capitalization. Here, we compare the medians for governance indicators between companies in the top-10% (bottom-10%) of lagged market capitalization and the rest of the sample. Deciles are calculated on a yearly basis. Shown are **a**, the percentage of independent board members, **b**, lobbying expenses, and **c**, the number of days to pay invoice. We report the number of company–year observations in the upper-right corner of each panel. Note that logarithmic axes are used to better visualize different orders of magnitude. For indicators recorded in percent, we use linear scales.

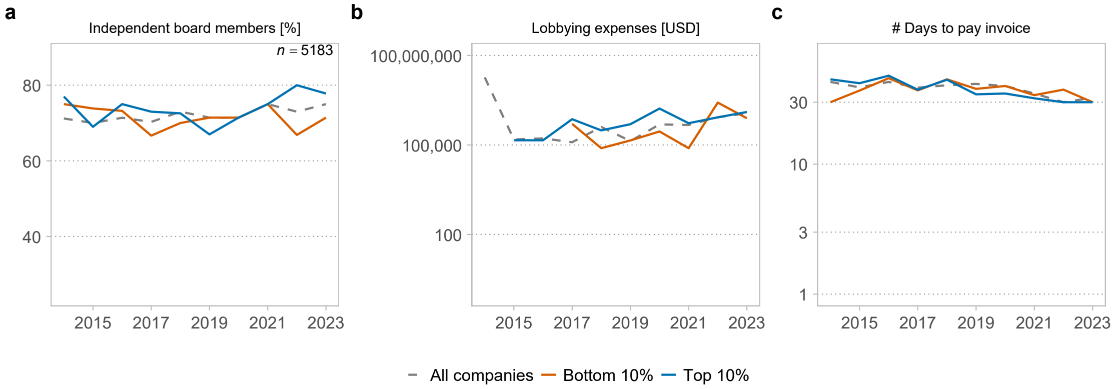

Fig. S29. Differences in governance indicators by ESG rating. Here, we compare the medians for governance indicators between companies in the top-10% (bottom-10%) of lagged ESG ratings and the rest of the sample. ESG ratings are sourced from MSCI [2]. Deciles are calculated on a yearly basis. Shown are **a**, the percentage of independent board members, **b**, lobbying expenses, and **c**, the number of days to pay invoice. We report the number of company–year observations in the upper-right corner of each panel. Note that logarithmic axes are used to better visualize different orders of magnitude. For indicators recorded in percent, we use linear scales.

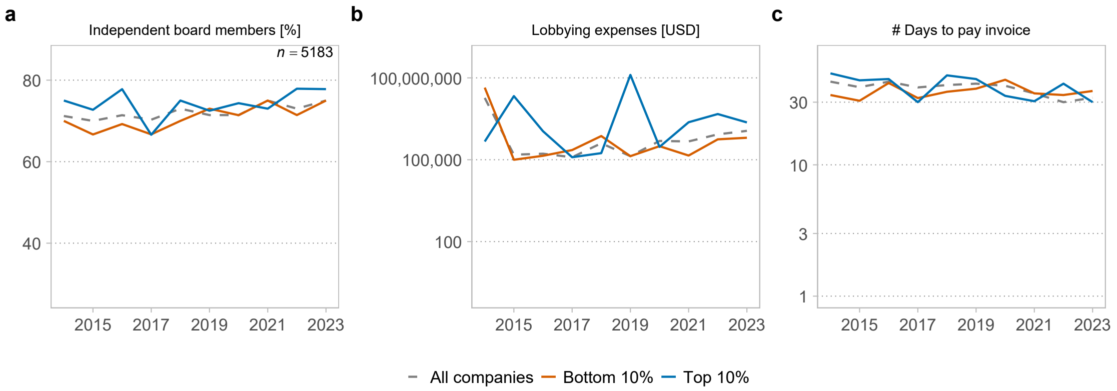

Fig. S30. Differences in governance indicators by ESG controversies score. Here, we compare the medians for governance indicators between companies in the top-10% (bottom-10%) of lagged ESG controversies scores and the rest of the sample. Controversies scores are sourced from Refinitiv [4]. We inverse-code the score such that companies in the top-10% of controversies scores have more controversies. Deciles are calculated on a yearly basis. Shown are **a**, the percentage of independent board members, **b**, lobbying expenses, and **c**, the number of days to pay invoice. We report the number of company–year observations in the upper-right corner of each panel. Note that logarithmic axes are used to better visualize different orders of magnitude. For indicators recorded in percent, we use linear scales.

Supplementary Tables

Table S1: List of companies by name and country, grouped by SICS sector

Name	Country	Name	Country
**Consumer Goods**			
Adidas	Germany	B&M	United Kingdom
Beiersdorf	Germany	Brunello Cucinelli	Italy
Bunzl	United Kingdom	Burberry	United Kingdom
Christian Dior	France	D'IeterenGroup	Belgium
Diploma	United Kingdom	Dufry	Switzerland
Electrolux	Sweden	Essity	Sweden
Games Workshop	United Kingdom	Grafton Group	United Kingdom
H&M	Sweden	Haleon	United Kingdom
Hellofresh	Germany	Henkel	Germany
Hermes International	France	Howdens	United Kingdom
Hugo Boss	Germany	Inchcape	United Kingdom
Inditex	Spain	JD Sports Fashion	United Kingdom
Kering	France	Kingfisher	United Kingdom
L'Oreal	France	LPP	Poland
LVMH Moet Hennessy Louis Vuitton	France	Marks and Spencer Group	United Kingdom
Moncler	Italy	Next	United Kingdom
Pandora	Denmark	Puma	Germany
Reckitt	United Kingdom	Richemont	Switzerland
Rockwool	Denmark	SEB	France
Swatch Group	Switzerland	Thule Group	Sweden
Travis Perkins	United Kingdom	Unilever	United Kingdom
WHSmith	United Kingdom	Watches of Switzerland Group	United Kingdom
Zalando	Germany	Zalando	Germany
**Extractives & Minerals Processing**			
AkerBP	Norway	Anglo American	United Kingdom
Antofagasta	United Kingdom	ArcelorMittal	France
Aurubis	Germany	BP	United Kingdom
Boliden	Sweden	CRH	Ireland

Table S1: List of companies by name and country, grouped by SICS sector *(continued)*

Name	Country	Name	Country
Compagnie de Saint-Gobain	France	DCC	United Kingdom
Enagás	Spain	Energean	United Kingdom
Eni	Italy	Equinor	Norway
Galp	Portugal	Geberit	Switzerland
Glencore	United Kingdom	Harbour Energy	United Kingdom
Heidelberg Materials	Germany	Holcim	Switzerland
KGHM Polska Miedz	Poland	Kingspan	Ireland
Neste	Finland	Norsk Hydro	Norway
OMV	Austria	Orlen	Poland
Repsol	Spain	Rio Tinto	United Kingdom
Rubis	France	SSAB	Sweden
Shell	United Kingdom	Subsea 7	Norway
Tenaris	Italy	ThyssenKrupp	Germany
TotalEnergies	France	Voestalpine	Austria
Wienerberger	Austria	Wienerberger	Austria
**Financials**			
3i Group PLC	United Kingdom	ABN Amro	Netherlands
ASR	Netherlands	AXA	France
Abrdn	United Kingdom	Ackermanns & Van Haaren	Belgium
Admiral Group	United Kingdom	Aegon	Netherlands
Ageas	Belgium	Allfunds Group	Netherlands
Allianz	Germany	Allied Irish Banks	Ireland
Amundi	France	Assicurazioni Generali	Italy
Avanza	Sweden	Aviva	United Kingdom
Azimut Holding	Italy	BAWAG Group	Austria
BCV	Switzerland	BNP Paribas	France
Banco BPM	Italy	Banco Bilbao Vizcaya	Spain
Banco Santander	Spain	Banco de Sabadell	Spain
Bank Pekao	Poland	Bank of Ireland	Ireland
Bankinter	Spain	Barclays	United Kingdom
Beazley	United Kingdom	Bper Banca	Italy
Bâloise Holding	Switzerland	Caixabank	Spain
Cembra Money Bank	Switzerland	Close Brothers	United Kingdom
Commerzbank	Germany	Credit Agricole	France

Table S1: List of companies by name and country, grouped by SICS sector *(continued)*

**Name**	**Country**	**Name**	**Country**
DNB Bank	Norway	Danske Bank	Denmark
Deutsche Bank	Germany	Deutsche Börse	Germany
Direct Line	United Kingdom	EQT	Sweden
Erste Group	Austria	Eurazeo	France
Euronext	France	Exor	Netherlands
Fineco Bank	Italy	Gjensidige	Norway
Groupe Bruxelles Lambert	Belgium	HSBC	United Kingdom
Hannover Rück	Germany	Hargreaves Lansdown	United Kingdom
Helvetia	Switzerland	Hiscox	United Kingdom
IG Group	United Kingdom	ING Groep	Netherlands
Industrivärden	Sweden	Intermediate Capital Group	United Kingdom
Intesa Sanpaolo	Germany	Investec	United Kingdom
Investor	Sweden	JYSKE Bank	Denmark
Julius Baer	Switzerland	KBC	Belgium
Kinnevik	Sweden	Legal & General	United Kingdom
Lloyds Banking Group	United Kingdom	London Stock Exchange Group	United Kingdom
M&G Investments	United Kingdom	Man Group	United Kingdom
Mediobanca	Italy	Muenchener Rueck	Germany
NN Group	Netherlands	NatWest Group	United Kingdom
Nordea Bank	Sweden	Nordnet	Sweden
Osb Group	United Kingdom	PZU	Poland
Partners Group	Switzerland	Phoenix	United Kingdom
Poste Italiane	Italy	Powszechna Kasa Oszczednosci Bank	Poland
Prudential	United Kingdom	Ringkjobing Landbobank	Denmark
SCOR	France	Sampo Group	Finland
Santander Bank Polska	Poland	Schroders	United Kingdom
Skandinaviska Enskilda Banken	Sweden	Societe Generale	France
Sofina	Belgium	St James’s Place	United Kingdom
Standard Chartered	United Kingdom	Storebrand	Norway
Svenska Handelsbanken	Sweden	Swedbank	Sweden
Swiss Life	Switzerland	Swiss Re Group	Switzerland
Sydbank	Denmark	Talanx	Germany
Topdanmark	Denmark	Tryg	Denmark
UBS	Switzerland	UniCredit	Italy

Table S1: List of companies by name and country, grouped by SICS sector *(continued)*

Name	Country	Name	Country
Virgin Money UK	United Kingdom	Wendel	France
Zurich	Switzerland	Zurich	Switzerland
**Food & Beverage**			
AAK	Sweden	Ahold Delhaize	Netherlands
Anheuser-Busch Inbev	Belgium	Associated British Foods	United Kingdom
Axfood	Sweden	Bakkafrost	Norway
Barry Callebaut	Switzerland	British American Tobacco	United Kingdom
Britvic	United Kingdom	Campari	Italy
Carlsberg	Denmark	Carrefour	France
Coca Cola HBC	Switzerland	Compass Group	United Kingdom
Danone	France	Diageo	United Kingdom
Dino	Poland	Genus	United Kingdom
Glanbia	Ireland	Greggs	United Kingdom
Heineken	Netherlands	Heineken Holding	Netherlands
Imperial Brands	United Kingdom	JDE Peet's	Netherlands
Jerónimo Martins	Portugal	Kerry	Ireland
Kesko	Finland	Lindt & Spruengl	Switzerland
Lotus Bakeries	Belgium	Mowi	Norway
Nestle	Switzerland	Ocado Group	United Kingdom
Orkla	Norway	Pernod Ricard	France
Royal Unibrew	Denmark	Rémy Cointreau	France
SSP Group	United Kingdom	Sainsbury's	United Kingdom
Salmar	Norway	Sodexo	France
Tate & Lyle	United Kingdom	Tesco	United Kingdom
**Health Care**			
ALK-Abelló	Denmark	Alcon	Switzerland
Ambu	Denmark	Amplifon	Italy
Argenx	Netherlands	AstraZeneca	United Kingdom
Bachem	Switzerland	Bavarian Nordic	Denmark
Bayer	Germany	Biomérieux	France
Carl Zeiss Meditec	Germany	Coloplast	Denmark
Convatec	United Kingdom	Dechra	United Kingdom
Demant	Denmark	DiaSorin	Italy

Table S1: List of companies by name and country, grouped by SICS sector *(continued)*

**Name**	**Country**	**Name**	**Country**
Elekta	Sweden	EssilorLuxottica	France
Evotec	Germany	Fresenius	Germany
Fresenius Medical Care	Germany	GN Store Nord	Denmark
Galenica	Switzerland	Genmab	Denmark
Getinge	Sweden	GlaxoSmithKline	United Kingdom
Grifols	Spain	Hikma.	United Kingdom
Indivior	United Kingdom	Ipsen	France
Lifco	Sweden	Lonza	Switzerland
Merck	Germany	Novartis	Switzerland
Novo Nordisk	Denmark	Orion	Finland
Philips	Netherlands	Qiagen	Germany
Recordati	Italy	Roche	Switzerland
Sanofi	France	Sartorius	Germany
Sartorius Stedim Biotech	France	Sectra	Sweden
Siegfried	Switzerland	Siemens Healthineers	Germany
Smith & Nephew	United Kingdom	Sonova	Switzerland
Straumann Holding	Switzerland	Swedish Orphan Biovitrum	Sweden
Tecan	Switzerland	UCB	Belgium
**Infrastructure**			
A2A	Italy	ACS	Spain
Acciona	Spain	Aedifica	Belgium
Allreal	Switzerland	Arcadis	Netherlands
BKW	Switzerland	Balder	Sweden
Balfour Beatty	United Kingdom	Barratt Developments	United Kingdom
Bellway	United Kingdom	Berkeley Group	United Kingdom
Big Yellow Group	United Kingdom	Bouygues	France
British Land	United Kingdom	Castellum	Sweden
Centrica	United Kingdom	Cofinimmo	Belgium
Colonial	Spain	Covivo	France
Derwent London	United Kingdom	Drax	United Kingdom
E.ON	Germany	EDP Energias de Portugal	Portugal
EDP Renovaveis	Portugal	Eiffage	France
Elia	Belgium	Encavis	Germany
Endesa	Spain	Enel	Italy

Table S1: List of companies by name and country, grouped by SICS sector *(continued)*

**Name**	**Country**	**Name**	**Country**
Engie	France	Fabege	Sweden
Ferrovial	Spain	Fortum	Finland
Gaztransport & Technigaz	France	Gecina	France
Hera	Italy	Iberdrola	Spain
Italgas	Italy	Klepierre	France
Kojamo	Finland	LEG Immobilien	Germany
LXI REIT	United Kingdom	Land Securities	United Kingdom
Londonmetric Property PLC	United Kingdom	Lundbergs	Sweden
Merlin Properties REIT	Spain	National Grid	United Kingdom
Naturgy	Spain	Nel	Norway
Orsted	Denmark	Pennon	United Kingdom
Persimmon	United Kingdom	Psp Swiss Property	Switzerland
RWE	Germany	Redeia Corporacion	Spain
SSE	United Kingdom	Safestore	United Kingdom
Sagax	Sweden	Segro	United Kingdom
Severn Trent	United Kingdom	Skanska	Sweden
Snam	Italy	Spie	France
Sweco	Sweden	Swiss Prime Site	Switzerland
Taylor Wimpey	United Kingdom	Technip Energies	France
Terna	Italy	Tritax Big Box	United Kingdom
Unibail-Rodamco-Westfield	France	Unite Group	United Kingdom
United Utilities	United Kingdom	Veolia	France
Verbund	Austria	Vinci	France
Vistry Group	United Kingdom	Vonovia	Germany
Warehouses De Pauw	Belgium	Wihlborgs	Sweden
**Renewable Resources & Alternative Energy**			
Corporacion Acciona Energias Renovables	Spain	Holmen	Sweden
Stora Enso	Finland	Svenska Cellulosa	Sweden
UPM	Finland	Vestas Wind Systems	Denmark
**Resource Transformation**			
ABB	Switzerland	Aalberts	Netherlands
Addtech	Sweden	Airbus	Germany
AkzoNobel	Netherlands	Alfa Laval	Sweden

Table S1: List of companies by name and country, grouped by SICS sector *(continued)*

**Name**	**Country**	**Name**	**Country**
Alstom	France	Andritz	Austria
Arkema	France	Ashtead Group	United Kingdom
Assa Abloy	Sweden	Atlas Copco	Sweden
Azelis Group	Belgium	BAE Systems	United Kingdom
BASF	Germany	Beijer Ref	Sweden
Belimo	Switzerland	Billerud	Sweden
Brenntag	Germany	Bucher	Switzerland
CHR Hansen	Denmark	CNH Industrial	Italy
Clariant	Switzerland	Covestro	Germany
Croda	United Kingdom	DS Smith	United Kingdom
DSM	Netherlands	Daimler Truck Holding	Germany
Dassault Aviation	France	EMS-Chemie	Switzerland
Epiroc	Sweden	Evonik Industries	Germany
Fuchs Petrolub	Germany	GEA Group	Germany
Georg Fischer	Switzerland	Gerresheimer	Germany
Givaudan	Switzerland	Halma	United Kingdom
Hexpol	Sweden	Huhtamaki	Finland
Husqvarna Group	Sweden	IMCD	Netherlands
IMI	United Kingdom	Indutrade	Sweden
Interpump Group	Italy	Johnson Matthey	United Kingdom
K+S	Germany	KONE	Finland
Kion Group	Germany	Knorr-Bremse	Germany
Kongsberg	Norway	L'Air Liquide	France
Lanxess	Germany	Latour	Sweden
Legrand	France	Leonardo	Italy
MTU Aero Engines	Germany	Melrose	United Kingdom
Metso	Finland	Mondi	United Kingdom
NKT A/S	Denmark	Nexans	France
Nibe	Sweden	Novozymes	Denmark
OCI	Netherlands	Prysmian Group	Italy
Qinetiq	United Kingdom	Rational	Germany
Rexel	France	Rheinmetall	Germany
Rolls-Royce Holdings	United Kingdom	Rotork	United Kingdom
SFS Group	Switzerland	SIG	Switzerland

Table S1: List of companies by name and country, grouped by SICS sector *(continued)*

**Name**	**Country**	**Name**	**Country**
SKF	Sweden	Saab	Sweden
Safran	France	Sandvik	Sweden
Schindler	Switzerland	Schneider Electric	France
Siemens	Germany	Siemens Energy	Germany
Signify	Netherlands	Sika Group	Switzerland
Smiths	United Kingdom	Smurfit Kappa Group	Ireland
Solvay	Belgium	Spectris	United Kingdom
Spirax-Sarco Engineering	United Kingdom	Symrise	Germany
Thales	France	Tomra Systems	Norway
Trelleborg	Sweden	Umicore	Belgium
VAT	Switzerland	Valmet	Finland
Verallia	France	Vidrala	Spain
Viscofan	Spain	Volvo	Sweden
Wacker Chemie	Germany	Weir	United Kingdom
Wärtsilä	Finland	Yara	Norway
**Services**			
Accor	France	Adecco Group	Switzerland
Aena	Spain	Alten	France
Aéroports de Paris	France	Bureau Veritas	France
CTS Eventim	Germany	DKSH	Switzerland
Edenred	France	Elis	France
Entain	United Kingdom	Eurofins	France
Evolution	Sweden	Experian	United Kingdom
Flughafen Zürich	Switzerland	Flutter Entertainment	Ireland
Hays	United Kingdom	ISS	Denmark
ITV	United Kingdom	Informa	United Kingdom
Intertek	United Kingdom	Intercontinental Hotels Group	United Kingdom
Kindred	Sweden	La Francaise des Jeux	France
Pearson	United Kingdom	Publicis	France
Randstad	Netherlands	Relx	United Kingdom
Rentokil Initial	United Kingdom	SGS	Switzerland
Securitas	Sweden	TUI	United Kingdom
Universal Music Group	Netherlands	Vivendi	France
WPP	United Kingdom	Whitbread	United Kingdom

Table S1: List of companies by name and country, grouped by SICS sector *(continued)*

**Name**	**Country**	**Name**	**Country**
Wolters Kluwer	Netherlands	Wolters Kluwer	Netherlands
**Technology & Communications**			
AMS Osram	Switzerland	ASM	Netherlands
ASML	Netherlands	Adyen	Netherlands
Aixtron	Germany	Amadeus	Spain
AutoTrader	United Kingdom	BE Semiconductor Industries	Netherlands
BT Group	United Kingdom	Bechtle	Germany
Capgemini	France	Cellnex	Spain
Computacenter	United Kingdom	Dassault Systemes	France
Delivery Hero	Germany	Deutsche Telekom	Germany
Elisa	Finland	Embracer Group	Sweden
Ericsson	Sweden	Fortnox	Sweden
Freenet	Germany	Hexagon	Sweden
Infineon	Germany	Infrastrutture Wireless Italiane	Italy
Just Eat Takeaway.com	Netherlands	Kpn	Netherlands
Lagercrantz Group	Sweden	Logitech	Switzerland
Millicom	Sweden	Nemetschek	Germany
Nexi	Italy	Nokia	Sweden
Nordic Semiconductor	Norway	Orange	France
Prosus	Netherlands	RS Group	United Kingdom
Reply	Italy	Rightmove	United Kingdom
SAP	Germany	SES	France
STMicroelectronics	Netherlands	Sage	United Kingdom
Scout24	Germany	Serco Group	United Kingdom
SimCorp	Denmark	Softcat	United Kingdom
Soitec	France	Sopra Steria	France
Swisscom	Switzerland	TIM (Telecom Italia)	Italy
Tele2	Sweden	Telefónica	Spain
Telefónica Deutschland	Germany	Telenor	Norway
Teleperformance	France	Telia Company	Sweden
Temenos	Switzerland	TietoEVRY	Finland
Ubisoft	France	Vodafone	United Kingdom
Worldline	France	Worldline	France

Table S1: List of companies by name and country, grouped by SICS sector *(continued)*

Name	Country	Name	Country
**Transportation**			
A.P. Moeller-Maersk	Denmark	Air France-KLM	France
BMW	Germany	Bolloré	France
Continental	Germany	DSV	Denmark
Deutsche Lufthansa	Germany	Deutsche Post DHL	Germany
Dowlais Group	United Kingdom	Ferrari	Italy
Forvia SE	France	Frontline	Norway
Getlink	France	InPost	Poland
International Consolidated Airlines	Spain	International Distributions	United Kingdom
Kuehne und Nagel International	Switzerland	Mercedes-Benz Group	Germany
Michelin	France	Porsche	Germany
Porsche Automobil Holding	Germany	Renault	France
Ryanair	Ireland	Stellantis	Netherlands
Valeo	France	Volkswagen	Germany
Volvo Car	Sweden	Volvo Car	Sweden
**No sector classification**			
Allegro	Poland	Wise	United Kingdom

Table S2: Transparency trends by rating group

	(1)	(2)	(3)	(4)
Constant	0.241***	−0.037		
	(0.006)	(0.058)		
Top-10% group	0.051***	0.036**	0.036**	0.006
	(0.013)	(0.013)	(0.012)	(0.012)
Bottom-10% group	−0.047***	−0.046***	−0.037***	−0.011
	(0.011)	(0.011)	(0.010)	(0.008)
Time trend	0.013***	0.013***	0.013***	0.014***
	(0.001)	(0.001)	(0.001)	(0.001)
Top-10% $\times$ Time trend	−0.004*	−0.002	−0.003	−0.003
	(0.002)	(0.002)	(0.002)	(0.002)
Bottom-10% $\times$ Time trend	0.003*	0.004*	0.003	0.002
	(0.002)	(0.002)	(0.002)	(0.001)
Determinant for top/bottom groups		ESG rating		
Controls	No	Yes	Yes	Yes
Sector FE	No	No	Yes	No
Firm FE	No	No	No	Yes
N	4,609	4,346	4,344	4,346
Adj. R^2	0.174	0.186	0.247	0.600

Notes: This table reports firm-year panel regressions where the dependent variable is the company-level transparency score, calculated as the number of reported ESG indicators divided by all indicators listed in ESRS. The specification includes top-10% and bottom-10% dummies of the lagged ESG rating from MSCI, a linear time trend, and interactions between the time trend and the group dummies as regressors. Deciles are calculated on a yearly basis. Model (1) includes no additional controls or fixed effects. Models (2)-(4) additionally include lagged market capitalization and the lagged ESG controversies score from Refinitv as continuous controls. Model (3) includes sector fixed effects. Model (4) includes firm fixed effects. Standard errors are clustered at the firm level and reported in parentheses. Significance levels: ***, **, * correspond to 0.1%, 1%, and 5%, respectively. Reported significance levels are based on two-sided t-tests. No multiple-comparison adjustments were applied. Estimates are based on ordinary least squares (OLS) regression.

Table S3: Inflation-adjusted median revenues in 2023 EUR

Year	Median revenue (in 2023 EUR)
2014	5,157,437,155
2015	5,312,233,674
2016	4,727,198,908
2017	5,024,952,257
2018	5,256,175,489
2019	5,583,455,174
2020	5,126,857,597
2021	5,758,371,581
2022	5,864,532,399
2023	6,019,500,000

Notes: Values are based on USD-denominated reported sales from Worldscope. We convert these values to EUR using the implied annual exchange rates provided by Worldscope [5]. To adjust for inflation, we apply consumer price index adjustment factors from Eurostat, based on the Harmonised Index of Consumer Prices for the EU-19 countries, with 2023 set as the base year [6].

Table S4: Regression analysis for scope 3 emissions

	Extracted values		Refinitiv	
	(1)	(2)	(3)	(4)
Constant	11.761***	10.037***	12.264***	11.648***
	(0.262)	(0.321)	(0.261)	(0.278)
2015	−0.014	0.065	−0.115	−0.114
	(0.256)	(0.257)	(0.117)	(0.116)
2016	0.050	0.092	−0.156	−0.210
	(0.273)	(0.271)	(0.167)	(0.166)
2017	−0.036	−0.002	0.115	0.044
	(0.296)	(0.286)	(0.188)	(0.191)
2018	0.147	0.232	0.354	0.243
	(0.291)	(0.286)	(0.204)	(0.203)
2019	0.137	0.092	0.446	0.300
	(0.295)	(0.294)	(0.236)	(0.235)
2020	0.146	0.049	0.576*	0.305
	(0.286)	(0.279)	(0.245)	(0.250)
2021	0.864**	0.618*	1.010***	0.588*
	(0.278)	(0.274)	(0.243)	(0.256)
2022	1.275***	0.830**	1.490***	0.911***
	(0.276)	(0.274)	(0.244)	(0.266)
2023	1.563***	0.926***	1.437***	0.744**
	(0.274)	(0.277)	(0.256)	(0.280)
Relative scope 3 transparency score		5.133***		3.070***
		(0.567)		(0.520)
Company FE	No	No	No	No
N	3159	3159	3108	3108
Adj. R^2	0.024	0.078	0.026	0.057

Notes: This table reports estimates from a regression analysis where the dependent variable is the scope 3 greenhouse gas emissions (in log values). Models (1) and (2) use the scope 3 emissions data from our machine learning framework, while models (3) and (4) use the corresponding values from Refinitiv data. All regressions include year fixed effects (FEs). Additionally, models (2) and (4) include a relative transparency score for scope 3 emissions, which is a company-level measure of scope 3 emissions transparency calculated as the number of reported indicators divided by all scope 3 emissions indicators. Standard errors are clustered at the company level. Significance levels: ***, **, and * indicate statistical significance at the 0.1%, 1%, and 5% level, respectively. Reported significance levels are based on two-sided t-tests. No multiple-comparison adjustments were applied. Estimates are based on ordinary least squares (OLS) regression. We find a sharp increase in scope 3 emissions from 2021 onward (see models (1) and (2)). The effect gets substantially smaller when we control for relative scope 3 transparency (see models (2) and (4)), indicating that more extensive reporting of individual scope 3 categories explains part of the increase in overall scope 3 emissions. The results are consistent across both data sources (i.e., the emission data extracted by our machine learning framework and the emission data by Refinitiv).

Table S5: Per-indicator standardized mean absolute error (sMAE) and standardized root mean squared error (sRMSE).

Indicator	n	sMAE	sRMSE
Amount of internal and external lobbying expenses	4	—	—
Annual remuneration ratio	3	0.000	0.000
Average number of days to pay invoice from date when contractual or statutory term of payment starts to be calculated	4	0.000	0.000
Average number of training hours per employee and (or) non-employee: Employees	10	0.345	0.621
Energy consumption related to own operations	10	0.045	0.071
Gender pay gap	4	0.323	0.647
Gross Scope 1 greenhouse gas emissions	9	0.000	0.000
Gross Scope 3 greenhouse gas emissions	9	0.000	0.000
Gross Scope 3 greenhouse gas emissions: Category 6 Business travel	8	0.130	0.359
Gross market-based Scope 2 greenhouse gas emissions	10	0.000	0.000
Number of complaints filed through channels for people in own workforce to raise concerns	7	0.390	0.868
Number of days lost to work-related injuries and fatalities from work-related accidents, work-related ill health and fatalities from ill health: Employees	3	28.116	47.564
Percentage of employee turnover	8	0.018	0.036
Percentage of employees at top management level: Female	6	0.843	1.072
Percentage of independent board members	9	0.190	0.501
Percentage of non-recycled waste	3	0.711	1.193
Percentage of renewable sources in total energy consumption	3	0.206	0.260
Percentage of total employees covered by collective bargaining agreements	7	0.007	0.017
Waste generated	7	0.268	0.666
Water consumption	6	0.258	0.631

Notes: For each indicator, we calculated the standardized mean absolute error (sMAE) and standardized root mean squared error (sRMSE) to make performance comparable across indicators with different scales. Standardization was performed by dividing the absolute or squared error by the scale of the manually annotated values for that indicator, using the interquartile range (IQR) as the denominator. This approach yields unit-free metrics that allow performance comparison across indicators regardless of their original scales. Macro-averaged across indicators, the framework achieves an sMAE of 0.21 (95% CI $[0.08, 0.37]$) and sRMSE of 0.39 (95% CI $[0.15, 0.54]$), demonstrating robust alignment with human-annotated values. Indicators marked with "—" had constant values in the manually annotated set (all reported values were zero), resulting in a scale denominator of zero, which makes standardized errors mathematically undefined. These indicators represent a negligible portion of the dataset and were therefore excluded from standardized error summaries to avoid reporting misleading values. In addition, the indicator "Number of days lost to work-related injuries and fatalities from work-related accidents, work-related ill health and fatalities from ill health: Employees" was excluded from the macro-averaged metrics to ensure statistical reliability, as its very small sample size ($n = 3$) combined with a single disproportionate prediction error yielded unstable standardized values. Confidence intervals for aggregated metrics were estimated using a nonparametric percentile bootstrap with 2,000 resamples.

Table S6: Per-indicator disclosure detection and standardization coverage.

Indicator	Disclosure detection rate (%)	Standardization rate (%)
Absolute value of location-based Scope 2 Greenhouse gas emissions reduction	54.5	84.2
Absolute value of market-based Scope 2 Greenhouse gas emissions reduction	35.0	85.0
Absolute value of Scope 1 Greenhouse gas emissions reduction	61.2	73.4
Absolute value of Scope 3 Greenhouse gas emissions reduction	30.1	63.7
Absolute value of total Greenhouse gas emissions reduction	62.1	67.0
Adjusting items to assets at material physical risk, in reconciliation with financial statement	1.0	70.7
Adjusting items to assets at material transition risk, in reconciliation with financial statement	6.8	71.2
Adjusting items to liabilities at material transition risk, in reconciliation with financial statement	12.6	68.4
Adjusting items to net revenue at material physical risk, in reconciliation with financial statement	3.8	75.2
Adjusting items to net revenue at material transition risk, in reconciliation with financial statement	6.9	74.1
Amount of carbon credits outside value chain planned to be cancelled in future	0.4	76.2
Amount of carbon credits outside value chain that are verified against recognised quality standards and cancelled	5.1	77.1
Amount of fines for violation of anti-corruption and anti-bribery laws	20.4	90.4
Amount of fines, penalties, and compensation for damages as result of incidents and complaints	28.2	90.6
Amount of fines, penalties, and compensation for damages for severe human rights incidents connected to own workforce	3.6	84.1
Amount of internal and external lobbying expenses	6.2	88.2
Amount of substances of concern that are generated or used during production or that are procured	2.1	71.1
Amount of substances of concern that leave facilities as emissions	14.8	87.2
Amount of substances of concern that leave facilities as emissions, as products, or as part of products or services	4.7	88.2
Amount of substances of concern that leave facilities as part of products	1.5	63.6
Amount of substances of concern that leave facilities as products	1.8	70.3
Amount of substances of concern that leave facilities as services	0.6	75.8
Amount of substances of very high concern that are generated or used during production or that are procured	1.0	28.6

Indicator	Disclosure detection rate (%)	Standardization rate (%)
Amount of substances of very high concern that leave facilities as emissions	0.6	62.9
Amount of substances of very high concern that leave facilities as emissions, as products, or as part of products or services	0.6	47.2
Amount of substances of very high concern that leave facilities as part of products	0.7	41.5
Amount of substances of very high concern that leave facilities as products	0.8	36.4
Amount of substances of very high concern that leave facilities as services	0.2	54.5
Amount paid for membership to lobbying associations	6.2	88.7
Annual remuneration ratio	55.1	67.0
Area covered by invasive alien species	0.0	100.0
Area of sites owned, leased or managed in or near protected areas or key biodiversity areas that undertaking is negatively affecting	4.9	90.7
Assets	98.5	70.7
Assets at acute material physical risk before considering climate change adaptation actions	1.5	19.3
Assets at chronic material physical risk before considering climate change adaptation actions	1.3	24.7
Assets at material physical risk before considering climate change adaptation actions	1.8	19.6
Assets at material transition risk before considering climate mitigation actions	1.0	66.1
Average number of days to pay invoice from date when contractual or statutory term of payment starts to be calculated	54.1	100.0
Average number of training hours per employee and (or) non-employee: Employees	51.0	100.0
Average number of training hours per employee and (or) non-employee: Non-employees	42.9	100.0
Biogenic emissions of CO2 from combustion or bio-degradation of biomass not included in Scope 1 GHG emissions	5.7	94.5
Biogenic emissions of CO2 from combustion or bio-degradation of biomass not included in Scope 2 GHG emissions	7.2	93.2
Biogenic emissions of CO2 from combustion or bio-degradation of biomass that occur in upstream and downstream value chain not included in Scope 3 GHG emissions	1.8	94.1
Board's gender diversity ratio	93.2	99.8
Capital expenditures (CapEx) in conjunction with major incidents and deposits: ESRS E2 Pollution	19.3	87.1

Indicator	Disclosure detection rate (%)	Standardization rate (%)
Carbon price applied for each metric tonne of greenhouse gas emission: CapEx shadow price	22.3	4.3
Carbon price applied for each metric tonne of greenhouse gas emission: Carbon prices for impairment testing	39.4	2.7
Carbon price applied for each metric tonne of greenhouse gas emission: Internal carbon fee or fund	19.7	6.0
Carbon price applied for each metric tonne of greenhouse gas emission: Other internal carbon pricing scheme type	14.4	2.9
Carbon price applied for each metric tonne of greenhouse gas emission: Research and development investment shadow price	12.9	3.5
Carrying amount of assets at material physical risk	7.0	73.8
Carrying amount of assets at material transition risk	8.9	78.2
Carrying amount of liabilities at material transition risk	20.0	79.5
Carrying amount of real estate assets for which energy consumption is based on internal estimates	13.6	72.3
Consumption of purchased or acquired electricity, heat, steam, and cooling from renewable sources	62.1	58.0
Consumption of purchased or acquired electricity, heat, steam, or cooling from fossil sources	68.4	81.1
Consumption of self-generated non-fuel renewable energy	37.6	84.6
Date of adoption of transition plan for undertaking not having adopted transition plan yet: ESRS E1 Climate change	19.6	94.9
Date of last process modification: ESRS E1 Climate change	88.5	95.0
Date of last process modification: ESRS E2 Pollution	67.6	94.7
Date of last process modification: ESRS E3 Water and marine resources	43.0	94.6
Date of last process modification: ESRS E4 Biodiversity and ecosystems	38.2	89.9
Date of last process modification: ESRS E5 Resource use and circular economy	72.3	92.5
Date of last process modification: ESRS G1 Business conduct	97.2	95.4
Date of last process modification: ESRS S1 Own workforce	90.1	96.1
Date of last process modification: ESRS S2 Workers in value chain	62.1	96.8
Date of last process modification: ESRS S3 Affected communities	61.3	96.5
Date of last process modification: ESRS S4 Consumers and end-users	65.0	97.3
Date when carbon credits outside value chain are planned to be cancelled	1.5	81.0
Dates of future revision of materiality assessment: ESRS E1 Climate change	26.9	39.3
Dates of future revision of materiality assessment: ESRS E2 Pollution	12.9	44.1

Indicator	Disclosure detection rate (%)	Standardization rate (%)
Dates of future revision of materiality assessment: ESRS E3 Water and marine resources	7.0	36.0
Dates of future revision of materiality assessment: ESRS E4 Biodiversity and ecosystems	11.0	29.0
Dates of future revision of materiality assessment: ESRS E5 Resource use and circular economy	12.3	28.8
Dates of future revision of materiality assessment: ESRS G1 Business conduct	34.6	54.3
Dates of future revision of materiality assessment: ESRS S1 Own workforce	23.5	47.7
Dates of future revision of materiality assessment: ESRS S2 Workers in value chain	10.8	32.2
Dates of future revision of materiality assessment: ESRS S3 Affected communities	9.7	35.9
Dates of future revision of materiality assessment: ESRS S4 Consumers and end-users	17.0	43.1
Definition of number of years following reporting period after which deviated long-term time horizon starts	98.8	99.5
Definition of number of years for deviated medium-term time horizon counted from reporting period	98.9	99.9
Duration of classroom training: Administrative / management and supervisory bodies	17.3	93.4
Duration of classroom training: At-risk functions	4.5	90.6
Duration of classroom training: Managers	18.6	92.0
Duration of classroom training: Other own workers	17.2	91.2
Duration of computer-based training: Administrative / management and supervisory bodies	14.2	91.9
Duration of computer-based training: At-risk functions	4.2	90.1
Duration of computer-based training: Managers	14.3	91.5
Duration of computer-based training: Other own workers	14.5	90.6
Duration of voluntary computer-based training: Administrative / management and supervisory bodies	15.5	92.1
Duration of voluntary computer-based training: At-risk functions	3.7	88.1
Duration of voluntary computer-based training: Managers	15.4	93.0
Duration of voluntary computer-based training: Other own workers	13.6	91.1
Emissions to air	56.7	91.4
Emissions to soil	0.4	58.3
Emissions to water	12.9	44.5

Indicator	Disclosure detection rate (%)	Standardization rate (%)
Energy consumption from fossil sources	48.7	86.4
Energy consumption from nuclear sources	5.8	67.8
Energy consumption from renewable sources	61.7	61.2
Energy consumption related to own operations	73.7	84.0
Energy intensity from activities in high climate impact sectors (total energy consumption per net revenue)	32.7	60.9
Estimated amount of potentially stranded assets	3.2	87.0
Expected cost savings from climate change adaptation actions	9.3	84.2
Expected cost savings from climate change mitigation actions	17.3	81.5
Financial and in-kind political contributions made	27.0	90.9
Financial political contributions made	22.4	92.7
Financial resources allocated to action plan (CapEx): ESRS E1 Climate change	49.4	88.3
Financial resources allocated to action plan (OpEx): ESRS E1 Climate change	20.8	81.2
Fuel consumption from coal and coal products	9.8	72.6
Fuel consumption from crude oil and petroleum products	14.9	64.6
Fuel consumption from natural gas	41.7	74.9
Fuel consumption from other fossil sources	27.6	71.5
Fuel consumption from renewable sources	41.7	60.2
Gender pay gap	35.0	94.9
GHG emissions intensity, location-based (total GHG emissions per net revenue)	53.2	57.6
GHG emissions intensity, market-based (total GHG emissions per net revenue)	50.3	57.8
Gross greenhouse gas emissions	80.2	94.5
Gross location-based Scope 2 greenhouse gas emissions	78.3	94.2
Gross location-based Scope 2 greenhouse gas emissions: CapEx shadow price / Approximate value of GHG emissions covered by internal carbon pricing scheme	7.7	31.1
Gross location-based Scope 2 greenhouse gas emissions: Carbon prices for impairment testing / Approximate value of GHG emissions covered by internal carbon pricing scheme	23.7	89.4
Gross location-based Scope 2 greenhouse gas emissions: Consolidated accounting group (financial control)	76.7	94.3
Gross location-based Scope 2 greenhouse gas emissions: Downstream value chain	8.2	93.6
Gross location-based Scope 2 greenhouse gas emissions: Internal carbon fee or fund / Approximate value of GHG emissions covered by internal carbon pricing scheme	20.8	88.6

Indicator	Disclosure detection rate (%)	Standardization rate (%)
Gross location-based Scope 2 greenhouse gas emissions: Other internal carbon pricing scheme type / Approximate value of GHG emissions covered by internal carbon pricing scheme	9.4	88.9
Gross location-based Scope 2 greenhouse gas emissions: Other investees excluded from consolidated accounting group / including contractual arrangements (operational control)	42.4	95.2
Gross location-based Scope 2 greenhouse gas emissions: Own operations	78.5	93.9
Gross location-based Scope 2 greenhouse gas emissions: Research and development investment shadow price / Approximate value of GHG emissions covered by internal carbon pricing scheme	15.0	80.1
Gross location-based Scope 2 greenhouse gas emissions: Transport	18.6	93.5
Gross location-based Scope 2 greenhouse gas emissions: Upstream value chain	18.8	94.4
Gross market-based Scope 2 greenhouse gas emissions	67.5	95.2
Gross market-based Scope 2 greenhouse gas emissions: CapEx shadow price / Approximate value of GHG emissions covered by internal carbon pricing scheme	8.1	20.9
Gross market-based Scope 2 greenhouse gas emissions: Carbon prices for impairment testing / Approximate value of GHG emissions covered by internal carbon pricing scheme	19.7	76.0
Gross market-based Scope 2 greenhouse gas emissions: Consolidated accounting group (financial control)	68.0	95.2
Gross market-based Scope 2 greenhouse gas emissions: Downstream value chain	4.1	92.4
Gross market-based Scope 2 greenhouse gas emissions: Internal carbon fee or fund / Approximate value of GHG emissions covered by internal carbon pricing scheme	18.9	78.4
Gross market-based Scope 2 greenhouse gas emissions: Other internal carbon pricing scheme type / Approximate value of GHG emissions covered by internal carbon pricing scheme	8.9	85.7
Gross market-based Scope 2 greenhouse gas emissions: Other investees excluded from consolidated accounting group / including contractual arrangements (operational control)	19.3	95.4
Gross market-based Scope 2 greenhouse gas emissions: Own operations	71.4	95.0
Gross market-based Scope 2 greenhouse gas emissions: Research and development investment shadow price / Approximate value of GHG emissions covered by internal carbon pricing scheme	10.9	62.1
Gross market-based Scope 2 greenhouse gas emissions: Transport	7.9	94.7

Indicator	Disclosure detection rate (%)	Standardization rate (%)
Gross market-based Scope 2 greenhouse gas emissions: Upstream value chain	13.5	95.1
Gross Scope 1 greenhouse gas emissions	80.8	95.3
Gross Scope 1 greenhouse gas emissions: CapEx shadow price / Approximate value of GHG emissions covered by internal carbon pricing scheme	8.6	16.4
Gross Scope 1 greenhouse gas emissions: Carbon prices for impairment testing / Approximate value of GHG emissions covered by internal carbon pricing scheme	25.6	87.0
Gross Scope 1 greenhouse gas emissions: Consolidated accounting group (financial control)	77.6	94.9
Gross Scope 1 greenhouse gas emissions: Downstream value chain	2.3	85.7
Gross Scope 1 greenhouse gas emissions: Internal carbon fee or fund / Approximate value of GHG emissions covered by internal carbon pricing scheme	24.7	84.6
Gross Scope 1 greenhouse gas emissions: Other internal carbon pricing scheme type / Approximate value of GHG emissions covered by internal carbon pricing scheme	13.4	88.8
Gross Scope 1 greenhouse gas emissions: Other investees excluded from consolidated accounting group / including contractual arrangements (operational control)	28.8	95.9
Gross Scope 1 greenhouse gas emissions: Own operations	80.3	94.8
Gross Scope 1 greenhouse gas emissions: Research and development investment shadow price / Approximate value of GHG emissions covered by internal carbon pricing scheme	13.7	65.7
Gross Scope 1 greenhouse gas emissions: Transport	47.7	94.1
Gross Scope 1 greenhouse gas emissions: Upstream value chain	9.1	91.4
Gross Scope 3 greenhouse gas emissions	57.3	96.0
Gross Scope 3 greenhouse gas emissions: CapEx shadow price / Approximate value of GHG emissions covered by internal carbon pricing scheme	7.3	18.5
Gross Scope 3 greenhouse gas emissions: Carbon prices for impairment testing / Approximate value of GHG emissions covered by internal carbon pricing scheme	22.6	88.5
Gross Scope 3 greenhouse gas emissions: Category 1 Purchased goods and services	25.7	95.4
Gross Scope 3 greenhouse gas emissions: Category 1.1 Cloud computing and data centre services	1.0	87.7
Gross Scope 3 greenhouse gas emissions: Category 10 Processing of sold products	9.6	93.7
Gross Scope 3 greenhouse gas emissions: Category 11 Use of sold products	17.9	94.6

Indicator	Disclosure detection rate (%)	Standardization rate (%)
Gross Scope 3 greenhouse gas emissions: Category 12 End-of-life treatment of sold products	10.3	91.9
Gross Scope 3 greenhouse gas emissions: Category 13 Downstream leased assets	6.1	91.1
Gross Scope 3 greenhouse gas emissions: Category 14 Franchises	2.6	92.1
Gross Scope 3 greenhouse gas emissions: Category 15 Investments	9.3	92.2
Gross Scope 3 greenhouse gas emissions: Category 2 Capital goods	12.8	93.6
Gross Scope 3 greenhouse gas emissions: Category 3 Fuel and energy-related activities	29.6	93.6
Gross Scope 3 greenhouse gas emissions: Category 4 Upstream transportation and distribution	20.2	93.5
Gross Scope 3 greenhouse gas emissions: Category 5 Waste generated in operations	27.4	94.5
Gross Scope 3 greenhouse gas emissions: Category 6 Business travel	43.7	92.6
Gross Scope 3 greenhouse gas emissions: Category 7 Employee commuting	20.0	90.5
Gross Scope 3 greenhouse gas emissions: Category 8 Upstream leased assets	6.5	93.3
Gross Scope 3 greenhouse gas emissions: Category 9 Downstream transportation and distribution	14.3	92.0
Gross Scope 3 greenhouse gas emissions: Downstream value chain	29.2	93.9
Gross Scope 3 greenhouse gas emissions: Internal carbon fee or fund / Approximate value of GHG emissions covered by internal carbon pricing scheme	17.9	86.1
Gross Scope 3 greenhouse gas emissions: Other internal carbon pricing scheme type / Approximate value of GHG emissions covered by internal carbon pricing scheme	11.7	91.0
Gross Scope 3 greenhouse gas emissions: Other Scope 3 greenhouse gas emissions category	59.3	94.6
Gross Scope 3 greenhouse gas emissions: Own operations	45.6	95.2
Gross Scope 3 greenhouse gas emissions: Research and development investment shadow price / Approximate value of GHG emissions covered by internal carbon pricing scheme	9.9	65.7
Gross Scope 3 greenhouse gas emissions: Transport	48.0	93.6
Gross Scope 3 greenhouse gas emissions: Upstream value chain	37.6	95.6
Hazardous waste	42.1	87.9
In-kind political contributions made	4.5	83.3
Increase (decrease) in net revenue from low-carbon products and services or adaptation solutions to which undertaking has or may have access	3.2	41.0

Indicator	Disclosure detection rate (%)	Standardization rate (%)
Increase (decrease) in water storage	1.6	47.8
Liabilities	97.6	67.3
Liabilities from material transition risks that may have to be recognised in financial statements	2.9	87.6
Location-based greenhouse gas emissions	76.6	92.3
Location-based greenhouse gas emissions: CapEx shadow price / Approximate value of GHG emissions covered by internal carbon pricing scheme	8.5	2.6
Location-based greenhouse gas emissions: Carbon prices for impairment testing / Approximate value of GHG emissions covered by internal carbon pricing scheme	13.7	35.4
Location-based greenhouse gas emissions: Downstream value chain	27.7	91.0
Location-based greenhouse gas emissions: Internal carbon fee or fund / Approximate value of GHG emissions covered by internal carbon pricing scheme	15.0	55.8
Location-based greenhouse gas emissions: Other internal carbon pricing scheme type / Approximate value of GHG emissions covered by internal carbon pricing scheme	9.4	60.3
Location-based greenhouse gas emissions: Own operations	78.2	90.7
Location-based greenhouse gas emissions: Research and development investment shadow price / Approximate value of GHG emissions covered by internal carbon pricing scheme	9.1	23.4
Location-based greenhouse gas emissions: Transport	43.0	86.7
Location-based greenhouse gas emissions: Upstream value chain	34.6	91.6
Market-based greenhouse gas emissions	49.4	93.4
Market-based greenhouse gas emissions: CapEx shadow price / Approximate value of GHG emissions covered by internal carbon pricing scheme	10.0	2.1
Market-based greenhouse gas emissions: Carbon prices for impairment testing / Approximate value of GHG emissions covered by internal carbon pricing scheme	17.7	14.2
Market-based greenhouse gas emissions: Downstream value chain	19.0	92.6
Market-based greenhouse gas emissions: Internal carbon fee or fund / Approximate value of GHG emissions covered by internal carbon pricing scheme	15.6	40.3
Market-based greenhouse gas emissions: Other internal carbon pricing scheme type / Approximate value of GHG emissions covered by internal carbon pricing scheme	12.7	50.8
Market-based greenhouse gas emissions: Own operations	72.1	91.9

Indicator	Disclosure detection rate (%)	Standardization rate (%)
Market-based greenhouse gas emissions: Research and development investment shadow price / Approximate value of GHG emissions covered by internal carbon pricing scheme	9.1	9.5
Market-based greenhouse gas emissions: Transport	29.2	89.5
Market-based greenhouse gas emissions: Upstream value chain	26.2	93.9
Microplastics generated	0.0	100.0
Microplastics generated or used	0.1	40.0
Microplastics used	0.1	25.0
Monetised gross Scope 1 and 2 GHG emissions	1.6	70.0
Monetised total GHG emissions	1.9	75.0
Nature-oriented area off site	10.2	73.1
Nature-oriented area on site	15.8	69.4
Net revenue at material physical risk	7.1	81.5
Net revenue at material transition risk	12.0	84.4
Net revenue other than used to calculate GHG intensity	73.2	82.8
Net revenue used to calculate GHG intensity	57.8	89.3
Non-recycled waste	53.4	80.2
Non-renewable energy production	17.4	79.0
Number of cases of recordable work-related ill health: Employees	20.9	100.0
Number of cases of recordable work-related ill health: Non-employees	4.0	100.0
Number of complaints filed through channels for people in own workforce to raise concerns	43.0	100.0
Number of complaints filed to National Contact Points for OECD Multinational Enterprises	1.1	100.0
Number of complaints received from consumers and end-users: ESRS S4 Consumers and end-users	22.2	100.0
Number of confirmed incidents in which own workers were dismissed or disciplined for corruption or bribery-related incidents	28.0	100.0
Number of confirmed incidents of corruption or bribery	39.4	100.0
Number of confirmed incidents relating to contracts with business partners that were terminated or not renewed due to violations related to corruption or bribery	8.8	100.0
Number of convictions for violation of anti-corruption and anti-bribery laws	6.6	100.0
Number of days lost to work-related injuries and fatalities from work-related accidents, work-related ill health and fatalities from ill health: Employees	26.9	100.0
Number of days lost to work-related injuries and fatalities from work-related accidents, work-related ill health and fatalities from ill health: Non-employees	3.8	100.0

Indicator	Disclosure detection rate (%)	Standardization rate (%)
Number of emission allowances stored (from previous allowances) at beginning of reporting period	4.1	100.0
Number of employees (head count or full-time equivalent), at end of period: Female / Full-time employees	91.8	100.0
Number of employees (head count or full-time equivalent), at end of period: Female / Non-guaranteed hours employees	79.5	100.0
Number of employees (head count or full-time equivalent), at end of period: Female / Part-time employees	82.4	100.0
Number of employees (head count or full-time equivalent), at end of period: Female / Permanent employees	85.9	100.0
Number of employees (head count or full-time equivalent), at end of period: Female / Temporary employees	74.8	100.0
Number of employees (head count or full-time equivalent), at end of period: Gender not reported / Full-time employees	99.0	100.0
Number of employees (head count or full-time equivalent), at end of period: Gender not reported / Non-guaranteed hours employees	98.1	100.0
Number of employees (head count or full-time equivalent), at end of period: Gender not reported / Part-time employees	99.2	100.0
Number of employees (head count or full-time equivalent), at end of period: Gender not reported / Permanent employees	99.0	100.0
Number of employees (head count or full-time equivalent), at end of period: Gender not reported / Temporary employees	99.1	100.0
Number of employees (head count or full-time equivalent), at end of period: Gender other than female and male / Full-time employees	56.1	100.0
Number of employees (head count or full-time equivalent), at end of period: Gender other than female and male / Non-guaranteed hours employees	14.8	100.0
Number of employees (head count or full-time equivalent), at end of period: Gender other than female and male / Part-time employees	56.6	100.0
Number of employees (head count or full-time equivalent), at end of period: Gender other than female and male / Permanent employees	72.6	100.0
Number of employees (head count or full-time equivalent), at end of period: Gender other than female and male / Temporary employees	54.6	100.0
Number of employees (head count or full-time equivalent), at end of period: Male / Full-time employees	97.5	100.0
Number of employees (head count or full-time equivalent), at end of period: Male / Non-guaranteed hours employees	81.7	100.0

Indicator	Disclosure detection rate (%)	Standardization rate (%)
Number of employees (head count or full-time equivalent), at end of period: Male / Part-time employees	69.8	100.0
Number of employees (head count or full-time equivalent), at end of period: Male / Permanent employees	90.4	100.0
Number of employees (head count or full-time equivalent), at end of period: Male / Temporary employees	62.8	100.0
Number of employees (head count or full-time equivalent), during period: Female / Full-time employees	92.3	100.0
Number of employees (head count or full-time equivalent), during period: Female / Non-guaranteed hours employees	76.7	100.0
Number of employees (head count or full-time equivalent), during period: Female / Part-time employees	82.0	100.0
Number of employees (head count or full-time equivalent), during period: Female / Permanent employees	85.7	100.0
Number of employees (head count or full-time equivalent), during period: Female / Temporary employees	71.0	100.0
Number of employees (head count or full-time equivalent), during period: Gender not reported / Full-time employees	99.0	100.0
Number of employees (head count or full-time equivalent), during period: Gender not reported / Non-guaranteed hours employees	97.5	100.0
Number of employees (head count or full-time equivalent), during period: Gender not reported / Part-time employees	99.1	100.0
Number of employees (head count or full-time equivalent), during period: Gender not reported / Permanent employees	99.1	100.0
Number of employees (head count or full-time equivalent), during period: Gender not reported / Temporary employees	98.9	100.0
Number of employees (head count or full-time equivalent), during period: Gender other than female and male / Full-time employees	38.4	100.0
Number of employees (head count or full-time equivalent), during period: Gender other than female and male / Non-guaranteed hours employees	6.5	100.0
Number of employees (head count or full-time equivalent), during period: Gender other than female and male / Part-time employees	42.5	100.0
Number of employees (head count or full-time equivalent), during period: Gender other than female and male / Permanent employees	52.4	100.0
Number of employees (head count or full-time equivalent), during period: Gender other than female and male / Temporary employees	41.3	100.0

Indicator	Disclosure detection rate (%)	Standardization rate (%)
Number of employees (head count or full-time equivalent), during period: Male / Full-time employees	97.8	100.0
Number of employees (head count or full-time equivalent), during period: Male / Non-guaranteed hours employees	76.4	100.0
Number of employees (head count or full-time equivalent), during period: Male / Part-time employees	68.0	100.0
Number of employees (head count or full-time equivalent), during period: Male / Permanent employees	90.1	100.0
Number of employees (head count or full-time equivalent), during period: Male / Temporary employees	59.3	100.0
Number of employees (head count) at top management level, at end of period: Female	78.6	100.0
Number of employees (head count) at top management level, at end of period: Gender not reported	80.2	100.0
Number of employees (head count) at top management level, at end of period: Gender other than female and male	13.0	100.0
Number of employees (head count) at top management level, at end of period: Male	75.0	100.0
Number of employees (head count) at top management level, during period: Female	68.9	100.0
Number of employees (head count) at top management level, during period: Gender not reported	82.7	100.0
Number of employees (head count) at top management level, during period: Gender other than female and male	6.1	100.0
Number of employees (head count) at top management level, during period: Male	73.4	100.0
Number of employees (head count) between 30 and 50 years old, at end of period	39.1	100.0
Number of employees (head count) between 30 and 50 years old, during period	36.9	100.0
Number of employees (head count) over 50 years old, at end of period	40.9	100.0
Number of employees (head count) over 50 years old, during period	38.1	100.0
Number of employees (head count) under 30 years old, at end of period	44.4	100.0
Number of employees (head count) under 30 years old, during period	43.1	100.0
Number of employees (head count), at end of period	98.8	100.0
Number of employees (head count), at end of period: Female	86.6	100.0
Number of employees (head count), at end of period: Gender not reported	99.0	100.0

Indicator	Disclosure detection rate (%)	Standardization rate (%)
Number of employees (head count), at end of period: Gender other than female and male	24.5	100.0
Number of employees (head count), at end of period: Male	80.0	100.0
Number of employees (head count), during period	99.0	100.0
Number of employees (head count), during period: Administrative / management and supervisory bodies	97.5	100.0
Number of employees (head count), during period: At-risk functions	76.2	100.0
Number of employees (head count), during period: Female	86.9	100.0
Number of employees (head count), during period: Gender not reported	99.1	100.0
Number of employees (head count), during period: Gender other than female and male	10.1	100.0
Number of employees (head count), during period: Male	82.3	100.0
Number of employees (head count), during period: Managers	95.3	100.0
Number of employees (head count), during period: Other own workers	94.2	100.0
Number of employees in countries with 50 or more employees representing at least 10% of total number of employees, at end of period	77.4	100.0
Number of employees in countries with 50 or more employees representing at least 10% of total number of employees, during period	71.6	100.0
Number of employees receiving anti-corruption and anti-bribery training, during period: Administrative / management and supervisory bodies	28.4	100.0
Number of employees receiving anti-corruption and anti-bribery training, during period: At-risk functions	28.2	100.0
Number of employees receiving anti-corruption and anti-bribery training, during period: Managers	28.3	100.0
Number of employees receiving anti-corruption and anti-bribery training, during period: Other own workers	29.8	100.0
Number of employees who have left undertaking, during period	53.7	100.0
Number of executive members	98.8	100.0
Number of fatalities as result of work-related ill health of other workers working on undertaking's sites: Employees	26.9	100.0
Number of fatalities as result of work-related ill health of other workers working on undertaking's sites: Non-employees	29.5	100.0
Number of fatalities as result of work-related injuries and work-related ill health of other workers working on undertaking's sites: Employees	47.3	100.0
Number of fatalities as result of work-related injuries and work-related ill health of other workers working on undertaking's sites: Non-employees	44.2	100.0

Indicator	Disclosure detection rate (%)	Standardization rate (%)
Number of fatalities as result of work-related injuries of other workers working on undertaking's sites: Employees	45.8	100.0
Number of fatalities as result of work-related injuries of other workers working on undertaking's sites: Non-employees	44.8	100.0
Number of fatalities in own workforce as result of work-related ill health: Employees	42.0	100.0
Number of fatalities in own workforce as result of work-related ill health: Non-employees	28.7	100.0
Number of fatalities in own workforce as result of work-related injuries and work-related ill health: Employees	49.4	100.0
Number of fatalities in own workforce as result of work-related injuries and work-related ill health: Non-employees	46.0	100.0
Number of fatalities in own workforce as result of work-related injuries: Employees	49.7	100.0
Number of fatalities in own workforce as result of work-related injuries: Non-employees	45.4	100.0
Number of incidents of discrimination	26.9	100.0
Number of invasive alien species	0.3	100.0
Number of legal proceedings outstanding for late payments	3.8	100.0
Number of non-employees in own workforce - people provided by undertakings primarily engaged in employment activities, at end of period	14.6	100.0
Number of non-employees in own workforce - people provided by undertakings primarily engaged in employment activities, during period	12.0	100.0
Number of non-employees in own workforce - self-employed people, at end of period	6.2	100.0
Number of non-employees in own workforce - self-employed people, during period	4.6	100.0
Number of non-employees in own workforce, at end of period	29.3	100.0
Number of non-employees in own workforce, during period	20.9	100.0
Number of non-executive members	96.7	100.0
Number of recordable work-related accidents: Employees	64.5	100.0
Number of recordable work-related accidents: Non-employees	16.9	100.0
Number of Scope 1 GHG emission allowances within regulated emission trading schemes	5.9	100.0
Number of severe human rights incidents connected to own workforce	6.2	100.0

Indicator	Disclosure detection rate (%)	Standardization rate (%)
Number of severe human rights incidents connected to own workforce that are non-respect of UN Guiding Principles on Business and Human Rights, ILO Declaration on Fundamental Principles and Rights at Work or OECD Guidelines for Multinational Enterprises	9.7	100.0
Number of severe human rights incidents where undertaking played role securing remedy for those affected	0.4	100.0
Number of sites owned, leased or managed in or near protected areas or key biodiversity areas that undertaking is negatively affecting	5.6	100.0
Operating expenditures (OpEx) in conjunction with major incidents and deposits: ESRS E2 Pollution	8.5	83.4
Percentage of approximate gross Scope 1 greenhouse gas emissions covered by internal carbon pricing scheme: CapEx shadow price	2.6	95.9
Percentage of approximate gross Scope 1 greenhouse gas emissions covered by internal carbon pricing scheme: Carbon prices for impairment testing	12.0	97.5
Percentage of approximate gross Scope 1 greenhouse gas emissions covered by internal carbon pricing scheme: Internal carbon fee or fund	6.7	97.4
Percentage of approximate gross Scope 1 greenhouse gas emissions covered by internal carbon pricing scheme: Other internal carbon pricing scheme type	3.7	98.6
Percentage of approximate gross Scope 1 greenhouse gas emissions covered by internal carbon pricing scheme: Research and development investment shadow price	3.6	90.7
Percentage of approximate gross Scope 2 greenhouse gas emissions covered by internal carbon pricing scheme: CapEx shadow price	2.0	94.0
Percentage of approximate gross Scope 2 greenhouse gas emissions covered by internal carbon pricing scheme: Carbon prices for impairment testing	11.1	97.6
Percentage of approximate gross Scope 2 greenhouse gas emissions covered by internal carbon pricing scheme: Internal carbon fee or fund	6.2	97.8
Percentage of approximate gross Scope 2 greenhouse gas emissions covered by internal carbon pricing scheme: Other internal carbon pricing scheme type	2.7	99.4
Percentage of approximate gross Scope 2 greenhouse gas emissions covered by internal carbon pricing scheme: Research and development investment shadow price	2.1	89.3
Percentage of approximate gross Scope 3 greenhouse gas emissions covered by internal carbon pricing scheme: CapEx shadow price	1.4	98.8
Percentage of approximate gross Scope 3 greenhouse gas emissions covered by internal carbon pricing scheme: Carbon prices for impairment testing	6.3	97.5

Indicator	Disclosure detection rate (%)	Standardization rate (%)
Percentage of approximate gross Scope 3 greenhouse gas emissions covered by internal carbon pricing scheme: Internal carbon fee or fund	4.5	98.5
Percentage of approximate gross Scope 3 greenhouse gas emissions covered by internal carbon pricing scheme: Other internal carbon pricing scheme type	1.4	97.5
Percentage of approximate gross Scope 3 greenhouse gas emissions covered by internal carbon pricing scheme: Research and development investment shadow price	1.3	83.8
Percentage of assets at material physical risk addressed by climate change adaptation actions	1.2	100.0
Percentage of assets at material physical risk before considering climate change adaptation actions	1.6	98.9
Percentage of assets at material transition risk addressed by climate change mitigation actions	0.9	100.0
Percentage of assets at material transition risk before considering climate mitigation actions	1.1	98.5
Percentage of biological materials used to manufacture products and services that are sustainably sourced	8.9	100.0
Percentage of carbon credits cancelled from projects in European Union	0.1	100.0
Percentage of carbon credits cancelled from reduction projects	0.5	93.3
Percentage of carbon credits cancelled from removal projects	0.3	100.0
Percentage of carbon credits cancelled that qualifies as corresponding adjustment	0.1	100.0
Percentage of contractual instruments used for sale and purchase of energy bundled with attributes about energy generation in relation to Scope 2 GHG emissions	1.3	100.0
Percentage of contractual instruments used for sale and purchase of unbundled energy attribute claims in relation to Scope 2 GHG emissions	0.2	100.0
Percentage of contractual instruments, Scope 2 GHG emissions	1.8	85.7
Percentage of data obtained from best estimates (water consumption)	5.6	99.4
Percentage of data obtained from direct measurement (water consumption)	15.2	99.1
Percentage of data obtained from sampling and extrapolation (water consumption)	8.7	99.4
Percentage of employee turnover	64.9	99.9
Percentage of employees and (or) non-employees paid below applicable adequate wage benchmark: Employees	2.6	100.0
Percentage of employees and (or) non-employees paid below applicable adequate wage benchmark: Non-employees	0.9	100.0

Indicator	Disclosure detection rate (%)	Standardization rate (%)
Percentage of employees and (or) non-employees that participated in regular performance and career development reviews: Employees	50.7	100.0
Percentage of employees and (or) non-employees that participated in regular performance and career development reviews: Non-employees	2.5	100.0
Percentage of employees at top management level: Female	90.0	100.0
Percentage of employees at top management level: Gender not reported	44.3	100.0
Percentage of employees at top management level: Gender other than female and male	3.2	100.0
Percentage of employees at top management level: Male	80.7	100.0
Percentage of employees between 30 and 50 years old	47.9	100.0
Percentage of employees entitled to take family-related leaves	9.5	100.0
Percentage of employees over 50 years old	49.2	100.0
Percentage of employees under 30 years old	50.4	100.0
Percentage of energy consumption from nuclear sources in total energy consumption	7.2	99.8
Percentage of entitled employees that took family-related leave	9.4	98.9
Percentage of entitled employees that took family-related leave: Female	17.4	98.5
Percentage of entitled employees that took family-related leave: Gender not reported	16.1	98.6
Percentage of entitled employees that took family-related leave: Gender other than female and male	0.6	100.0
Percentage of entitled employees that took family-related leave: Male	15.1	98.3
Percentage of estimated share of potentially stranded assets of total assets at material transition risk	1.2	100.0
Percentage of fossil sources in total energy consumption	44.0	100.0
Percentage of functions-at-risk covered by anti-corruption and anti-bribery training programmes	18.8	100.0
Percentage of Gross Scope 3 greenhouse gas emissions calculated using primary data obtained from suppliers or other value chain partners	6.7	100.0
Percentage of independent board members	90.2	100.0
Percentage of location-based Scope 2 Greenhouse gas emissions reduction (as of emissions of base year)	52.5	99.7
Percentage of market-based Scope 2 GHG emissions linked to purchased electricity bundled with instruments	11.3	98.5
Percentage of market-based Scope 2 Greenhouse gas emissions reduction (as of emissions of base year)	37.4	99.8

Indicator	Disclosure detection rate (%)	Standardization rate (%)
Percentage of members of administrative, management and supervisory bodies: Female	94.5	100.0
Percentage of members of administrative, management and supervisory bodies: Gender not reported	26.0	99.9
Percentage of members of administrative, management and supervisory bodies: Gender other than female and male	7.8	100.0
Percentage of members of administrative, management and supervisory bodies: Male	92.9	100.0
Percentage of net revenue from business activities at material physical risk	0.8	100.0
Percentage of net revenue from business activities at material transition risk	1.9	100.0
Percentage of net revenue from customers operating in coal-related activities	1.2	100.0
Percentage of net revenue from customers operating in gas-related activities	3.3	100.0
Percentage of net revenue from customers operating in oil-related activities	3.4	100.0
Percentage of net revenue made with products and services that are or that contain substances of concern: ESRS E2 Pollution	1.2	100.0
Percentage of net revenue made with products and services that are or that contain substances of very high concern: ESRS E2 Pollution	1.2	100.0
Percentage of non-employees in own workforce whose working conditions and terms of employment are determined or influenced by collective bargaining agreements	17.9	100.0
Percentage of non-recycled waste	50.8	98.7
Percentage of own workers covered by health and safety management system based on legal requirements and (or) recognised standards or guidelines and which has been internally audited and (or) audited or certified by external party	43.4	100.0
Percentage of payments aligned with standard payment terms	13.2	97.1
Percentage of people in own workforce who are covered by health and safety management system based on legal requirements and (or) recognised standards or guidelines: Employees	67.0	100.0
Percentage of people in own workforce who are covered by health and safety management system based on legal requirements and (or) recognised standards or guidelines: Non-employees	22.1	100.0
Percentage of persons with disabilities amongst employees	23.4	99.9
Percentage of persons with disabilities amongst employees: Female	13.0	100.0
Percentage of persons with disabilities amongst employees: Gender not reported	27.3	100.0
Percentage of persons with disabilities amongst employees: Gender other than female and male	1.8	100.0

Indicator	Disclosure detection rate (%)	Standardization rate (%)
Percentage of persons with disabilities amongst employees: Male	11.0	100.0
Percentage of remuneration recognised in current period that is linked to climate related considerations: ESRS E1 Climate change	17.4	100.0
Percentage of renewable sources in total energy consumption	60.9	99.8
Percentage of Scope 1 greenhouse gas emissions from regulated emission trading schemes	4.5	98.5
Percentage of Scope 1 greenhouse gas emissions from regulated emission trading schemes: Consolidated accounting group (financial control)	4.8	97.1
Percentage of Scope 1 greenhouse gas emissions from regulated emission trading schemes: Other investees excluded from consolidated accounting group / including contractual arrangements (operational control)	2.9	99.4
Percentage of Scope 1 Greenhouse gas emissions reduction (as of emissions of base year)	61.2	99.9
Percentage of Scope 1 Greenhouse gas emissions reduction in total Greenhouse gas emissions reduction	28.5	99.8
Percentage of Scope 2 location-based Greenhouse gas emissions reduction in total Greenhouse gas emissions reduction	20.6	99.7
Percentage of Scope 2 market-based Greenhouse gas emissions reduction in total Greenhouse gas emissions reduction	16.6	99.7
Percentage of Scope 3 Greenhouse gas emissions reduction (as of emissions of base year)	34.2	99.9
Percentage of Scope 3 Greenhouse gas emissions reduction in total Greenhouse gas emissions reduction	18.9	99.8
Percentage of secondary reused or recycled components, secondary intermediary products and secondary materials used to manufacture products and services	7.4	98.8
Percentage of target related to location-based Scope 2 Greenhouse gas emissions	7.0	98.8
Percentage of target related to location-based Scope 2 Greenhouse gas emissions (as of emissions of base year)	31.3	97.9
Percentage of target related to market-based Scope 2 Greenhouse gas emissions	7.7	96.6
Percentage of target related to market-based Scope 2 Greenhouse gas emissions (as of emissions of base year)	23.8	97.4
Percentage of target related to Scope 1 Greenhouse gas emissions	36.3	99.7
Percentage of target related to Scope 1 Greenhouse gas emissions (as of emissions of base year)	55.1	99.1
Percentage of target related to Scope 3 Greenhouse gas emissions	21.9	99.8

Indicator	Disclosure detection rate (%)	Standardization rate (%)
Percentage of target related to Scope 3 Greenhouse gas emissions (as of emissions of base year)	32.7	99.2
Percentage of target related to total Greenhouse gas emissions	45.0	99.3
Percentage of target related to total Greenhouse gas emissions (as of emissions of base year)	65.2	99.4
Percentage of total emissions of pollutants to soil occurring in areas at water risk	0.0	—
Percentage of total emissions of pollutants to soil occurring in areas of high-water stress	0.0	100.0
Percentage of total emissions of pollutants to water occurring in areas at water risk	2.8	100.0
Percentage of total emissions of pollutants to water occurring in areas of high-water stress	3.3	100.0
Percentage of total employees covered by collective bargaining agreements	46.3	100.0
Percentage of total Greenhouse gas emissions reduction (as of emissions of base year)	68.8	99.7
Percentage of variable remuneration dependent on sustainability-related targets and (or) impacts: ESRS E1 Climate change	37.5	99.9
Potential future liabilities, based on existing contractual agreements, associated with carbon credits planned to be cancelled in near future	0.9	71.2
Potential market size of low-carbon products and services or adaptation solutions to which undertaking has or may have access	2.6	83.3
Products and technical and biological materials used	15.8	51.5
Provisions for environmental protection and remediation costs: ESRS E2 Pollution	45.3	73.9
Radioactive waste	2.0	60.5
Rate of recordable work-related accidents: Employees	70.7	32.0
Rate of recordable work-related accidents: Non-employees	15.1	32.8
Remuneration ratio adjusted for purchasing power differences between countries	0.8	77.8
Renewable energy production	35.3	76.6
Reporting period end date	100.0	100.0
Reporting period start date	100.0	100.0
Revenue	99.1	84.1
Revenue from activities in high climate impact sectors	17.3	80.2
Revenue from activities other than in high climate impact sectors	31.3	83.3

Indicator	Disclosure detection rate (%)	Standardization rate (%)
Revenue from business activities at material physical risk	9.8	81.7
Revenue from business activities at material transition risk	11.1	83.6
Revenue from chemicals production	5.2	83.2
Revenue from coal	4.4	45.9
Revenue from controversial weapons	0.1	75.0
Revenue from cultivation and (or) production of tobacco	0.2	78.6
Revenue from customers operating in coal-related activities	1.0	53.3
Revenue from customers operating in gas-related activities	5.9	75.5
Revenue from customers operating in oil-related activities	5.0	82.2
Revenue from fossil fuel (coal, oil and gas) sector	10.7	73.5
Revenue from gas	11.5	50.0
Revenue from oil	7.2	67.0
Revenue from Taxonomy-aligned economic activities related to fossil gas	3.1	75.6
Sealed area	9.0	76.7
Secondary reused or recycled components, secondary intermediary products and secondary materials used to manufacture products and services	7.9	64.6
Significant CapEx for coal-related economic activities: ESRS E1 Climate change	5.1	74.5
Significant CapEx for gas-related economic activities: ESRS E1 Climate change	15.9	76.0
Significant CapEx for oil-related economic activities: ESRS E1 Climate change	10.6	79.7
Use of land area	21.7	76.8
Waste generated	59.0	90.6
Waste generated directed to disposal	55.2	84.7
Waste generated directed to disposal: Hazardous waste / Incineration	42.2	82.0
Waste generated directed to disposal: Hazardous waste / Landfill	56.2	79.7
Waste generated directed to disposal: Hazardous waste / Other disposal operations	50.5	84.2
Waste generated directed to disposal: Non-hazardous waste / Incineration	37.7	81.8
Waste generated directed to disposal: Non-hazardous waste / Landfill	61.7	79.6
Waste generated directed to disposal: Non-hazardous waste / Other disposal operations	57.8	84.0
Waste generated diverted from disposal	55.0	51.3
Waste generated diverted from disposal: Hazardous waste / Other recovery operations	54.2	65.6

Indicator	Disclosure detection rate (%)	Standardization rate (%)
Waste generated diverted from disposal: Hazardous waste / Preparation for reuse	48.4	67.6
Waste generated diverted from disposal: Hazardous waste / Recycling	62.1	53.8
Waste generated diverted from disposal: Non-hazardous waste / Other recovery operations	61.8	63.9
Waste generated diverted from disposal: Non-hazardous waste / Preparation for reuse	56.5	61.7
Waste generated diverted from disposal: Non-hazardous waste / Recycling	68.2	54.8
Water consumption	59.6	79.7
Water consumption in areas at water risk	18.2	69.8
Water consumption in areas of high-water stress	17.8	69.1
Water discharges	22.7	77.6
Water intensity (total water consumption per net revenue)	35.6	71.2
Water recycled and reused	21.7	55.9
Water stored	3.5	78.5
Water withdrawals	49.4	89.4

Notes: Disclosure detection rate is the share of indicator-company-year observations for which the pipeline detects a non-missing value-unit pair. Standardization rate is the share of indicator-company-year observations with detected disclosure for which the pipeline successfully standardizes the extracted value to a valid numeric value and unit consistent with the EFRAG specification.

Supplementary Information

S1 European Sustainability Reporting Standards (ESRS)

ESRS codify the reporting requirements for firms under the Corporate Sustainability Reporting Directive (CSRD, Directive EU 2022/2464). The successor of the Non-Financial Reporting Directive (NFRD, Directive EU 2013/34), the CSRD marks the largest-ever introduction of mandatory ESG reporting globally. It requires large, listed EU companies (i.e., listed companies that fulfill two of the three criteria: more than EUR 50 million in net turnover; more than EUR 25 million in assets;

or more than 250 employees) to comprehensively disclose their impacts on people and the planet as well as ESG-related risks and opportunities for their own business models. For these companies previously subject to the NFRD, the new rules are applicable from financial years starting in 2024 onward. Smaller and non-listed companies will face this mandate in later waves. A current EU reform proposal (COM(2025) 81) aims to reduce the reporting burden by exempting companies with up to 1,000 employees.

The disclosures are specified in two overarching (ESRS 1: General requirements and ESRS 2: General disclosures) and ten topical ESRS. The latter include environmental (ESRS E1: Climate change, ESRS E2: Pollution, ESRS E3 Water and marine resources, ESRS E4: Biodiversity, and ESRS E5: Circular economy), four social (ESRS S1: Own workforce, ESRS S2: Workers in the value chain, ESRS S3: Affected communities, and ESRS S4: Consumers), and one governance (ESRS G1: Business conduct) topics. In our analyses, we group indicators from ESRS 2 with the governance category to better align the ESRS topics with the public discourse.

Each topical ESRS requires companies to make disclosures about governance, strategy, management of impacts, risks, and opportunities, as well as metrics and targets related to the respective topic. Our list of n=501 indicators is derived from the accompanying XHTML taxonomy because the ESRS themselves do not provide a list of reportable indicators. Specifically, we used the ESRS XBRL Taxonomy Package published in August 2024 to derive our list of indicators. We validated our approach with a representative from the standard setter.

S2 ESG transparency, voluntary reporting, and the role of rating agencies

S2.1 ESG reporting before and after the EU Non-financial Reporting Directive

Our data show a relatively high level of ESG transparency already evident in 2014. This is partially explained by the widespread voluntary adoption of sustainability reporting frameworks well before the implementation of mandatory disclosure rules under the EU's Non-financial Reporting Directive (NFRD) [7], which only took effect for fiscal years starting in 2017.

Prior to the NFRD, many European companies voluntarily disclosed ESG information under globally recognized frameworks such as the Global Reporting Initiative (GRI) [8] and the United Nations Global Compact [9]. These frameworks promoted standardized reporting on ESG topics, especially on environmental metrics like energy usage, greenhouse gas emissions, and water consumption. While heterogeneous in structure and depth, this voluntary ESG reporting landscape laid the foundations for systematic data collection by third-party data providers and rating agencies [10].

The NFRD introduced a minimum set of mandatory disclosure requirements for large European firms, including key non-financial performance indicators relevant to the environment, social and employee matters, respect for human rights, and anti-corruption. However, the directive built on pre-existing voluntary practices rather than introducing a fundamentally new, rigorously audited and enforced reporting regime. Thus, the NFRD established a minimum standard for ESG transparency rather than a comprehensive upper benchmark. Despite extensive ESG disclosures by many firms prior to the legal mandate, existing studies indicate that the NFRD further enhanced transparency [11].

S2.2 Current state of ESRS digital reporting infrastructure

Although ESRS reporting has become mandatory for the first cohort of firms for fiscal year 2024 in European Union member states that have transposed the CSRD into national law [12], the digital reporting infrastructure for sustainability statements is still being phased in. In particular, the application of the European Single Electronic Format (ESEF) [13] to ESRS sustainability statements has been explicitly postponed, and ESRS sustainability information is therefore not yet required to be reported in a standardized, machine-readable XBRL/iXBRL format comparable to ESEF financial statements [14]. Consequently, ESRS disclosures remain predominantly narrative and PDF-based, often embedded in management reports.

Centralized access to sustainability reporting via the European Single Access Point (ESAP)

[15] is also not yet live and will be rolled out gradually, with full functionality expected only in later stages of implementation. Moreover, even once digital tagging and centralized access are fully implemented, these infrastructures are forward-looking and do not automatically make standardized, machine-readable data available retroactively for prior reporting years. This motivates the continued value of systematic extraction and validation approaches for constructing longitudinal baselines for pre-CSRD and early-transition disclosures.

S2.3 ESG data collection by rating agencies

ESG rating agencies rely heavily on publicly available corporate disclosures—such as annual reports, sustainability reports, and websites—as a primary input to their evaluations. Agencies such as MSCI, Refinitiv, Sustainalytics, Moody's ESG (Vigeo-Eiris), S&P Global (RobecoSAM), and KLD base their ratings on hundreds of individual ESG indicators [16]. While public disclosures form a critical input, rating agencies also supplement these with other sources, including: (1) company questionnaires, the responses to which are typically not made public, and (2) third-party and media reports. Notably, even when using the same publicly disclosed data, raters may apply different scoring algorithms, which contributes to variation in ESG ratings across providers. Rating disagreement can be decomposed into three components: scope (what is measured), measurement (how it is measured), and weights (how indicators are aggregated). As shown in existing work, measurement divergence and scope divergence strongly account for the variation, but weight divergence less so [16].

S2.4 Implications for standardized disclosure and baseline transparency

One possible explanation for the high baseline ESG transparency observed in the early years is that firms engaged in box-ticking behavior—that is, reporting on standardized, easily measurable indicators in response to the expectations of data providers and investors. Such disclosures may have been sufficient to satisfy prevailing ESG rating methodologies. Commonly reported indica-

tors across providers include water usage, labor practices, product safety, and energy consumption. However, deeper variation across firms and raters appears in more complex or controversial ESG domains, such as tax policy, biodiversity, or lobbying activities. These domains are often more difficult to measure and less frequently disclosed voluntarily, limiting their use as differentiators unless mandated.

Overall, the relatively high levels of ESG transparency observed in 2014 reflect both voluntary disclosure traditions and selective reporting on standardized, easily measurable indicators. ESG rating agencies rely heavily on such public disclosures, but also incorporate proprietary inputs like questionnaires and media-based analytics. Because measurement methods vary substantially across raters, transparency may be overestimated if assessed through coarse, undifferentiated metrics. This highlights the need for caution when interpreting ESG ratings or disclosure volumes as proxies for meaningful transparency or performance.

S3 Prompt design

The prompt is based on best practices in LLM prompt design [17, 18]. It consists of a system prompt and a user prompt. The system prompt details the role that the model should assume and explains the task it should perform. Furthermore, clear instructions on the output format are given, followed by specific examples. The model is instructed to refrain from generating speculative responses in case the requested ESG indicator is absent from the corporate report. The user prompt specifies the company name and reporting year of the corresponding corporate report. In addition, it contains the top-k most relevant report chunks retrieved by the hybrid search method and the description of the requested ESG indicator. Thus, the prompt design limits model hallucination and guarantees scalability for the large-scale RAG implementation in this work. Both our system prompt and user prompt are presented in Fig. S31.

System prompt

You are a senior researcher who specializes in extracting numeric data points from corporate reports to meet the European Sustainability Reporting Standards (ESRS). Your task is to identify the correct value and unit of the requested data point from the provided corporate report chunks.

Give a single, definite answer in JSON format with the keys "value" and "unit". "value" is a single floating-point value without any commas or spaces between the digits. If the requested data point is a date, "value" is a string in the form "YYYY-MM-DD". "unit" is always a string.

For example: {{"value": 281002.0, "unit": "tCO2e"}} or {{"value": 15.4, "unit": "%"}} or {{"value": 1118.0, "unit": null}}.

Important note: If you cannot find information on the requested data point in the provided corporate report, respond with: {{"value": null, "unit": null}}.

Use only the provided corporate report in its entirety to find information on the requested data point.

User prompt

Corporate report ({company}, year: {year}): {context}

Requested data point: {{request}}

Answer:

Fig. S31. Prompt design. The system prompt (top) specifies the role, extraction task, and guidelines to be observed by the LLM. The user prompt (bottom) includes the company name and reporting year, the top-k retrieved report chunks (context), and the ESG indicator to be extracted (requested value).

S4 Methodological limitations

ML-based document processing is evolving rapidly, yet robust extraction from complex, heterogeneous corporate reports remains challenging. First, our ML framework relies on automatic parsing of PDF reports and is therefore sensitive to document layout. Complex formatting (e.g., multi-column text, embedded or rotated tables, footnotes) can lead to imperfect text reconstruction and, in turn, missed or misread values. Second, the performance depends on retrieval: relevant information may not be returned if it is dispersed across distant sections or expressed indirectly. To mitigate the risk of context incompleteness, we use overlapping chunks and expand the retrieved chunks with their preceding and subsequent chunks, respectively. Third, LLM-based extraction remains sensitive to ambiguous indicators and heterogeneity in definitions. Companies may describe conceptually similar indicators using different reporting scopes and conventions (e.g., organiza-

tional boundary, consolidation scope, intensity versus absolute metrics), and disclosures may only partially match the target indicator definition—creating ambiguity for the LLM and increasing the risk of extracting a plausible but non-equivalent value. We mitigate this by passing ESRS-aligned indicator descriptions to the model via a tailored prompt. Fourth, corporate disclosures frequently contain multiple time references (current year, prior year comparatives; multi-year KPI tables). Even if the correct value is present, attributing it to the intended reporting period can be ambiguous. We address this by structuring extraction at the document–indicator level and explicitly highlighting the reporting period in a tailored prompt. Finally, as with generative models more generally, the LLM can produce plausible-looking outputs even when evidence is weak or missing (i.e., hallucinate). To limit hallucination, we use conservative prompting that explicitly instructs the model not to return a value when the requested indicator is absent from the corporate report. Because residual extraction errors can persist despite these safeguards, rigorous validation is essential. Accordingly, we validated LLM-extracted values against both a proprietary dataset and an expert-annotated subset.

Supplementary References

[1] Sustainability Accounting Standards Board. Find your industry (2025). URL https://sasb.ifrs.org/find-your-industry/.

[2] MSCI Inc. ESG ratings (2025). URL https://www.msci.com/data-and-analytics/sustainability-solutions/esg-ratings.

[3] London Stock Exchange Group. ESG data (2025). URL https://www.lseg.com/en/data-analytics/financial-data/company-data/esg-data.

[4] London Stock Exchange Group. Environmental, social and governance scores from LSEG (2024). URL https://www.lseg.com/content/dam/data-analytics/en_us/documents/methodology/lseg-esg-scores-methodology.pdf.

[5] London Stock Exchange Group. Worldscope Fundamentals (2025). URL https://www.lseg.com/en/data-analytics/financial-data/company-data/fundamentals-data/worldscope-fundamentals.

[6] Eurostat. HICP (2025). URL https://ec.europa.eu/eurostat/databrowser/view/prc_hicp_aind__custom_16284184/default/table?lang=en.

[7] European Parliament and the Council of the European Union. Directive 2014/95/EU of the European Parliament and of the Council of 22 October 2014 amending Directive 2013/34/EU as regards disclosure of non-financial and diversity information by certain large undertakings and groups (text with EEA relevance). *Official Journal of the European Union* **L330**, 1–9 (2014).

[8] Global Reporting Initiative. GRI Standards (2025). URL https://www.globalreporting.org/standards/.

[9] United Nations Global Compact. UN Global Compact (2025). URL https://unglobalcompact.org/.

[10] Sellhorn, T. & Wagner, V. The forces that shape mandatory ESG reporting. In *Research Handbook on Environmental, Social and Corporate Governance*, 269–292 (Edward Elgar Publishing, Cheltenham, UK, 2024).

[11] Fiechter, P., Hitz, J.-M. & Lehmann, N. Real effects of a widespread CSR reporting mandate: evidence from the European Union's CSR Directive. *Journal of Accounting Research* **60**, 1499–1549 (2022).

[12] European Parliament and the Council of the European Union. Directive (EU) 2022/2464 of the European Parliament and of the Council of 14 December 2022 amending Regulation (EU) No 537/2014, Directive 2004/109/EC, Directive 2006/43/EC and Directive 2013/34/EU, as regards corporate sustainability reporting (text with EEA relevance). *Official Journal of the European Union* **L322**, 15–80 (2022).

[13] European Commission. Commission Delegated Regulation (EU) 2018/815 of 17 December 2018 supplementing Directive 2004/109/EC of the European Parliament and of the Council with regard to regulatory technical standards on the specification of a single electronic reporting format (text with EEA relevance). *Official Journal of the European Union* **L143**, 1–792 (2019).

[14] European Financial Reporting Advisory Group (EFRAG). Digital reporting with XBRL (2025). URL https://www.efrag.org/en/sustainability-reporting/esrs-workstreams/digital-reporting-with-xbrl.

[15] European Parliament and the Council of the European Union. Regulation (EU) 2023/2859 of the European Parliament and of the Council of 13 December 2023 establishing a European single access point providing centralised access to publicly available information of relevance

to financial services, capital markets and sustainability (text with EEA relevance). *Official Journal of the European Union* (2023).

[16] Berg, F., Kölbel, J. F. & Rigobon, R. Aggregate confusion: the divergence of ESG ratings. *Review of Finance* **26**, 1315–1344 (2022).

[17] Lin, Z. How to write effective prompts for large language models. *Nature Human Behaviour* **8**, 611–615 (2024).

[18] Giray, L. Prompt engineering with ChatGPT: a guide for academic writers. *Annals of Biomedical Engineering* **51**, 2629–2633 (2023).
