# Supplementary material for: Assessing corporate sustainability with large language models: evidence from Europe
Source: Nat Commun. 2026 Jul 7;17:5940. doi: 10.1038/s41467-026-75160-z (PMC13341771; doi:10.1038/s41467-026-75160-z)
Supplement: Supplementary file 2 — Reporting Summary [file 41467_2026_75160_MOESM2_ESM.pdf]

nature portfolio

Corresponding author(s): Stefan Feuerriegel

Last updated by author(s): 13.05.2026

Reporting Summary

Nature Portfolio wishes to improve the reproducibility of the work that we publish. This form provides structure for consistency and transparency in reporting. For further information on Nature Portfolio policies, see our Editorial Policies and the Editorial Policy Checklist.

Statistics

For all statistical analyses, confirm that the following items are present in the figure legend, table legend, main text, or Methods section.

n/a	Confirmed	
☐	☒	The exact sample size (*n*) for each experimental group/condition, given as a discrete number and unit of measurement
☐	☒	A statement on whether measurements were taken from distinct samples or whether the same sample was measured repeatedly
☐	☒	The statistical test(s) used AND whether they are one- or two-sided<br>*Only common tests should be described solely by name; describe more complex techniques in the Methods section.*
☐	☒	A description of all covariates tested
☐	☒	A description of any assumptions or corrections, such as tests of normality and adjustment for multiple comparisons
☐	☒	A full description of the statistical parameters including central tendency (e.g. means) or other basic estimates (e.g. regression coefficient) AND variation (e.g. standard deviation) or associated estimates of uncertainty (e.g. confidence intervals)
☐	☒	For null hypothesis testing, the test statistic (e.g. *F*, *t*, *r*) with confidence intervals, effect sizes, degrees of freedom and *P* value noted<br>*Give P values as exact values whenever suitable.*
☒	☐	For Bayesian analysis, information on the choice of priors and Markov chain Monte Carlo settings
☐	☒	For hierarchical and complex designs, identification of the appropriate level for tests and full reporting of outcomes
☐	☒	Estimates of effect sizes (e.g. Cohen's *d*, Pearson's *r*), indicating how they were calculated

Our web collection on statistics for biologists contains articles on many of the points above.

Software and code

Policy information about availability of computer code

Data collection	Data collection was conducted using custom Python v3.12.10 code, which is publicly available in the GitHub repository listed in the Code Availability Statement. The machine learning pipeline used PyMuPDF v1.24.9 for PDF parsing, LangChain packages for document processing and model access (langchain-community v0.2.12, langchain-text-splitters v0.2.2, langchain-huggingface v0.0.3 and langchain-together v0.1.5), sentence-transformers v3.1.0 with the pre-trained all-MiniLM-L12-v2 embedding model for vector representations, FAISS v1.8.0.post1 for vector retrieval, rank-bm25 v0.2.2 for keyword-based retrieval, Llama-Rank-V1 for re-ranking, and Llama-3.1-70B-Instruct accessed via the Together AI API using together v1.3.3 for ESG indicator extraction. Postprocessing used regular expressions and Pint v0.24.4 for unit standardization.
Data analysis	Data analysis was conducted using custom Python v3.12.10 and R v4.4.0 code, which is publicly available in the GitHub repository listed in the Code Availability Statement.<br>Statistical analyses, data processing and figure generation were performed using custom analysis scripts in Python v3.12.10 and the following Python packages: pandas v2.2.2, numpy v1.26.4, matplotlib v3.9.2, plotly v5.24.1, fastparquet v2024.11.0, Pillow v10.4.0, PyYAML v6.0.2, requests v2.32.3, clean-text v0.6.0, langdetect v1.0.9, textstat v0.7.4 and unidecode v1.3.8. The Python environment also used torch v2.5.0, ipykernel v6.29.5, notebook v7.4.4 and setuptools v72.1.0. Exact software versions and installation requirements are provided in the requirements.txt file in the public GitHub repository.<br>R-based analyses, tables and figures used R v4.4.0 and the following R packages: tidyr v1.3.1, dplyr v1.1.4, readr v2.1.5, ggplot2 v3.5.1, tinytable v0.6.1, kableExtra v1.4.0, modelsummary v2.2.0, fixest v0.12.1, here v1.0.1, patchwork v1.2.0, scales v1.3.0, data.tree v1.1.0, DiagrammeR v1.0.11, vtree v5.6.5, xtable v1.8-4, ggnewscale v0.5.1, forcats v1.0.0, purrr v1.0.2, gridExtra v2.3, stringr v1.5.1, rlang v1.1.3, tinytex v0.50, psych v2.5.6, latex2exp v0.9.6, yaml v2.3.8 and jsonlite v1.8.8.

For manuscripts utilizing custom algorithms or software that are central to the research but not yet described in published literature, software must be made available to editors and reviewers. We strongly encourage code deposition in a community repository (e.g. GitHub). See the Nature Portfolio guidelines for submitting code & software for further information.

Data

Policy information about availability of data

All manuscripts must include a data availability statement. This statement should provide the following information, where applicable:

- Accession codes, unique identifiers, or web links for publicly available datasets
- A description of any restrictions on data availability
- For clinical datasets or third party data, please ensure that the statement adheres to our policy

The complete dataset of extracted ESG indicators generated in this study has been deposited in the Open Science Framework (OSF) repository under accession code q2jpv (https://osf.io/q2jpv/). The corporate annual and sustainability reports used in this study have been deposited in Harvard Dataverse under accession code DVN/84HKPS (https://doi.org/10.7910/DVN/84HKPS). The remaining third-party datasets used for validation and additional analyses are available under restricted access because they are proprietary and require a subscription or license; access can be obtained from the respective data providers. Specifically, the LSEG Refinitiv ESG data used for validation are available from LSEG (https://www.lseg.com/en/data-analytics/financial-data/company-data/esg-data); company fundamentals data are available from LSEG Worldscope Fundamentals (https://www.lseg.com/en/data-analytics/financial-data/company-data/fundamentals-data/worldscope-fundamentals); ESG controversies scores are available from LSEG ESG Scores (https://www.lseg.com/en/data-analytics/sustainable-finance/esg-scores); and MSCI ESG ratings are available from MSCI (https://www.msci.com/data-and-analytics/sustainability-solutions/esg-ratings).

Research involving human participants, their data, or biological material

Policy information about studies with human participants or human data. See also policy information about sex, gender (identity/presentation), and sexual orientation and race, ethnicity and racism.

Reporting on sex and gender	This study did not involve human participants or human data.
Reporting on race, ethnicity, or other socially relevant groupings	This study did not involve human participants or human data.
Population characteristics	This study did not involve human participants or human data.
Recruitment	This study did not involve human participants or human data.
Ethics oversight	This study did not involve human participants or human data.

Note that full information on the approval of the study protocol must also be provided in the manuscript.

Field-specific reporting

Please select the one below that is the best fit for your research. If you are not sure, read the appropriate sections before making your selection.

☐ Life sciences ☐ Behavioural & social sciences ☒ Ecological, evolutionary & environmental sciences

For a reference copy of the document with all sections, see nature.com/documents/nr-reporting-summary-flat.pdf

Ecological, evolutionary & environmental sciences study design

All studies must disclose on these points even when the disclosure is negative.

Study description	This study develops and validates a machine learning pipeline to extract quantitative environmental, social and governance (ESG) indicators from corporate annual and sustainability reports. The analysis covers the 600 largest listed companies in Europe over 2014–2023. The pipeline tracks 501 ESRS-aligned ESG indicators and is used to study ESG-related transparency and performance over time, across industries, and across company characteristics. The study is observational and descriptive rather than experimental.
Research sample	The research sample consists of all companies listed in the STOXX Europe 600 stock index in 2023. This yields 600 large listed European firms from 16 countries and 11 industry sectors, covering nearly 90% of the investable equity market in Europe. For these firms, the study collects annual and sustainability reports published as PDF files between 2014 and 2023 (9,173 documents in total).
Sampling strategy	The study uses a population-based sample of all STOXX Europe 600 constituents as of 2023 rather than drawing a random sample. This sample was chosen because the index covers the largest listed European firms and represents nearly 90% of the investable European equity market by market capitalization.
Data collection	Corporate annual and sustainability reports were collected from publicly available sources, including https://annualreports.com, https://sustainabilityreports.com, and companies' own websites. Company fundamentals were obtained from Worldscope, ESG controversies scores from LSEG Refinitiv, and ESG ratings from MSCI. For validation, extracted ESG values were compared with a proprietary LSEG Refinitiv ESG dataset and with an expert human-annotated subset. Manual annotations were produced independently by two PhD-level researchers familiar with ESRS reporting, and the full subset was also independently annotated by a third annotator to assess inter-rater reliability.
Timing and spatial scale	Data collection covers reporting years 2014–2023 at annual frequency. The spatial scale is Europe and includes companies from 16

countries represented in the STOXX Europe 600.

Data exclusions	Analyses were limited to company-years for which at least one annual or sustainability report was publicly available. For pipeline processing, company-year documents were retained if text extraction produced at least 5,000 characters and the indexed document contained at least 50 chunks, to ensure that documents contained sufficient textual content for meaningful retrieval and extraction. For performance analyses, indicator-company-year observations were included only when a numerical value could be extracted and standardized to the relevant unit. No additional exclusions were applied.
Reproducibility	The study supports reproducibility by providing the full machine learning pipeline, data processing scripts and analysis code in the GitHub repository listed in the Code Availability Statement. The extracted ESG indicator dataset is available via OSF, and the corporate report corpus is available via Harvard Dataverse. The inference model was run with temperature set to 0 to promote deterministic output. Software versions are listed in the software section and repository files.
Randomization	Randomization was not relevant because the study is observational and descriptive. Companies were not assigned to experimental groups or interventions. Group comparisons, such as top and bottom deciles of ESG ratings or market capitalization, were based on observed company characteristics.
Blinding	Blinding was not relevant for the machine learning extraction and quantitative analyses because there was no experimental group allocation.

Did the study involve field work? ☐ Yes ☒ No

Reporting for specific materials, systems and methods

We require information from authors about some types of materials, experimental systems and methods used in many studies. Here, indicate whether each material, system or method listed is relevant to your study. If you are not sure if a list item applies to your research, read the appropriate section before selecting a response.

Materials & experimental systems

n/a	Involved in the study	
☒	☐	Antibodies
☒	☐	Eukaryotic cell lines
☒	☐	Palaeontology and archaeology
☒	☐	Animals and other organisms
☒	☐	Clinical data
☒	☐	Dual use research of concern
☒	☐	Plants

Methods

n/a	Involved in the study	
☒	☐	ChIP-seq
☒	☐	Flow cytometry
☒	☐	MRI-based neuroimaging

Plants

Seed stocks	This study did not involve plants, seed stocks, or other plant material.
Novel plant genotypes	This study did not involve plants, seed stocks, or other plant material.
Authentication	This study did not involve plants, seed stocks, or other plant material.
