# Supplementary material for: Assessing corporate sustainability with large language models: evidence from Europe
Source: Nat Commun. 2026 Jul 7;17:5940. doi: 10.1038/s41467-026-75160-z (PMC13341771; doi:10.1038/s41467-026-75160-z)
Supplement: Supplementary file 3 — Transparent Peer Review file [file 41467_2026_75160_MOESM3_ESM.pdf]

nature portfolio

Peer Review File

Assessing corporate sustainability with large language models: Evidence from Europe

Corresponding Author: Professor Stefan Feuerriegel

This file contains all reviewer reports in order by version, followed by all author rebuttals in order by version.

Version 0:

Reviewer comments:

Reviewer #1

(Remarks to the Author)
The manuscript creates a machine-extracted data set of 9K annual and sustainability reports from 2014 to 2023 of STOXX 600 companies, and provides various descriptive statistics about the collected data.

There are clear strengths:

- The general topic of highlighting progress in sustainability reporting and results over the years can present a positive incentive for key private sector contributions, complementing regulation and compliance studies focusing primarily on the negative cases.

- The specific descriptive statistics presented on the data set highlight slow but steady progress in both sustainability reporting practices and in sustainability KPIs across all countries and sectors. Again, this presents a welcome positive, albeit half-full glass, narrative that supports the extraordinary efforts of EU policy makers, emerging sustainability standards on reporting, and on key sustainability indicators that help the private sector in the transformations needed.

- There is significant methodological novelty in the paper, which goes far beyond traditional scientific hypothesis testing against independent human-verified data and evidence collection, with explicit hypotheses and clearly defined populations, and rigorous statistical analysis of what conclusions are warranted by the data.

- In contrast, the paper makes the important, but implicit, assumption that big data at scale, which is necessarily lacking in data quality, can serve as a basis for meaningful analysis by providing a far larger volume of data quantity. This is significantly pushing the frontier in methodology beyond what is traditionally accepted as evidence warranting scientific conclusions, and bridging to standard engineering methodologies in the tech sector and elsewhere that go beyond methodological guarantees upfront, and essentially rely on the “proof is in the pudding” approaches: just do it, and it works if it works.

- A particular strength is the limited validation in the second half of the paper, demonstrating low but significant correlation between machine-extraction and human-annotation, and reasonable overall correlation with a proprietary (presumably supervised) data set. This indicates the machine extraction is demonstrably better than random, and although far from identical to human annotations or error-free data, it can be a basis for meaningful analysis and potentially yield interesting hypotheses to be validated in follow-up research.

- A wealth of data is made available, allowing other researchers to reproduce the research and to examine the quality of the data analysis more closely.

There are some limitations:

- My main quarrel is with the organization of the manuscript. The paper first documents an interesting descriptive analysis of a very particular data set and then, in the second half, details the precise construction of this data set. The second half details all the ad hoc pragmatic engineering choices necessitated by the construction of an extensive data set without human supervision. Knowing the engineering choices and their limitations feels necessary for interpreting the analysis of the

resulting data set, which validates these pragmatic engineering choices as not unreasonable.

- The positive enthusiasm about emerging methodological opportunities that readers feel throughout the paper is a key strength that should not be lost by endless stressing of non-trivial limitations, provisos, and qualifications. However, showing awareness of the significant limitations at several key points would significantly strengthen the paper's contributions in clarity and impact. Let me limit my suggestion to the framing and key methodological aspects, more clearly carving out the contributions of the current data and analysis.

- First, in terms of framing and novelty, a slight straw man feeling of overstated novelty in the sense that the 2024 ESRS standard is now mandatory and includes the Sustainability Reporting in a Digital Format (ESEF) with standardized and machine-readable data as reported directly by each company. This makes the papers' approach focus only on historic, legacy data (rather than current data), and most of the issues as reported in the paper should no longer apply. In addition. While the current paper's focus differs, there is endless related research on technically highly advanced LegalTech, FinTech, and AI approaches in the literature. E.g., publicly traded companies deposit any contract in platforms like EDGAR (US), Sedar (Canada), or similar platforms, which are widely used in AI/ML/NLP research, and many data sets and advanced document processing pipelines are available. Here, critically evaluating the quality of information extraction is central. And the state of the art in AI technology is progressing fast, but it is still far from perfect.

- Second, the ultimate dataset is a combination of selection bias on the population of included organizations (so the population is one set of STOXX 600 companies rather than whole sectors or industries, or continents), the success of the crawling strategy (e.g., not even all annual reports were included according to Fig. S3), the success of the OCR/PDF text extraction (this is never 100% or may be scrambled), the choice of the chunker (with very brief passages, which may work well for some but not all indicators), the success of the information extraction strategy (with just one prompt for each indicator), the success of the data normalization strategy (which may appear in many units and variants), etc, etc. Anything that looks like a simple problem turns out to be an imperfect extraction in reality, once you start measuring data (extraction) quality against ground truth. E.g., think of sentences mentioning both last year's and this year's numbers, or report in relative gains, or think of complex table extraction or getting numbers from images. As a result, the final data is the combination of the real-world data and a range of extraction errors (which we normally very carefully quantify against human reference data). With any analysis of this data, we must be aware that we are underreporting, which can result in selection bias. Moreover, the imperfection in the processing may not be equally distributed over variables, which can explain some of the observed variance.

- Third, while the exploratory data analysis is interesting, a more critical discussion and awareness of limitations could be possible. For example, there are many potential confounding effects both in the data and outside the explicit variables. A more critical discussion of alternative explanations and what exact hypothesis the data suggests would strengthen the analysis. E.g., if a recency effect is observed, due to more reporting or better extraction quality, or a size effect, subsequent analysis could be sampled to compensate for this effect, allowing for the analysis of the independent contribution of other factors or indicators.

- The analysis makes a large number of engineering choices that are clearly documented and are each an entirely reasonable and pragmatic choice in themselves. The strength of this is that it enables novel analysis at a far larger scale, and far less human time and effort in annotation. This is a key strength that can accelerate research significantly. With a slightly more critical reporting showing more awareness of both the strengths and limitations of this methodological choice, the manuscript can make an essential contribution. A request for more critical reflection on the methodology may be unfair, as no paper using traditional methods will detail textbook-level explanations of basic methodology. When moving to a new "AI paradigm," the onus of accounting for the new methodology lies with those venturing into this new area. Perhaps undeservedly, this comes with a responsibility to very critically account for both strengths and limitations of the new methodology — just to fully inform the reader, and allow them to interpret the results and evidence on which these are based to their true value. Similar to the emerging standards on reporting sustainability efforts studied in the paper, we can only expect the highest levels of transparency and detail on the consequences of the combined limitations.

(Remarks on code availability)
Just a bunch of csv files.

Didn't check the raw download, nor run these through a standard pipeline from the literature.

The authors' processing reveals great effort and care (but also limited experience in large-scale document processing and information extraction in a technical field).

Reviewer #2

(Remarks to the Author)
I was very appreciative of having the opportunity to read and provide a view on this paper. I appreciated the extensive amount of work that has gone into the paper, the clarity of the description of the methods and the significance of the findings. I was also very keen on the open access dataset that will accompany the paper: this will be a hugely valuable resource for researchers within and beyond the corporate accountability field.

There are two minor points that I would like to see addressed in the paper. First, some more detail on the LLM used to create the core data would be helpful. For example, if this is a dedicated model developed for this work or some external model. I

think readers will be really interested in knowing about this – apologies if it is tucked away in SMs and I missed it.

The second point is a handful of places in the text where ‘improvements or ‘negative’ trends are noted in disclosure. I would prefer that these are increases or decreases rather than descriptions associated with value judgements. Most readers would agree with your judgement, but it should be left to them to decide how they understand an increase/decrease.

(Remarks on code availability)

Version 1:

Reviewer comments:

Reviewer #2

(Remarks to the Author)
The additional work undertaken has strengthened the manuscript. The findings are nuanced and insightful, providing a rich description of an extended dataset of reporting practice. In addition, the open source aspect of this work means that this material will have a flow on positive effect for other studies in the future. Finally, the work has advanced thinking about methodological aspects on how and which reporting framework items should be considered.

(Remarks on code availability)
I lack the technical expertise to review the code.

Open Access This Peer Review File is licensed under a Creative Commons Attribution 4.0 International License, which permits use, sharing, adaptation, distribution and reproduction in any medium or format, as long as you give appropriate credit to the original author(s) and the source, provide a link to the Creative Commons license, and indicate if changes were made.
In cases where reviewers are anonymous, credit should be given to 'Anonymous Referee' and the source.
The images or other third party material in this Peer Review File are included in the article’s Creative Commons license, unless indicated otherwise in a credit line to the material. If material is not included in the article’s Creative Commons license and your intended use is not permitted by statutory regulation or exceeds the permitted use, you will need to obtain permission directly from the copyright holder.
To view a copy of this license, visit https://creativecommons.org/licenses/by/4.0/

Revised submission to *Nature Communications*

Manuscript: "Assessing corporate sustainability with large language models: Evidence from Europe" (NCOMMS-25-72275-T)

Dear Reviewers,

We are deeply grateful for your helpful and detailed reviews of the previous version of our paper. We especially appreciate the constructive feedback highlighting specific areas in need of improvement and have taken it very seriously. To address the concerns raised by the reviewers, we have made substantial revisions to bring the paper to a substantially higher standard of quality by closely following your comments.

Based on your feedback, we have improved our manuscript in the following ways:

- **Added cross-references to literature using NLP in finance:** We added additional cross-references to prior work using natural language processing (NLP) in finance to sharpen our positioning relative to the literature. While there is a substantial body of research in finance, accounting, and legal studies applying NLP to analyze regulatory filings (see overviews in Loughran and McDonald, 2016, and Bochkay et al., 2023), the focus of these works differs from ours. Existing works primarily apply NLP for sentiment or readability analysis, which falls under the task of *text classification*. In contrast, monitoring ESG performance, as in our work, is a different task and requires the extraction of structured quantitative information, which is a *retrieval and extraction task*. We clarify this methodological distinction in the Introduction to better delineate our contribution.
- **Clarified the novelty and value of our analyses in the light of CSRD/ESRS transition and the limits of digital reporting requirements.** We revised the manuscript to better contextualize the premise that firm-level, standardized, machine-readable ESRS data are already broadly available. Specifically, we now clarify that
    - ESRS reporting since fiscal year 2024 applies only to the first cohort of large, publicly traded firms in EU member states that have transposed the CSRD into national law;
    - the mandatory digital tagging of ESRS sustainability statements in a standardized electronic format has been explicitly postponed; and
    - the European Single Access Point (ESAP)—the planned EU-wide platform for centralized access to sustainability disclosures—is not yet live and will only be rolled out gradually in the coming years.

  Accordingly, at present sustainability disclosures remain predominantly PDF-based, often embedded in management reports, and systematic indicator extraction and validation remain necessary. We therefore position our dataset as a longitudinal baseline for the pre-CSRD and early-transition period that enables research and benchmarking of how reporting practices evolve as ESRS adoption and digital infrastructure are phased in.

- **Additional method overview.** We added a brief methodological overview at the beginning of Section 2, so that key elements of the extraction pipeline and its assumptions are introduced before the descriptive findings. This structure follows common practice in Nature-style journals, where, different from many other journals, the actual Methods section is normally placed at the end of the manuscript. Nevertheless, we felt that providing an early overview—together with a concise description of our task, pipeline design, and validation approach—helps readers understand how the data are generated without requiring them to consult the full Methods section first. In particular, we now reference the validation early on to clarify the reliability of the extracted indicators before presenting substantive patterns.

- **Additional pipeline diagnostics.** We now report additional end-to-end pipeline diagnostics, including stage-wise coverage of the extraction process (new Supplementary Fig. S6). Specifically, we document how observations (i.e., individual reports) progress through each step of the pipeline—from report retrieval and text extraction to chunking, disclosure detection, and standardization—and where information may drop out. In addition, we report per-indicator disclosure detection and standardization rates (new Supplementary Table S6) to document heterogeneity in downstream standardization success across indicators. Together, these diagnostics provide greater transparency about the mechanics and reliability of the pipeline. Overall, we note that potential errors during extraction are rare, which further corroborates the robustness of our pipeline. We also added a short summary of this analysis to Section 2, so that readers can understand early what the strengths and limitations of our LLM approach are.
- **Revised interpretation without normative claims.** We also revised the framing throughout the Results and Discussion sections to distinguish empirical patterns from interpretation and to describe temporal patterns in neutral terms (e.g., "increase" instead of "improve"; in response to Reviewer R2). We emphasize that our analyses are descriptive and do not support causal claims. In the revised Discussion, we therefore state more explicitly which hypotheses the documented transparency and performance patterns suggest and discuss potential alternative explanations and confounding influences. At the same time, we clarify the main purpose of our analysis: that is, to demonstrate the scientific value of the dataset for downstream research, including hypothesis development and subsequent causal analysis. We also state more explicitly which types of questions the descriptive evidence may motivate.
  In addition, we moved a key regression analysis from the Supplementary Materials into the main manuscript to more clearly demonstrate the scientific value of the dataset for empirical research (see Table 1). Specifically, the regression analysis quantifies the association between transparency measures and firm characteristics and assesses whether the observed descriptive patterns hold in a multivariate panel framework. By presenting this analysis in the main text, we aim to illustrate how the dataset can support systematic, theory-driven downstream analyses rather than serving solely as descriptive evidence.
- **Extended robustness checks.** We have added a set of robustness analyses to assess whether key patterns are sensitive to (i) recency-related mechanical changes over time (expanding disclosure incidence or improving document-to-text quality), or (ii) observable firm-level heterogeneity that could confound descriptive trends. First, we assess potential recency effects by re-estimating year-to-year trends within the subset of constant reporters for each indicator and on a restricted sample of company–year observations with high document text quality (new Supplementary Fig. S18–S20 and S7–S12); here, the temporal patterns closely mirror the full-sample results, which corroborates our findings. Second, we re-estimate firm-year panel regressions of transparency under increasingly restrictive controls and (year, sector, year-by-sector, and firm) fixed effects and assess differential trajectories by interacting ESG-rating strata with a linear time trend (new Table 1; new Supplementary Table S2). Overall, these checks indicate robustness to plausible variation in disclosure incidence, document text quality, and observable firm heterogeneity.
- **Expanded LLM documentation**: Following Reviewer R2, we have also expanded the Methods section to ensure that our LLM components are described comprehensively and transparently. For this, we reviewed our Methods section against a recent, consensus-based LLM reporting checklist (GUIDE-LLM; http://www.llm-checklist.com/llm-checklist.pdf) to ensure comprehensive and transparent documentation of the model component. Where appropriate, we clarified and made more explicit existing descriptions (e.g., model selection, prompting setup, parameter choices, and postprocessing) to improve completeness and reproducibility. We now provide the completed reporting checklist in our code base to document alignment with established reporting standards.

Across all improvements, our key findings are robust: ESG transparency has increased but remains uneven across topics and industries. At the same time, ESG performance exhibits mixed trends, with substantial decreases in some areas.

We provide point-by-point responses below. To facilitate the review, we highlighted key changes in our manuscript in **blue text color**.

RESPONSES TO REVIEWER #1

Comment R1.0: *"The manuscript creates a machine-extracted data set of 9K annual and sustainability reports from 2014 to 2023 of STOXX 600 companies, and provides various descriptive statistics about the collected data.*

There are clear strengths:

- The general topic of highlighting progress in sustainability reporting and results over the years can present a positive incentive for key private sector contributions, complementing regulation and compliance studies focusing primarily on the negative cases.

- The specific descriptive statistics presented on the data set highlight slow but steady progress in both sustainability reporting practices and in sustainability KPIs across all countries and sectors. Again, this presents a welcome positive, albeit half-full glass, narrative that supports the extraordinary efforts of EU policy makers, emerging sustainability standards on reporting, and on key sustainability indicators that help the private sector in the transformations needed.

- There is significant methodological novelty in the paper, which goes far beyond traditional scientific hypothesis testing against independent human-verified data and evidence collection, with explicit hypotheses and clearly defined populations, and rigorous statistical analysis of what conclusions are warranted by the data.

- In contrast, the paper makes the important, but implicit, assumption that big data at scale, which is necessarily lacking in data quality, can serve as a basis for meaningful analysis by providing a far larger volume of data quantity. This is significantly pushing the frontier in methodology beyond what is traditionally accepted as evidence warranting scientific conclusions, and bridging to standard engineering methodologies in the tech sector and elsewhere that go beyond methodological guarantees upfront, and essentially rely on the "proof is in the pudding" approaches: just do it, and it works if it works.

- A particular strength is the limited validation in the second half of the paper, demonstrating low but significant correlation between machine-extraction and human-annotation, and reasonable overall correlation with a proprietary (presumably supervised) data set. This indicates the machine extraction is demonstrably better than random, and although far from identical to human annotations or error-free data, it can be a basis for meaningful analysis and potentially yield interesting hypotheses to be validated in follow-up research.

- A wealth of data is made available, allowing other researchers to reproduce the research and to examine the quality of the data analysis more closely.
"

Response R1.0: Thank you for the positive assessment of our work. We appreciate your feedback on the contributions and methodological novelty. We took your comment on the implicit "quantity-over-quality" assumption to heart and further strengthened the evidentiary basis for our findings by adding new end-to-end pipeline coverage diagnostics (Supplementary Fig. S6) and robustness checks (indicator-level extractability diagnostics, balanced panels, document-quality restrictions, and panel regressions; Table 1, Supplementary Tables S2 and S6, Supplementary Fig. S7–S12 and S18–S20).

Comment R1.1: *"There are some limitations:*

- My main quarrel is with the organization of the manuscript. The paper first documents an interesting descriptive analysis of a very particular data set and then, in the second half, details the precise construction of this data set. The second half details all the ad hoc pragmatic engineering choices necessitated by the construction of an extensive data set without human supervision. Knowing the engineering choices and their limitations feels necessary for interpreting the analysis of the resulting data set, which validates these pragmatic engineering choices as not unreasonable. "

Response R1.1: Thank you for this helpful suggestion. We followed your suggestion closely and added a new Section 2 called "ML framework overview", wherein we provide an overview of our LLM pipeline. Upon reading your comment, we realized that the original organization made it hard to interpret the descriptive results, as the engineering choices were relegated to the Method section at the end of the paper. Unfortunately, the editorial convention at *Nature Communications* is to have the Method section at the end of the paper (even after the Results and Discussion section), which can sometimes make it a bit more challenging to interpret the results.

We have therefore reorganized the manuscript to strike a balance between providing a methodological overview while also ensuring that we adhere to the editorial conventions of the journal. Specifically, we added a dedicated subsection before the start of the Results that provides an overview of our end-to-end pipeline, including the core engineering choices and their implications for interpretation. Our new overview also points readers directly to the validation evidence and introduces new stagewise analyses that quantify coverage across pipeline steps, so that the descriptive findings can be read in light of where information enters the process. We hope this revised structure makes it easier for readers to interpret the descriptive patterns with the necessary methodological context. Nevertheless, we still followed the editorial guidelines at *Nature Communications* and kept the full technical details in the Methods and Supplementary Materials. If you or the editor prefer a different placement, we would be happy to adjust accordingly.

To ease review, we copy below our new Section 2 ("ML framework overview") that we added to address your comment:

"ML framework overview

Our end-to-end ML framework operates as follows (Fig. 1b; see the Methods and the Supplementary Materials for full technical details). For each company–year (i.e., each company observed in a given year), we collect the corresponding annual and sustainability reports (as PDFs), extract and clean the text from the PDFs, split the text into overlapping chunks, and index the chunks in a vector database. Within each company–year, we then query the indexed text separately for each of the 501 ESG indicators, retrieve and re-rank candidate chunks, and use a state-of-the-art LLM to extract the requested numeric value (or abstain if absent). Finally, we standardize units and currencies and store the resulting output in a structured format (Supplementary Fig. S4). We apply this framework to STOXX Europe 600 index constituents (as of 2023) over the 2014–2023 period. Key methodological limitations are discussed in the Supplementary Materials S4.

We validated the outputs against (i) a proprietary benchmark dataset and (ii) expert human annotations on a subset. The agreement is strong against both comparisons (Supplementary Fig. S1–S2), which implies that the generated dataset is reliable for the descriptive analyses below. The full validation design and error analysis are described in Methods (Section "Validation").

While our framework enables large-scale, automated ESG indicator extraction, the reliability of its outputs depends on the quality of extraction at each stage of the pipeline. We therefore quantify how observations progress through each stage of the ML pipeline and report the results at both the company–year level and the indicator–company–year level, which allows us to identify at which stage information enters—or drops out of—the process (Supplementary Fig. S6). Building on this

decomposition, an indicator may fail to be retrieved for two reasons: (i) it may not be disclosed (e.g., due to non-materiality or because the company reports a different indicator or uses a different reporting variant), or (ii) the extraction may fail (e.g., due to ambiguous reporting). To assess whether the extraction may further vary across indicators, we additionally report per-indicator disclosure detection and standardization rates (Supplementary Table S6)."

Comment R1.2: *"- The positive enthusiasm about emerging methodological opportunities that readers feel throughout the paper is a key strength that should not be lost by endless stressing of non-trivial limitations, provisos, and qualifications. However, showing awareness of the significant limitations at several key points would significantly strengthen the paper's contributions in clarity and impact. Let me limit my suggestion to the framing and key methodological aspects, more clearly carving out the contributions of the current data and analysis."*

Response R1.2: Thank you. In response, we have revised the Discussion to more clearly delineate what our data and analyses support. Specifically, we emphasize that our contribution lies in providing large-scale descriptive evidence on ESG disclosure (transparency) and extracted quantitative indicators (performance) within the STOXX Europe 600 reporting population. We clarify that the analyses are descriptive and do not establish causal relationships, and we explicitly distinguish between empirical patterns and potential interpretations. Where readers might otherwise infer stronger claims—for example regarding drivers of trends or policy implications—we have added concise statements to clarify the intended scope. Accordingly, the revised Discussion now includes the following dedicated limitations paragraph (copied below for ease of review):

> "Our work is subject to the following limitations. First, the observed patterns are consistent with multiple causes, yet we refrain from such causal claims and interpret the results as descriptive, opening avenues for future research using our dataset. Second, as we apply the ESRS framework retrospectively to reports published before ESRS became mandatory, documented data gaps may partly reflect differences in the reporting frameworks under which companies report. Transparency scores should therefore be interpreted as alignment with current ESRS requirements. However, ESRS are largely based on previously established frameworks, especially GRI, and thus overlap substantially in underlying indicators, making our findings relevant beyond ESRS-specific requirements. Third, sustainability reports are self-reported and, during our sample period, subject to limited assurance. While our framework can detect what is disclosed, it cannot identify strategic non-disclosure. Nevertheless, such selective disclosures should be mitigated through future regulations, including ESRS, with mandatory audits of such data being rolled out. Importantly, outright falsification of financial or ESRS reports constitutes a legal offense under existing capital market regulations, which may deter systematic data manipulation. Moreover, non-retrieval of an indicator can reflect either non-disclosure or imperfect extraction (e.g., due to ambiguous information in the report); hence, absence of an extracted value does not necessarily represent the absence of reporting. Fourth, our sample comprises the 600 largest listed European companies, placing it at the upper tail of company size, visibility, and institutional coverage. Our findings may not generalize to smaller or private companies, or to non-European markets where regulatory pressure and stakeholder expectations differ substantially. Nevertheless, our ML framework is scalable, enabling future research to extend the analysis to other regions and company populations. Fifth, the accuracy of the RAG pipeline depends on the structure, clarity, completeness, and consistency of the corporate reports analyzed. Variations in data quality—such as missing or ambiguous information—can affect extraction accuracy, and LLM-based extraction may be prone to errors arising from hallucinations and the opaque reasoning processes inherent in LLMs. We thus validated extracted values against two external datasets (see Supplementary Fig. S1) to confirm the reliability of our framework."

At the same time, we were careful not to dilute the paper's central contribution. Rather than repeatedly qualifying results throughout the main text, we consolidate methodological limitations and their implications in a dedicated Supplementary section (new Supplementary Materials S4). This allows us to transparently document boundary conditions, data construction constraints, and interpretive caveats, while maintaining a clear narrative in the main manuscript.

Comment R1.3: *"- First, in terms of framing and novelty, a slight straw man feeling of overstated novelty in the sense that the 2024 ESRS standard is now mandatory and includes the Sustainability Reporting in a Digital Format (ESEF) with standardized and machine-readable data as reported directly by each company. This makes the papers' approach focus only on historic, legacy data (rather than current data), and most of the issues as reported in the paper should no longer apply. In addition. While the current paper's focus differs, there is endless related research on technically highly advanced LegalTech, FinTech, and AI approaches in the literature. E.g., publicly traded companies deposit any contract in platforms like EDGAR (US), Sedar (Canada), or similar platforms, which are widely used in AI/ML/NLP research, and many data sets and advanced document processing pipelines are available. Here, critically evaluating the quality of information extraction is central. And the state of the art in AI technology is progressing fast, but it is still far from perfect."*

Response R1.3: Thank you. We appreciate the opportunity to clarify the institutional context of ESRS digital reporting and to further delineate our contribution relative to existing research. We would like to address two aspects raised in your comment: (i) the current state of ESRS reporting infrastructure, and (ii) how our work relates to prior AI-based research on regulatory filings.

(i) The current state of the ESRS digital reporting infrastructure

We would like to put the premise underlying the first part of this comment ("*the 2024 ESRS standard is now mandatory and includes the Sustainability Reporting in a Digital Format (ESEF) with standardized and machine-readable data*") into perspective, because we believe it is not fully in line with the current state of the ESRS digital reporting infrastructure.

First, ESRS reporting has indeed become mandatory for the first cohort of firms for fiscal year 2024 in EU member states; yet, this is only true for those EU member states that have transposed the CSRD into national law—not for others, including large economies such as Germany. Further, the application of the European Single Electronic Format (ESEF) to ESRS sustainability statements has been explicitly postponed. At present, ESRS sustainability information is not required to be reported in a standardized, machine-readable XBRL/iXBRL format comparable to ESEF financial statements. Consequently, companies' ESRS disclosures are still predominantly provided in narrative, PDF-based formats, often embedded in management reports. The central premise that firm-level, standardized, machine-readable ESRS data are already available is therefore incorrect. Specifically, as communicated by EFRAG (https://www.efrag.org/en/sustainability-reporting/esrs-workstreams/digital-reporting-with-xbrl), *"EFRAG has been requested by the European Commission (EC) to develop the digital XBRL taxonomies needed to digitise the sustainability statements in an electronic format. [...] The XBRL taxonomies are the basis for the European Securities and Markets Authority (ESMA) to develop Regulatory Technical Standards (RTS) for tagging the sustainability statements. The final RTS will be adopted by the EC by way of a Delegated Act (DA), through the amendment of the Commission Delegated Regulation (EU) 2019/815 on the European Single Electronic Format (ESEF). Digital tagging will not be mandatory for companies until the EC adopts the XBRL taxonomy as part of the ESEF RTS that will be prepared by ESMA."*

Second, the European Single Access Point (ESAP)—which is intended to provide centralized, harmonized, and machine-readable access to sustainability reporting data—is not yet live and will only be rolled out gradually. Its full functionality will not be available until at least mid-2027, and even that timeline remains uncertain. Whereas Regulation (EU) 2023/2859 (the ESAP Regulation; published in the Official Journal 20 December 2023, and in force 9 January 2024), requires that ESMA must establish

ESAP by 10 July 2027, full functionalities remain to be implemented (see above). Furthermore, the "Stop-the-Clock Directive" (Directive (EU) 2025/794 of the European Parliament and of the Council of 14 April 2025, published 16 April 2025) postpones the application dates for sustainability reporting obligations for "wave 2" and "wave 3" firms, further delaying population of the ESAP, once established, with a meaningful cross-section and time series of ESRS sustainability statements. Given the current political environment, in which sustainability reporting is increasingly framed as an example of costly regulatory overreach that allegedly undermines EU competitiveness, and in which even transposition of the CSRD is not achieved in several EU member states (see above), further delays or scope reductions are a realistic possibility rather than a remote risk.

Third, even once ESEF-based digital tagging is introduced and ESAP becomes operational, they will not make standardized, machine-readable data available retroactively for prior reporting years.

We thus have added the following clarification on the current state of the ESRS digital reporting infrastructure (see new Supplementary Materials S2.2):

> "Although ESRS reporting has become mandatory for the first cohort of firms for fiscal year 2024 in European Union member states that have transposed the CSRD into national law (European Parliament and the Council of the European Union, 2022), the digital reporting infrastructure for sustainability statements is still being phased in. In particular, the application of the European Single Electronic Format (ESEF; European Commission, 2019) to ESRS sustainability statements has been explicitly postponed, and ESRS sustainability information is therefore not yet required to be reported in a standardized, machine-readable XBRL/iXBRL format comparable to ESEF financial statements (European Financial Reporting Advisory Group [EFRAG]). Consequently, ESRS disclosures remain predominantly narrative and PDF-based, often embedded in management reports.
>
> Centralized access to sustainability reporting via the European Single Access Point (ESAP; European Parliament and the Council of the European Union, 2023) is also not yet live and will be rolled out gradually, with full functionality expected only in later stages of implementation. Moreover, even once digital tagging and centralized access are fully implemented, these infrastructures are forward-looking and do not automatically make standardized, machine-readable data available retroactively for prior reporting years. This motivates the continued value of systematic extraction and validation approaches for constructing longitudinal baselines for pre-CSRD and early-transition disclosures."

(ii) How our work differs from prior AI-based research on regulatory filings

Regarding the second part of your comment, we appreciate the opportunity to cite AI-based research on regulatory filings more extensively and to clarify how our work differs from that literature. We have therefore expanded the Introduction to more explicitly position our contribution relative to prior LegalTech, FinTech, and NLP research. While a substantial body of work applies NLP to regulatory filings (e.g., EDGAR, SEDAR) and has developed technically sophisticated document processing pipelines, the primary focus of this literature is typically on text classification tasks—such as sentiment analysis, readability, tone, risk disclosure, or information content. These approaches generally operate on unstructured narrative text and aim to derive latent textual features or predictive signals. In contrast, our task centers on retrieval and structured information extraction: we systematically identify and extract predefined quantitative ESG indicators aligned with a regulatory taxonomy (ESRS) from lengthy and heterogeneous reports. This requires linking indicator definitions to dispersed textual and tabular content, resolving units and reporting scopes, and standardizing outputs into machine-readable, panel-structured data. The methodological challenges therefore differ from those in text classification, as the emphasis lies on precise indicator-level extraction (and validation) rather than on aggregate, document-level textual characteristics. Because of this reason, existing LLM pipelines are also not directly transferable.

We thus have added the following statement to the Introduction to position our study relative to prior LegalTech/FinTech and NLP work on regulatory filings across the globe:

> "Fourth, while there is a large body of research in finance, accounting, and legal studies using natural language processing to analyze regulatory filings (see overviews in Loughran and McDonald, 2016, and Bochkay et al., 2023), primarily for the purpose of text classification (e.g., sentiment analysis, readability analysis), monitoring ESG performance is a different task and requires the extraction of structured quantitative information."

We finally address your point about the rapidly evolving—but still imperfect—state of AI-based document processing in a dedicated Supplementary section, where we motivate why systematic validation and error analysis remain central (see new Supplementary Materials S4). In this context, we have added additional validations, including diagnostics to assess how observations progress through each step of the extraction process and where potential information loss may occur. These diagnostics help distinguish between non-disclosure and extraction-related errors. Importantly, our validation analyses indicate strong agreement with benchmark datasets, suggesting that systematic extraction errors are rare and unlikely to drive the reported patterns. We refer to our detailed responses above (see Response R1.4 after the next comment below) for a comprehensive description of these additions and how they strengthen our validation.

Comment R1.4: *"- Second, the ultimate dataset is a combination of selection bias on the population of included organizations (so the population is one set of STOXX 600 companies rather than whole sectors or industries, or continents), the success of the crawling strategy (e.g., not even all annual reports were included according to Fig. S3), the success of the OCR/PDF text extraction (this is never 100% or may be scrambled), the choice of the chunker (with very brief passages, which may work well for some but not all indicators), the success of the information extraction strategy (with just one prompt for each indicator), the success of the data normalization strategy (which may appear in many units and variants), etc, etc. Anything that looks like a simple problem turns out to be an imperfect extraction in reality, once you start measuring data (extraction) quality against ground truth. E.g., think of sentences mentioning both last year's and this year's numbers, or report in relative gains, or think of complex table extraction or getting numbers from images. As a result, the final data is the combination of the real-world data and a range of extraction errors (which we normally very carefully quantify against human reference data). With any analysis of this data, we must be aware that we are underreporting, which can result in selection bias. Moreover, the imperfection in the processing may not be equally distributed over variables, which can explain some of the observed variance."*

Response R1.4: Thank you for this thoughtful comment. Your comment prompted us to clarify and foreground our empirical validation strategy more explicitly in the manuscript, so that readers can more clearly assess the robustness of the machine learning framework in this application. In general, large-scale information extraction from unstructured documents is a well-established application area in machine learning (Grishman, 2019; Xu et al., 2024; Lavelli et al., 2008). In this literature, rigor is achieved through structured pipeline design combined with systematic empirical validation against independent reference data. Our study follows this framework. The dataset is constructed using a retrieval-based extraction architecture with controlled prompting and standardized postprocessing, and its quality is evaluated through rigorous benchmarking procedures consistent with best practice in machine learning (Grishman, 2019; Xu et al., 2024; Lavelli et al., 2008).

In our analysis, we empirically demonstrate that the extracted results are sufficiently reliable (see Methods, "Validation"). To this end, we follow established best practice in machine learning (Grishman, 2019; Xu et al., 2024; Lavelli et al., 2008) and implement a rigorous validation strategy, which we now present more prominently in the manuscript, including in Section 2 ("ML framework overview"). Specifically, we rely on two complementary validation approaches:

First, we compare our extracted values with a large proprietary ESG dataset from LSEG Refinitiv (London Stock Exchange Group, 2025). This comparison enables a broad, large-scale correspondence assessment across firms and indicators and therefore allows us to assess whether our framework aligns, at scale, with an established external data source.

Second, we constructed a human-annotated validation set directly from the underlying reports. This benchmark is necessarily smaller because manual annotation of sustainability reports is highly time-intensive, but it provides a direct reference point for evaluating extraction quality at the level of the source documents themselves. To assess the reliability of the manual benchmark, we additionally performed independent annotation and inter-rater agreement analysis, which indicated very high agreement across both annotated subsets (subset 1: ICC = 0.978, 95% CI [0.968, 0.985]; subset 2: ICC = 0.998, 95% CI [0.997, 0.998]).

Across both benchmarks, the extracted values track the reference data closely. For the Refinitiv comparison, the estimated correlation is 0.885 ($p < 0.001$), with an adjusted R^2 of 0.91; for the human-annotated benchmark, the estimated correlation is 0.885 ($p < 0.001$), with an adjusted R^2 of 0.93. *Hence, we find strong agreement between our LLM-based analysis and both benchmark datasets.*

To address your comment, we now foreground the empirical validation more prominently in the analysis (see our new Section 2, "ML framework overview"), so that readers can assess the strength and limitations of our LLM analysis. We also revised the manuscript to make the scope conditions of the dataset and the sources of incompleteness more transparent. Specifically, Section 2 now defines the population explicitly as STOXX Europe 600 constituents as of 2023 and clarifies that all empirical findings are conditional on this reporting frame and on the availability of public annual and sustainability reports. This framing is now introduced before the Results section so that readers encounter the population definition, data construction process, and validation strategy before interpreting the descriptive findings. We also expanded the descriptive reporting by sector and country across years in the revised Results section, and added Supplementary Fig. S5 (copied below for ease of review) to make the composition of this reporting frame explicit.

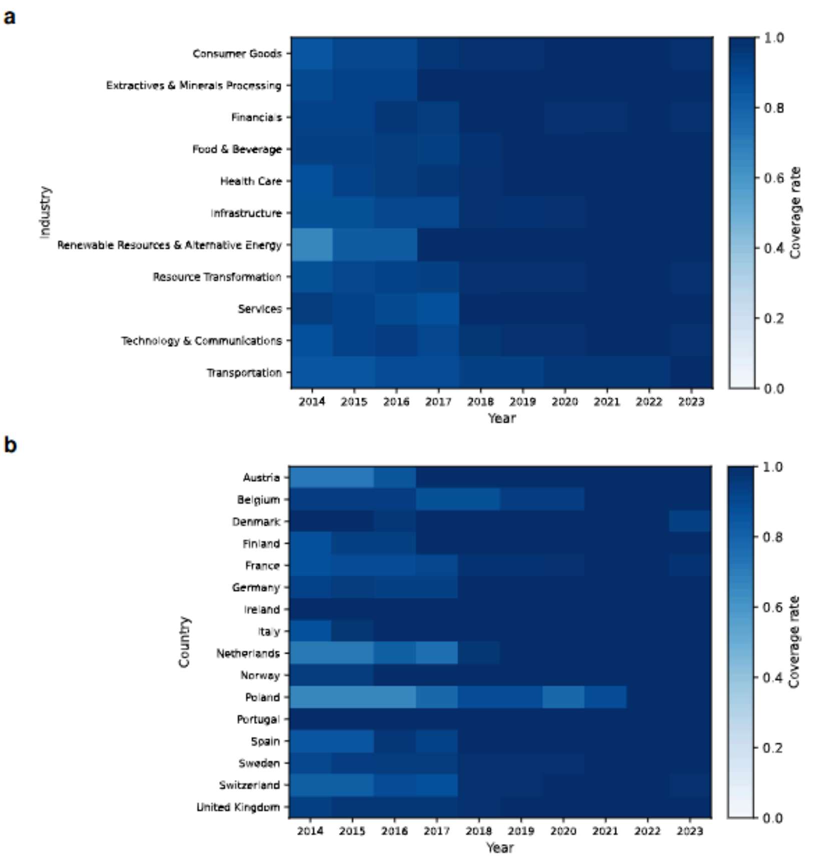

Fig. S5. Scope and annual coverage. a, Coverage by industry and year. Industries are categorized according to the Sustainable Industry Classification System (SICS), which was developed by the Sustainability Accounting Standards Board (SASB) to group companies into sectors with comparable exposure to sustainability-related risks and opportunities [37]. **b**, Coverage by country and year. Coverage is computed at the company–year level: for each STOXX 600 firm and year in the population table, a company–year is counted as covered if at least one report (e.g., annual report and/or sustainability report) is publicly available and included in our corpus for that company–year. Cells summarize the share of covered company–years within (**a**) each industry–year and (**b**) country–year. Coverage is high across both industries and countries and increases over time, converging to near-complete coverage for most industries and countries from roughly 2018 onward. Lower coverage is concentrated in the early years and in a small subset of industries and countries, consistent with more limited public availability of reports in the earlier part of the sample. Overall, the figure indicates broad and increasingly comprehensive coverage of STOXX Europe 600 firm-years in the corpus over the study period.

We further revised the manuscript to make the main sources of incompleteness more transparent. Specifically, we emphasize that cases where the pipeline does not retrieve an indicator can arise because of either non-disclosure or imperfect extraction; accordingly, non-retrieval does not necessarily represent the absence of reporting. To support this distinction empirically, we added pipeline coverage diagnostics that report coverage at successive processing stages—report availability, text extraction, chunking, indicator extraction, and standardization—both at the company–year and at the indicator–company–year level (new Supplementary Fig. S6; copied below). At the company–year level, reports were retrieved for 5,749 of 6,000 observations (95.8%), and text extraction and chunking were successful for all retrieved reports. At the indicator–company–year level, most missingness arises upstream because indicators are not detected as disclosed (67.7% of observations), whereas conditional on disclosure the pipeline successfully standardizes a numeric value for 88.6% of observations.

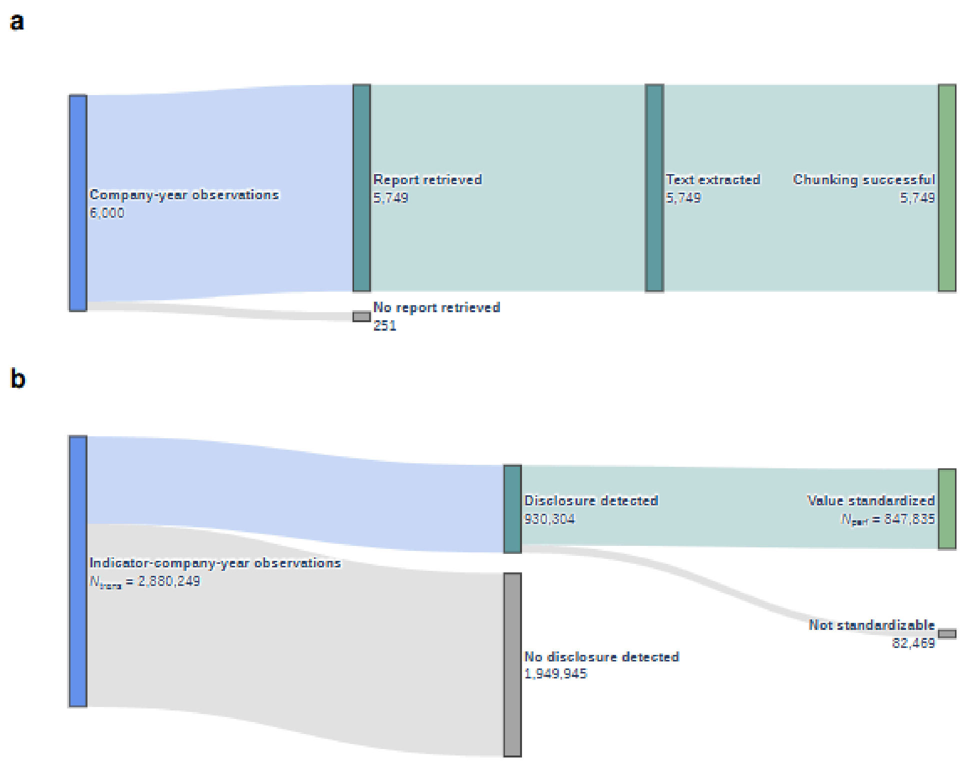

Fig. S6. Pipeline coverage analysis. a, Coverage of company-year observations across the end-to-end processing pipeline (successful report retrieval, text extraction, and chunking). Of all company-year observations under analysis (i.e., STOXX Europe 600, 2014–2023), 95.8% successfully pass all three stages and meet conservative minimum-quality gates designed to ensure substantive textual content (minimum extracted text length: 5,000 characters; minimum indexed chunks per vector store: 50). **b**, Coverage of indicator-company-year observations conditional on successful chunking, tracing indicator extraction from text, and subsequent normalization to a standardized, numeric representation. Among disclosed indicators, 88.6% yield a valid value that can be expressed in the EFRAG-specified unit after postprocessing.

To address the possibility that extraction difficulties are unevenly distributed across variables and could therefore contribute to observed variance across indicators, we additionally report indicator-level disclosure detection and standardization rates (new Supplementary Table S6), which make heterogeneity in extractability explicit. Taken together, our revised presentation of the validation evidence, population-level transparency, pipeline coverage diagnostics, and indicator-level extractability analyses are intended to make the main sources of uncertainty more explicit and to assess their likely importance empirically. We hope these revisions strengthen the manuscript by allowing readers to interpret the resulting dataset, its scope conditions, and its remaining limitations more transparently before drawing conclusions from the downstream analyses.

Comment R1.5: *"- Third, while the exploratory data analysis is interesting, a more critical discussion and awareness of limitations could be possible. For example, there are many potential confounding effects*

both in the data and outside the explicit variables. A more critical discussion of alternative explanations and what exact hypothesis the data suggests would strengthen the analysis. E.g., if a recency effect is observed, due to more reporting or better extraction quality, or a size effect, subsequent analysis could be sampled to compensate for this effect, allowing for the analysis of the independent contribution of other factors or indicators."

Response R1.5: Thank you for this helpful comment. Let us briefly summarize how we improved our paper before elaborating on the key findings from our new analysis: (i) In the revised Discussion, we strengthen the exploratory analysis by stating more explicitly which hypotheses the descriptive patterns suggest and what interpretive scope they support, providing a more critical discussion of the limitations. (ii) In addition, we conduct robustness checks to assess whether temporal patterns could be mechanically driven by recency-related changes (disclosure incidence or document-to-text quality). We also include more restrictive controls in our existing analyses to strengthen our inferences. (iii) Finally, in the spirit of this comment, we estimate multivariate regressions of company–year transparency to assess whether temporal patterns persist after accounting for observable firm-level heterogeneity, including firm size. Below, we discuss each point separately and detail how we improved our manuscript in response:

(i) Hypotheses and interpretive scope:

In our revised Discussion, we strengthen the discussion and awareness of limitations, articulate more clearly what specific hypotheses are suggested by the exploratory patterns, and broaden the critical discussion of alternative explanations and potential confounding influences—both in the underlying data and in the broader reporting environment—to clarify what can (and cannot) be inferred from the descriptive evidence. For ease of review, the revised limitations paragraph is copied in our Response R1.2.

(ii) Recency effects:

To assess whether temporal patterns could be driven by expanding disclosure coverage or improvements in document-to-text quality over time, we run two robustness checks: (a) we re-estimate temporal trends of ESG performance within the subset of constant reporters for each indicator, holding the reporting incidence fixed, and (b) we re-estimate transparency and performance trends on a subset of company–year observations with high document text quality, minimizing time-varying extraction noise. In both analyses, the temporal patterns remain consistent with the main analysis, indicating that our main findings are not primarily attributable to recency-related disclosure expansion or document quality improvements.

a. We re-estimate year-to-year trends within the subset of constant reporters for each indicator, i.e., firms that report the indicator in every year they are observed (see new Supplementary Fig. S18–S20, copied below for ease of review). The resulting trends closely mirror those in the full sample, consistent with our existing early-adopter versus full-sample comparison.

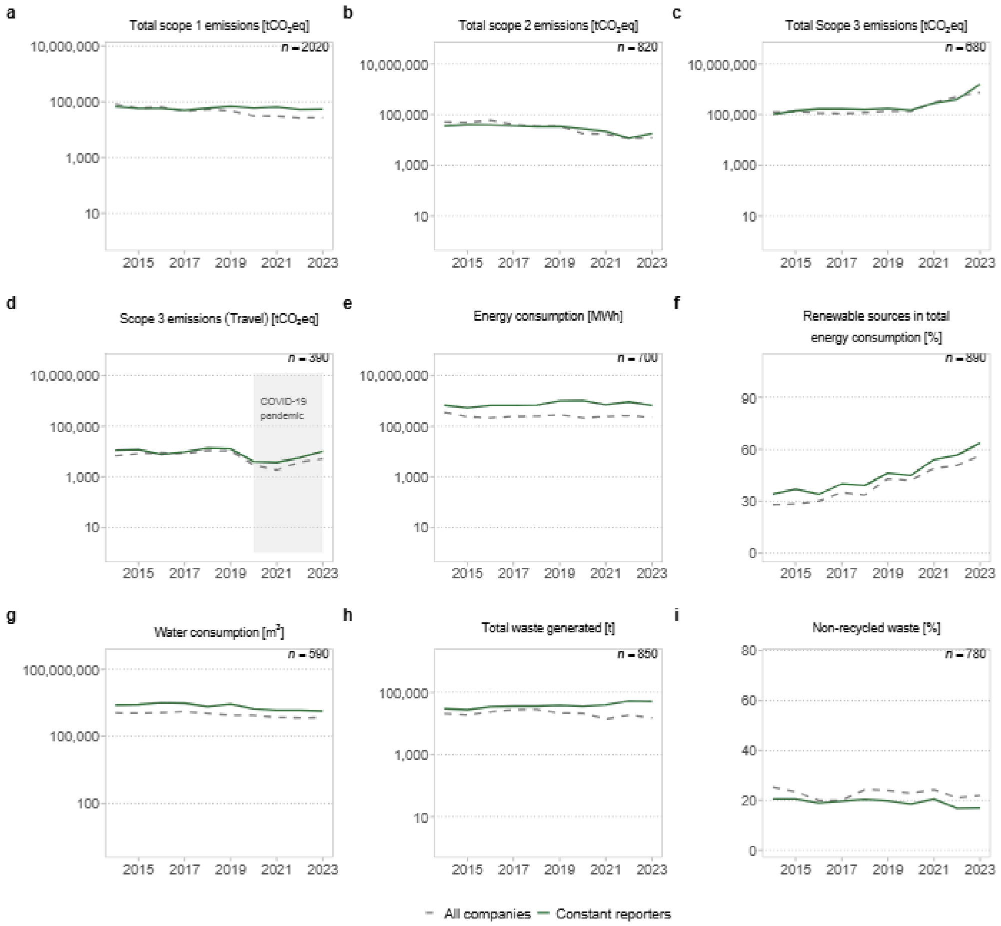

Fig. S18. Environmental indicators (median) for constant reporters and full sample. Here, we compare medians for environmental indicators between constant reporters,i.e., companies that report on a given metric for all years within the sample period, and our full sample. We report the number of company–year observations for constant reporters in the upper-right corner of each panel. In this figure, n refers to the number of company–year observations of constant reporters. Note that logarithmic axes are used to better visualize different orders of magnitude. For indicators recorded in percent, we use linear scales. Although this subset is more restrictive, the results closely mirror our findings for early adopting companies and the full sample.

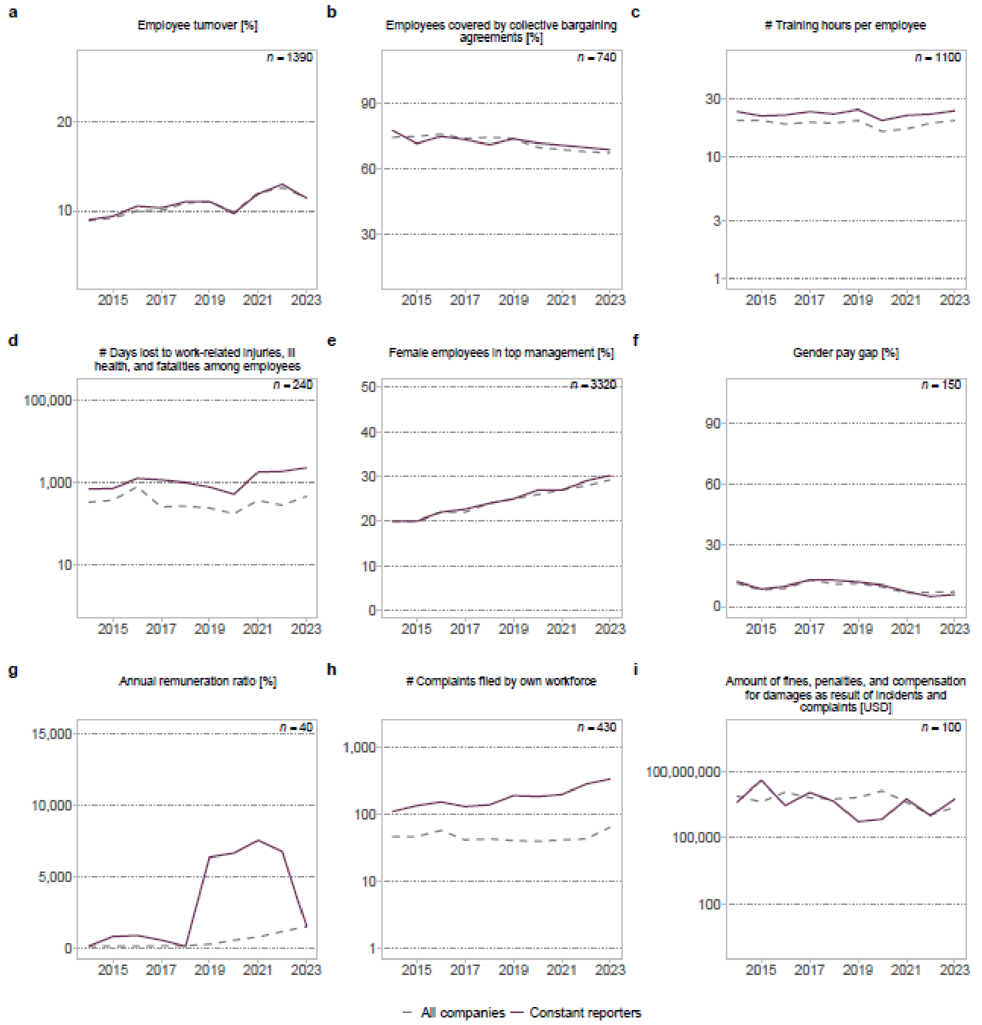

Fig. S19. Social indicators (median) for constant reporters and full sample. Here, we compare medians for social indicators between constant reporters, i.e., companies that report on a given metric for all years within the sample period, and our full sample. We report the number of company–year observations for constant reporters in the upper-right corner of each panel. In this figure, n refers to the number of company–year observations of constant reporters. Note that logarithmic axes are used to better visualize different orders of magnitude. For indicators recorded in percent, we use linear scales. Although this subset is more restrictive, the results closely mirror our findings for early adopting companies and the full sample, with the exception of the annual remuneration ratio, where the small sample of constant reporters ($n = 4$) limits the representativeness of the observed trend.

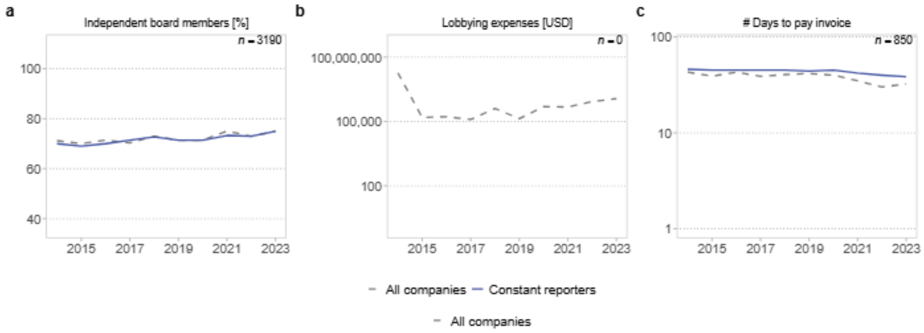

Fig. S20. Governance indicators (median) for constant reporters and full sample. Here, we compare medians for governance indicators between constant reporters (companies that report on a given metric for all years within the sample period) and our full sample. We report the number of company–year observations for constant reporters in the upper-right corner of each panel. In this figure, n refers to the number of company–year observations of constant reporters. Note that logarithmic axes are used to better visualize different orders of magnitude. For indicators recorded in percent, we use linear scales. Although this subset is more restrictive, the results closely mirror our findings for early adopting companies and the full sample. Our full sample does not contain any constant reporters for lobbying expenses.

b. We re-estimate the trends on the subset of company–year observations with high document text quality, defined as texts that, within each year, do not fall into the upper tercile on any of three proxies for text quality: newline density, boilerplate concentration, and the Fog index (see new Supplementary Fig. S7–S12, copied below for ease of revision). Patterns for both transparency and performance remain consistent relative to the full sample, indicating that the main results are not primarily driven by improvements in document quality over time.

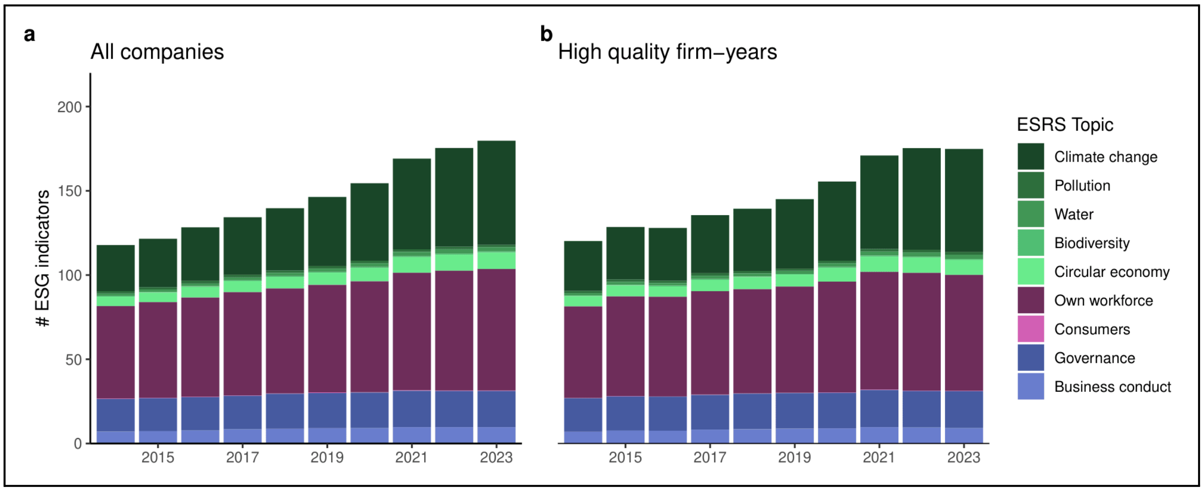

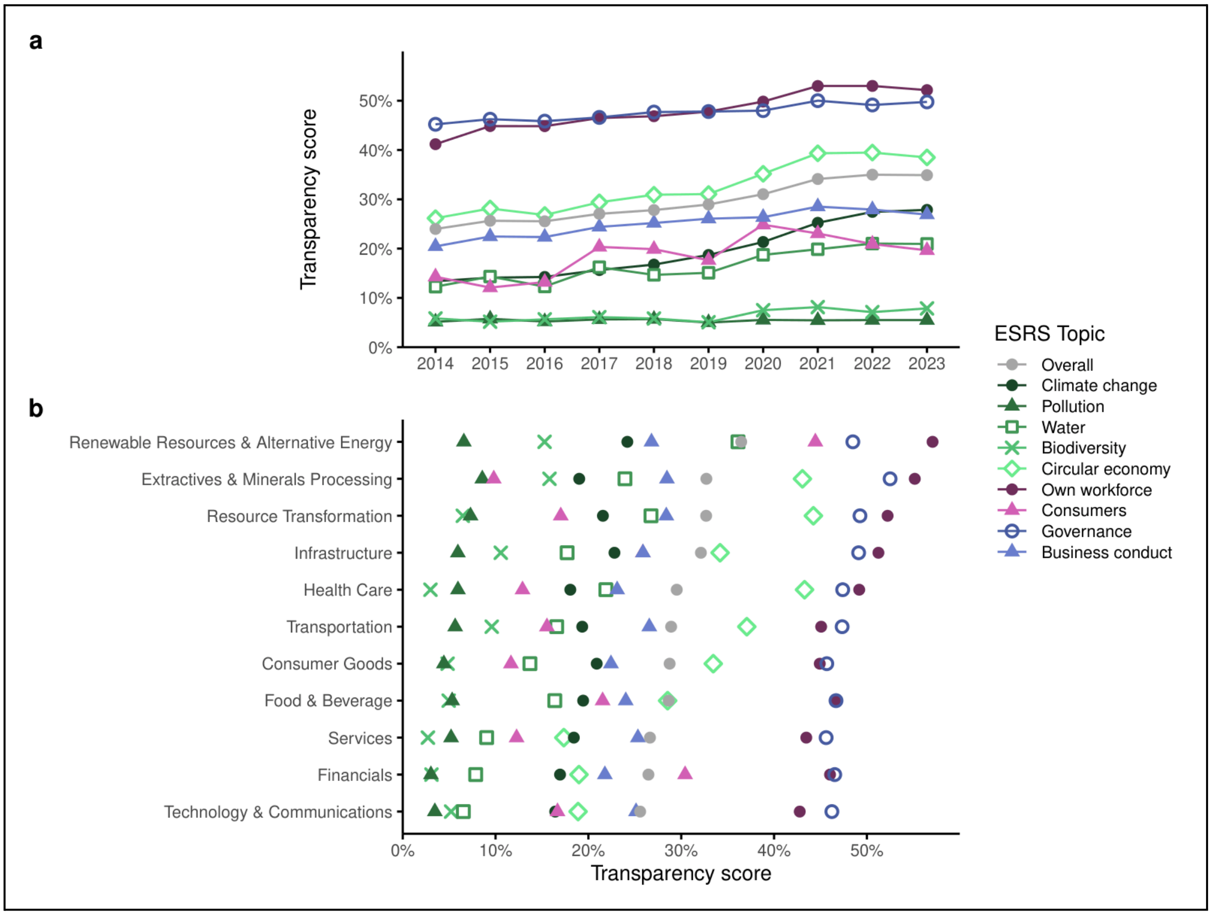

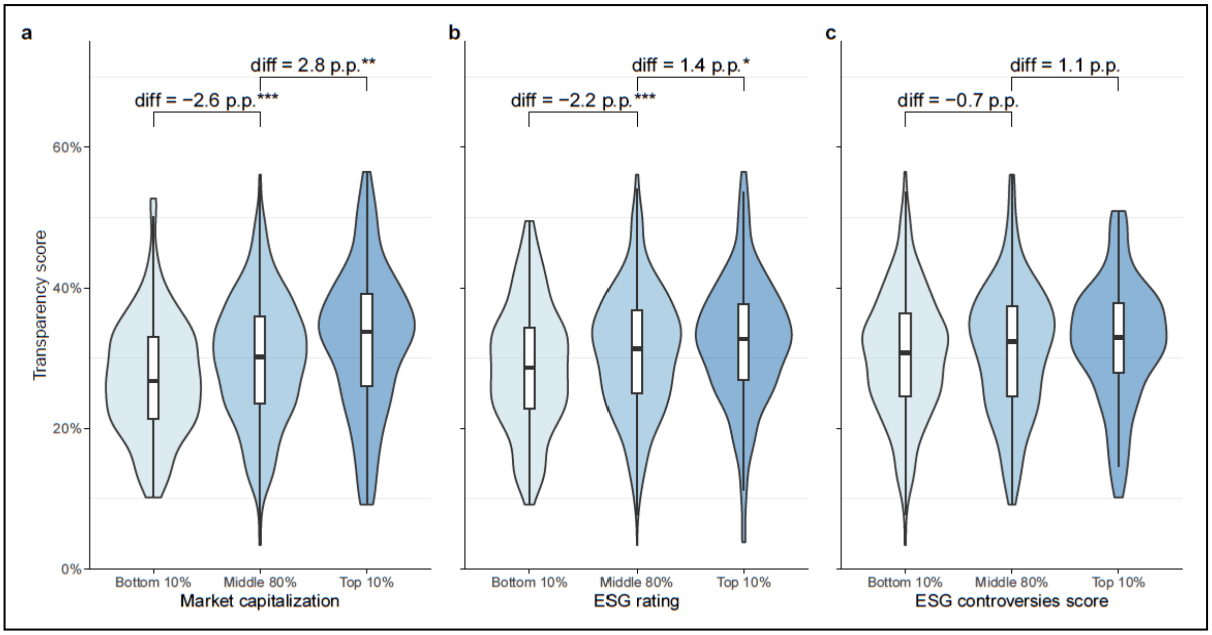
a
b
c
diff = 2.8 p.p.**
diff = −2.6 p.p.***
diff = 1.4 p.p.*
diff = −2.2 p.p.***
diff = 1.1 p.p.
diff = −0.7 p.p.
Transparency score
60%
40%
20%
0%
Bottom 10%
Middle 80%
Top 10%
Market capitalization
ESG rating
ESG controversies score

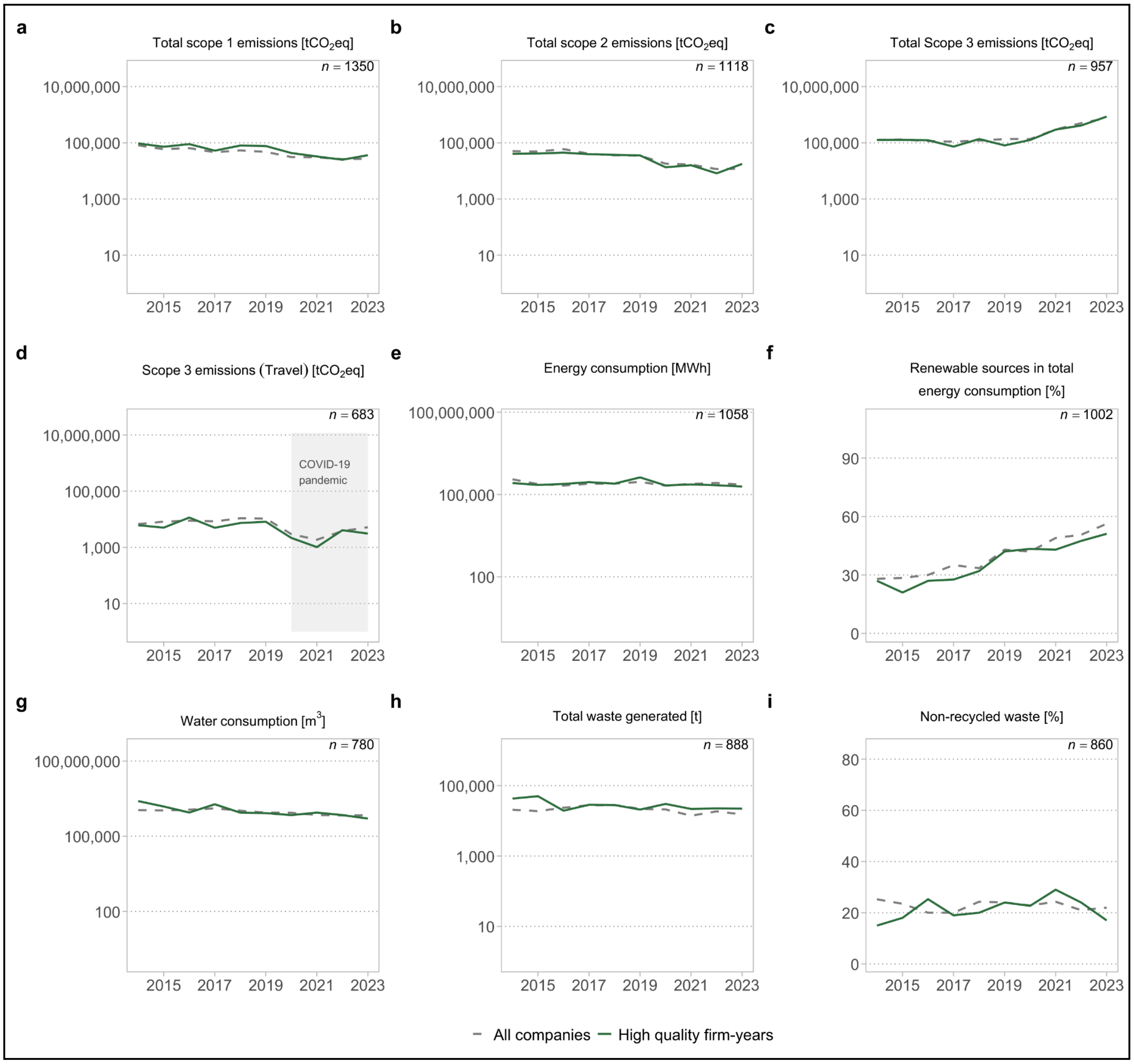
a
Total scope 1 emissions [tCO_2eq]
n = 1350
b
Total scope 2 emissions [tCO_2eq]
n = 1118
c
Total Scope 3 emissions [tCO_2eq]
n = 957
10,000,000
100,000
1,000
10
2015 2017 2019 2021 2023
d
Scope 3 emissions (Travel) [tCO_2eq]
n = 683
COVID-19 pandemic
e
Energy consumption [MWh]
n = 1058
100,000,000
100,000
100
f
Renewable sources in total energy consumption [%]
n = 1002
90
60
30
0
g
Water consumption [m^3]
n = 780
h
Total waste generated [t]
n = 888
i
Non-recycled waste [%]
n = 860
80
60
40
20
0
All companies
High quality firm-years

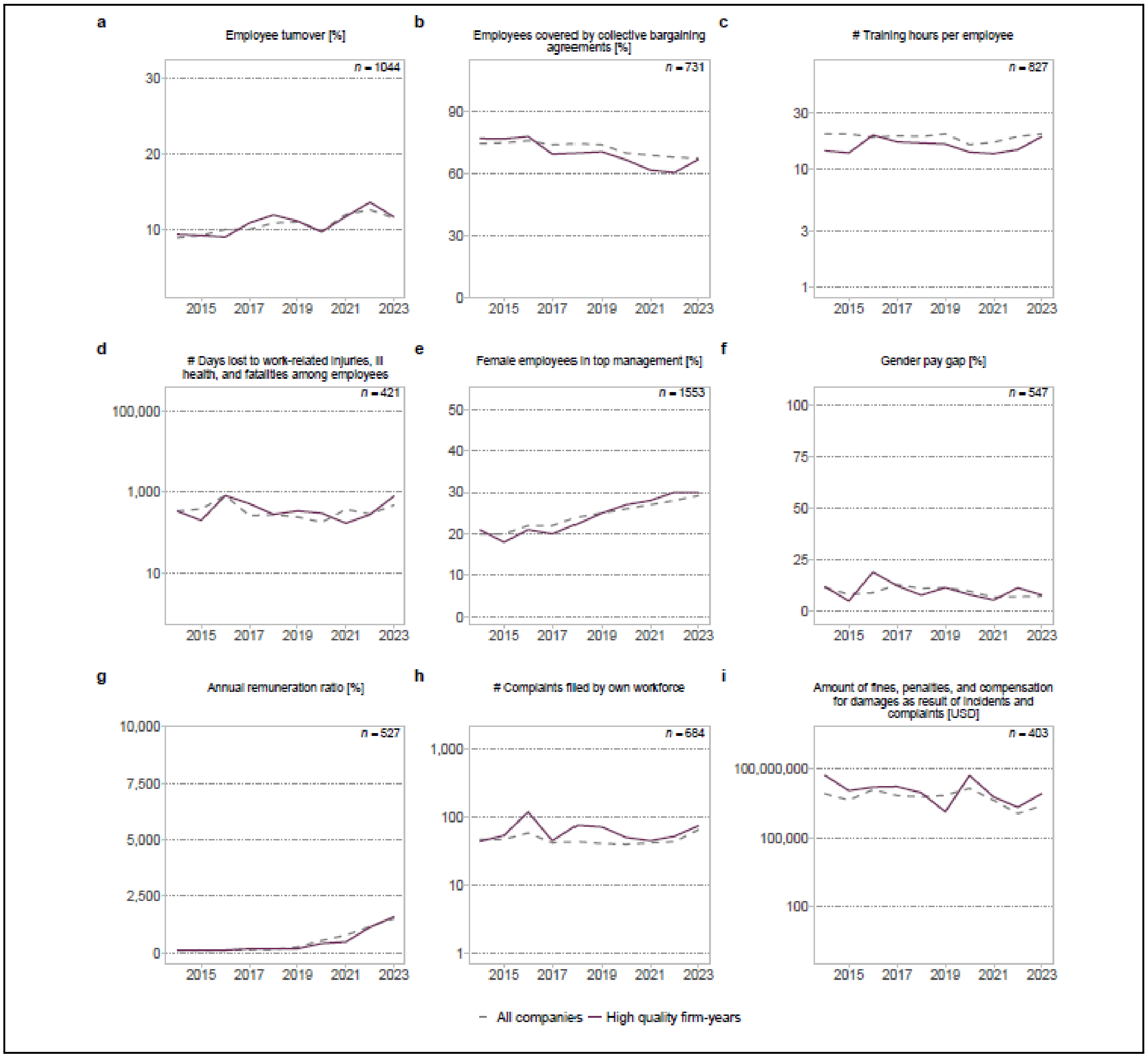

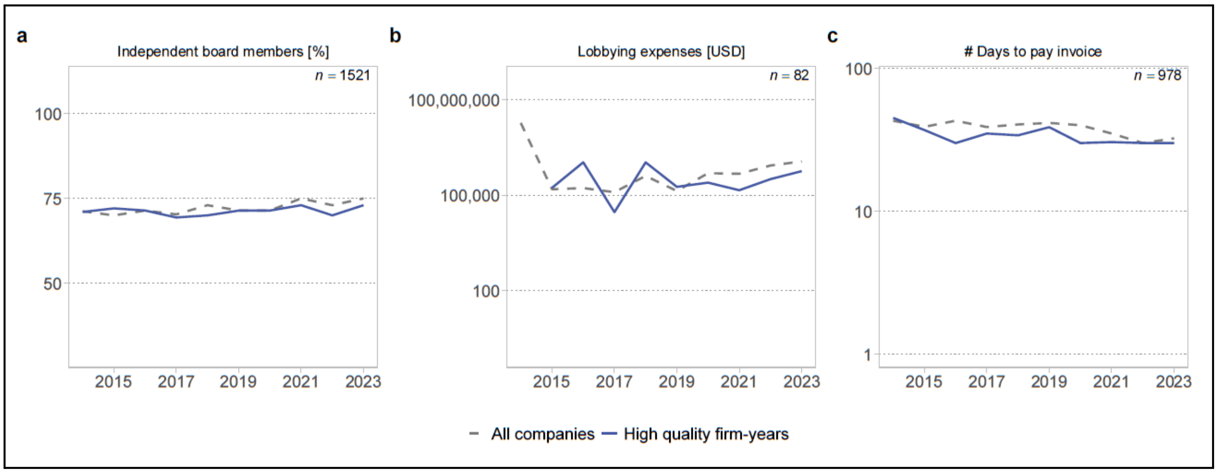

(iii) <u>Firm-level heterogeneity and observable drivers</u>:

To address the concern that the observed transparency patterns may be driven by observable firm-level heterogeneity rather than genuine temporal dynamics, and to strengthen the analysis of individual determinants, we conduct two complementary robustness analyses.

a. We re-estimate existing firm-year panel regressions with the transparency score as the dependent variable (see new Table 1). For each of our three main hypothesized determinants, log lagged market capitalization (proxying firm size), lagged ESG ratings, and lagged ESG controversies score, we construct top-10% and bottom-10% dummy variables calculated yearly and report results in a separate panel. We estimate six increasingly restrictive specifications: (i) without controls, or fixed effects, (ii) including the remaining two determinants as continuous controls, (iii) adding year fixed effects, (iv) adding sector fixed effects, (v) including year-by-sector fixed effects, and (vi) adding company fixed effects to absorb all time-invariant firm heterogeneity. Standard errors are clustered at the firm level. The results confirm the patterns documented in the univariate analysis. In Panel A, companies in the top 10% of market capitalization exhibit significantly higher transparency scores, while those in the bottom 10% exhibit significantly lower scores (Model 1). However, these effects attenuate and become statistically insignificant once we control for ESG rating and controversy exposure (Model 2), suggesting that the size-transparency association is largely explained by these other firm characteristics rather than by firm size per se. In Panel B, the ESG rating association is more robust: top-rated companies disclose significantly more across specifications (1)–(5), with the coefficient remaining significant even after including year-by-sector fixed effects (Model 5). The effect becomes insignificant only in the firm fixed effects specification (Model 6), indicating that the rating-transparency link is primarily a between-firm phenomenon. In Panel C, lower ESG controversy exposure is associated with significantly lower transparency in Model 1, while the top-10% effect is not significant. Across all remaining specifications, neither the top nor bottom group dummies are statistically significant, suggesting that the controversies-transparency association is already absorbed once the remaining determinants are included as controls. Overall, the large jump in adjusted R^2 from Model (5) to Model (6), from approximately 0.24 to 0.60, indicates that time-invariant firm heterogeneity accounts for a substantial share of variation in transparency, underscoring the importance of controlling for unobserved firm characteristics when interpreting cross-sectional transparency differences.

Table 1: Determinants of transparency

Panel A: Transparency explained by market capitalization	(1)	(2)	(3)	(4)	(5)	(6)
Constant	0.297***	0.195***				
	(0.003)	(0.012)				
Top-10% market capitalization	0.031***	0.016	0.022**	0.020*	0.026**	0.018
	(0.009)	(0.009)	(0.008)	(0.008)	(0.008)	(0.011)
Bottom-10% market capitalization	−0.039***	−0.003	−0.019*	−0.005	−0.021**	0.011
	(0.008)	(0.008)	(0.007)	(0.008)	(0.008)	(0.006)
Controls	No	Yes	Yes	Yes	Yes	Yes
Year FE	No	No	Yes	No	—	—
Sector FE	No	No	No	Yes	—	—
Year × Sector FE	No	No	No	No	Yes	Yes
Company FE	No	No	No	No	No	Yes
N	5431	4346	4346	4344	4344	4344
Adj. R^2	0.025	0.081	0.188	0.132	0.238	0.604

Panel B: Transparency explained by ESG rating	(1)	(2)	(3)	(4)	(5)	(6)
Constant	0.305***	0.036				
	(0.003)	(0.061)				
Top-10% ESG rating	0.037***	0.029***	0.024**	0.027***	0.022**	−0.006
	(0.007)	(0.007)	(0.008)	(0.007)	(0.007)	(0.008)
Bottom-10% ESG rating	−0.025***	−0.022**	−0.028***	−0.015*	−0.022**	−0.004
	(0.007)	(0.007)	(0.007)	(0.007)	(0.007)	(0.004)
Controls	No	Yes	Yes	Yes	Yes	Yes
Year FE	No	No	Yes	No	—	—
Sector FE	No	No	No	Yes	—	—
Year × Sector FE	No	No	No	No	Yes	Yes
Company FE	No	No	No	No	No	Yes
N	4609	4346	4346	4344	4344	4344
Adj. R^2	0.033	0.052	0.185	0.114	0.243	0.603

Panel C: Transparency explained by ESG controversies score	(1)	(2)	(3)	(4)	(5)	(6)
Constant	0.325***	−0.015				
	(0.005)	(0.062)				
Top-10% ESG controversies score	−0.006	0.001	−0.001	−0.001	−0.003	−0.002
	(0.007)	(0.006)	(0.006)	(0.006)	(0.006)	(0.005)
Bottom-10% ESG controversies score	−0.024***	−0.010	−0.009	−0.009	−0.008	−0.005
	(0.005)	(0.005)	(0.005)	(0.005)	(0.005)	(0.003)
Controls	No	Yes	Yes	Yes	Yes	Yes
Year FE	No	No	Yes	No	—	—
Sector FE	No	No	No	Yes	—	—
Year × Sector FE	No	No	No	No	Yes	Yes
Company FE	No	No	No	No	No	Yes
N	4565	4346	4346	4344	4344	4344
Adj. R^2	0.010	0.088	0.194	0.144	0.250	0.603

b. We estimate company–year panel regressions that interact top-10% and bottom-10% ESG rating group dummies with a linear time trend, allowing for differential transparency trajectories across rating strata (see new Supplementary Table S2). Models (2)–(4) additionally control for log lagged market capitalization and lagged ESG controversies exposure; Model (3) includes sector fixed effects, and Model (4) includes firm fixed effects. Standard errors are clustered at the firm level. Across all specifications, the linear time trend is positive and highly significant, confirming a steady increase in transparency over the sample period. The level differences between rating groups are consistent with the findings from Table 1: top-rated companies exhibit significantly higher transparency, while bottom-rated companies disclose significantly less. Crucially, the interaction terms point toward convergence. The “Top-10% × Time trend” coefficient is negative, indicating that the transparency advantage of top-rated companies diminishes over time, while the “Bottom-10% × Time trend” coefficient is positive, suggesting that lower-rated companies are catching up. These interaction effects are marginally significant in the unconditional specification (Model 1) but attenuate and lose significance as controls and fixed effects are added (Models 2–4), suggesting that the convergence pattern is partly explained by other firm characteristics evolving over time. In the firm fixed effects specification (Model 4), neither the level differences nor the differential trends remain significant, indicating that convergence is primarily a between-firm phenomenon, consistent with laggards increasingly adopting established disclosure practices rather than individual firms changing their relative position within the rating distribution.

Table S2: Transparency trends by rating group

	(1)	(2)	(3)	(4)
Constant	0.241***	−0.037		
	(0.006)	(0.058)		
Top-10% group	0.051***	0.036**	0.036**	0.006
	(0.013)	(0.013)	(0.012)	(0.012)
Bottom-10% group	−0.047***	−0.046***	−0.037***	−0.011
	(0.011)	(0.011)	(0.010)	(0.008)
Time trend	0.013***	0.013***	0.013***	0.014***
	(0.001)	(0.001)	(0.001)	(0.001)
Top-10% × Time trend	−0.004*	−0.002	−0.003	−0.003
	(0.002)	(0.002)	(0.002)	(0.002)
Bottom-10% × Time trend	0.003*	0.004*	0.003	0.002
	(0.002)	(0.002)	(0.002)	(0.001)
Determinant for top/bottom groups	ESG rating			
Controls	No	Yes	Yes	Yes
Sector FE	No	No	Yes	No
Firm FE	No	No	No	Yes
N	4,609	4,346	4,344	4,346
Adj. R^2	0.174	0.186	0.247	0.600

In sum, we believe that these revisions strengthen the critical discussion and awareness of limitations, while adding diagnostic evidence that the reported trends are unlikely to be primarily artifacts of disclosure expansion, extraction quality, or other observable drivers.

Comment R1.6: *“- The analysis makes a large number of engineering choices that are clearly documented and are each an entirely reasonable and pragmatic choice in themselves. The strength of this is that it enables novel analysis at a far larger scale, and far less human time and effort in annotation.*

This is a key strength that can accelerate research significantly. With a slightly more critical reporting showing more awareness of both the strengths and limitations of this methodological choice, the manuscript can make an essential contribution. A request for more critical reflection on the methodology may be unfair, as no paper using traditional methods will detail textbook-level explanations of basic methodology. When moving to a new "AI paradigm," the onus of accounting for the new methodology lies with those venturing into this new area. Perhaps undeservedly, this comes with a responsibility to very critically account for both strengths and limitations of the new methodology — just to fully inform the reader, and allow them to interpret the results and evidence on which these are based to their true value. Similar to the emerging standards on reporting sustainability efforts studied in the paper, we can only expect the highest levels of transparency and detail on the consequences of the combined limitations."

Response R1.6: Thank you. We have revised the manuscript to more critically and transparently contextualize our AI-based extraction pipeline and to substantiate its strengths and limitations with targeted robustness checks, so that readers can interpret the results appropriately. In particular, we have revised the manuscript (see revised Discussion section) to more explicitly highlight both the strengths—namely, scalability, systematic coverage, and reproducibility—and the inherent trade-offs of the pipeline. We clarify that the central methodological advantage lies in enabling large-scale, indicator-level extraction across thousands of reports, which would not be feasible through manual annotation alone. At the same time, we acknowledge that such scalability comes with potential limitations in precision and heterogeneity in extractability across indicators, and we now make this balance more explicit in the main text.

We have also carefully checked the Methods section to ensure that all key components of the LLM pipeline are fully documented. This includes preprocessing, indexing (including embedding), retrieval (including reranking and context expansion), generation (including model selection, parameter settings, prompting strategy, and abstention handling), postprocessing (EFRAG-aligned unit standardization), and validation protocols. The corresponding code for these components is provided in the Supplementary Files and will be made publicly available upon acceptance, along with documentation that allows the pipeline to be rerun and extended in future applications. This ensures transparency and reproducibility of both the extraction process and the downstream analyses.

To further strengthen the transparency and rigor, we have implemented the following improvements:

- To make the methodological foundations more transparent, we added a structured overview of the full pipeline to the main manuscript (see new Section 2; "ML framework overview"). This new section summarizes the task definition, data scope (STOXX Europe 600 population), preprocessing steps, retrieval and extraction strategy, validation design, and interpretive boundaries before presenting the substantive findings. By introducing these elements early, readers can better assess how the data are generated and what conclusions the evidence supports.
- We have added a dedicated Supplementary section that consolidates key methodological limitations and their implications for our AI-based extraction pipeline (Supplementary Materials S4). Specifically, we discuss the main challenges that can affect completeness and accuracy, including sensitivity of PDF parsing to complex layouts, retrieval limitations when relevant information is dispersed or expressed indirectly, ambiguity arising from heterogeneous indicator definitions and reporting scopes, challenges in attributing values to the intended reporting period, and the general risk of hallucinated outputs in LLM-based extraction. We also document the safeguards implemented to mitigate these limitations (e.g., overlapping chunks and context expansion, ESRS-aligned indicator descriptions in tailored prompts, explicit reporting period cues, and conservative abstention prompting).
- To further enhance transparency, we now include a structured reporting checklist for the LLM component based on GUIDE-LLM, a consensus-based reporting framework (see http://www.llm-checklist.com/llm-checklist.pdf). This checklist systematically documents model selection, prompting strategy, parameter settings, abstention handling, postprocessing, and

validation choices, thereby standardizing the disclosure of design decisions. and other key design choices in a standardized way. The completed checklist is provided in our public code repository.

- We now provide a structured, end-to-end summary of the pipeline with stage-wise coverage reporting (per company–year observation and per indicator–company–year), so readers can directly see where information can be distorted and how this may affect downstream analyses (new Supplementary Fig. S6):

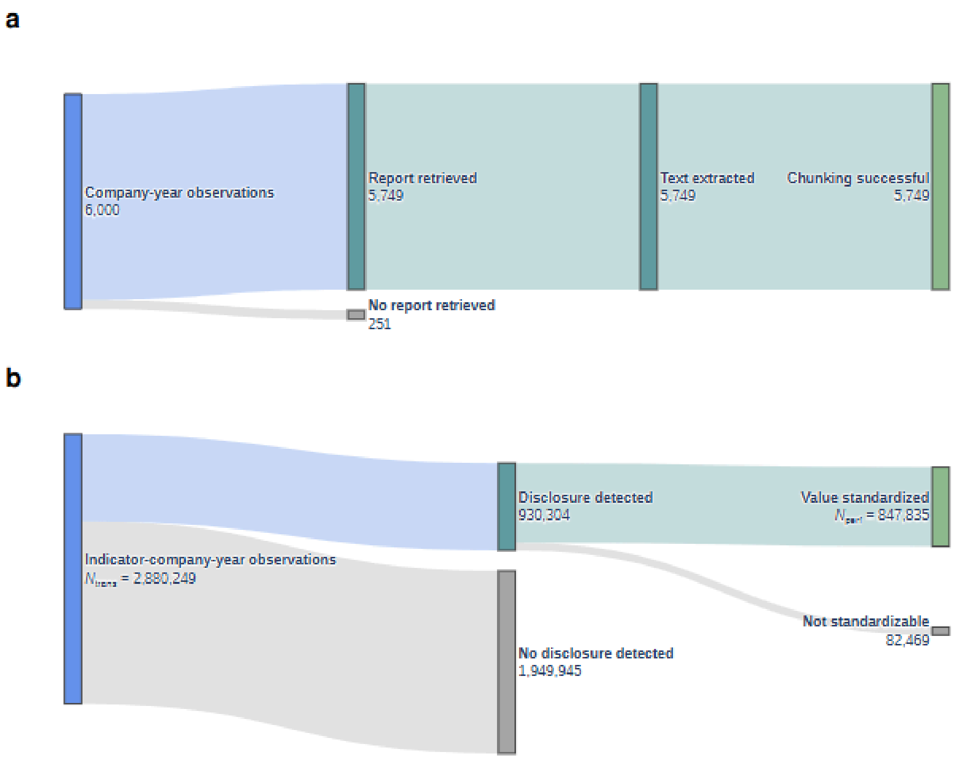

Fig. S6. Pipeline coverage analysis. a, Coverage of company-year observations across the end-to-end processing pipeline (successful report retrieval, text extraction, and chunking). Of all company-year observations under analysis (i.e., STOXX Europe 600, 2014–2023), 95.8% successfully pass all three stages and meet conservative minimum-quality gates designed to ensure substantive textual content (minimum extracted text length: 5,000 characters; minimum indexed chunks per vector store: 50). **b,** Coverage of indicator-company-year observations conditional on successful chunking, tracing indicator extraction from text, and subsequent normalization to a standardized, numeric representation. Among disclosed indicators, 88.6% yield a valid value that can be expressed in the EFRAG-specified unit after postprocessing.

Remarks on code availability

Comment R1.7: *"Just a bunch of csv files.*

Didn't check the raw download, nor run these through a standard pipeline from the literature.

The authors' processing reveals great effort and care (but also limited experience in large-scale document processing and information extraction in a technical field)."

Response R1.7: Thank you for reviewing the shared materials. We have expanded the repository documentation to describe the end-to-end pipeline (from downloaded reports to the released CSV files) and provide metadata for each CSV file. In addition, we provide the full set of raw processing scripts and analysis code required to reproduce the results presented in the manuscript. This includes the pipeline components used for preprocessing, indexing, retrieval, generation, postprocessing, and validation, as well as the scripts used to generate all tables and figures in the paper. We also include a comprehensive README file that documents prerequisites and software dependencies, configuration settings, and step-by-step instructions for rerunning the pipeline and reproducing the empirical analyses.

Importantly, our code is open-source to facilitate updates in future years. Researchers can extend the dataset by adding newly published reports and rerunning the documented pipeline, thereby enabling replication and longitudinal extension of our analyses. We hope these additions make the workflow transparent and allow others to inspect, replicate, and build upon our work.

RESPONSES TO REVIEWER #2

Comment R2.0: *"I was very appreciative of having the opportunity to read and provide a view on this paper. I appreciated the extensive amount of work that has gone into the paper, the clarity of the description of the methods and the significance of the findings. I was also very keen on the open access dataset that will accompany the paper: this will be a hugely valuable resource for researchers within and beyond the corporate accountability field."*

Response R2.0: Thank you. We are glad to hear that you consider our findings and dataset valuable for researchers.

Comment R2.1: *"There are two minor points that I would like to see addressed in the paper. First, some more detail on the LLM used to create the core data would be helpful. For example, if this is a dedicated model developed for this work or some external model. I think readers will be really interested in knowing about this – apologies if it is tucked away in SMs and I missed it."*

Response R2.1: Thank you. To improve clarity for readers, we have added a brief statement in the Methods section (ML framework, Step 4: Generation) that specifies the provenance and use of the LLM component in our pipeline:

> "`Llama-3.1-70B-Instruct` is an externally developed, instruction-tuned large language model with publicly released weights. We did not update model parameters on our corpus, and all outputs were generated via prompting conditioned on the retrieved report chunks."

In addition, we now include an LLM reporting checklist based on GUIDE-LLM (http://www.llm-checklist.com/llm-checklist.pdf), a consensus-based reporting framework developed with input from more than 60 experts. This gives readers a structured overview of how the LLM was used, why key methodological choices were made, and what steps were taken to support responsible research practice. The completed checklist is provided in our public code repository.

Comment R2.2: *"The second point is a handful of places in the text where 'improvements or 'negative' trends are noted in disclosure. I would prefer that these are increases or decreases rather than descriptions associated with value judgements. Most readers would agree with your judgement, but it should be left to them to decide how they understand an increase/decrease."*

Response R2.2: Thank you for this helpful suggestion. We have revised the manuscript to use neutral, descriptive language throughout when reporting temporal patterns in disclosure. Specifically, we consistently refer to "increases/decreases" rather than "improvements/negative trends". Where interpretation is relevant, we now explicitly separate descriptive results from normative evaluation and phrase potential implications as empirically grounded considerations rather than value judgements, leaving their interpretation to the reader.

References

Bochkay, Khrystyna; Brown, Stephen V.; Leone, Andrew J.; Tucker, Jennifer Wu (2023): Textual analysis in accounting: What's next? In *Contemporary Accounting Research* 40 (2), pp. 765–805. doi: 10.1111/1911-3846.12825.

European Commission (2019): Commission Delegated Regulation (EU) 2018/815 of 17 December 2018 supplementing Directive 2004/109/EC of the European Parliament and of the Council with regard to regulatory technical standards on the specification of a single electronic reporting format (text with EEA relevance). In *Official Journal of the European Union* L 143, pp. 1–792.

European Financial Reporting Advisory Group (EFRAG): Digital reporting with XBRL. URL https://www.efrag.org/en/sustainability-reporting/esrs-workstreams/digital-reporting-with-xbrl.

European Parliament and the Council of the European Union (2022): Directive (EU) 2022/2464 of the European Parliament and of the Council of 14 December 2022 amending Regulation (EU) No 537/2014, Directive 2004/109/EC, Directive 2006/43/EC and Directive 2013/34/EU, as regards corporate sustainability reporting (text with EEA relevance). In *Official Journal of the European Union* L 322, pp. 15–80.

European Parliament and the Council of the European Union (2023): Regulation (EU) 2023/2859 of the European Parliament and of the Council of 13 December 2023 establishing a European single access point providing centralised access to publicly available information of relevance to financial services, capital markets and sustainability (text with EEA relevance). In *Official Journal of the European Union*.

Grishman, Ralph (2019): Twenty-five years of information extraction. In *Natural Language Engineering* 25 (6), pp. 677–692. doi: 10.1017/S1351324919000512.

Lavelli, Alberto; Califf, Mary Elaine; Ciravegna, Fabio; Freitag, Dayne; Giuliano, Claudio; Kushmerick, Nicholas; Romano, Lorenza; Ireson, Neil (2008): Evaluation of machine learning-based information extraction algorithms: criticisms and recommendations. In *Language Resources and Evaluation* 42 (4), pp. 361–393. doi: 10.1007/s10579-008-9079-3.

London Stock Exchange Group (2025): ESG data. URL https://www.lseg.com/en/data-analytics/financial-data/company-data/esg-data

Loughran, Tim; McDonald, Bill (2016): Textual analysis in accounting and finance: a survey. In *Journal of Accounting Research* 54 (4), pp. 1187–1230. doi: 10.1111/1475-679X.12123.

Xu, Derong; Chen, Wei; Peng, Wenjun; Zhang, Chao; Xu, Tong; Zhao, Xiangyu; Wu, Xian; Zheng, Yefeng; Wang, Yang; Chen, Enhong (2024): Large language models for generative information extraction: a survey. In *Frontiers of Computer Science* 18, Article 186357. doi: 10.1007/s11704-024-40555-y
